# Supplementary material for: GastritisMIL: An interpretable deep learning model for the comprehensive histological assessment of chronic gastritis
Source: Patterns (N Y). 2025 Jun 10;6(8):101286. doi: 10.1016/j.patter.2025.101286 (PMC12365534; doi:10.1016/j.patter.2025.101286)
Supplement: Document S1. Figures S1–S6 and Tables S1–S14 [file mmc1.pdf]

Patterns, Volume *6*

Supplemental information

GastritisMIL: An interpretable deep learning model for the comprehensive histological assessment of chronic gastritis

Kun Xia, Yihuang Hu, Shuntian Cai, Mengjie Lin, Mingzhi Lu, Huadong Lu, Yuhan Ye, Fenglian Lin, Liang Gao, Qingan Xia, Ruihua Tian, Weiping Lin, Lei Xie, Decheng Tan, Yapi Lu, Xunting Lin, Xiaoning Yang, Lingfeng Zhong, Lei Xu, Zhixin Zhang, Liansheng Wang, Jianlin Ren, and Hongzhi Xu

Supplemental Figures

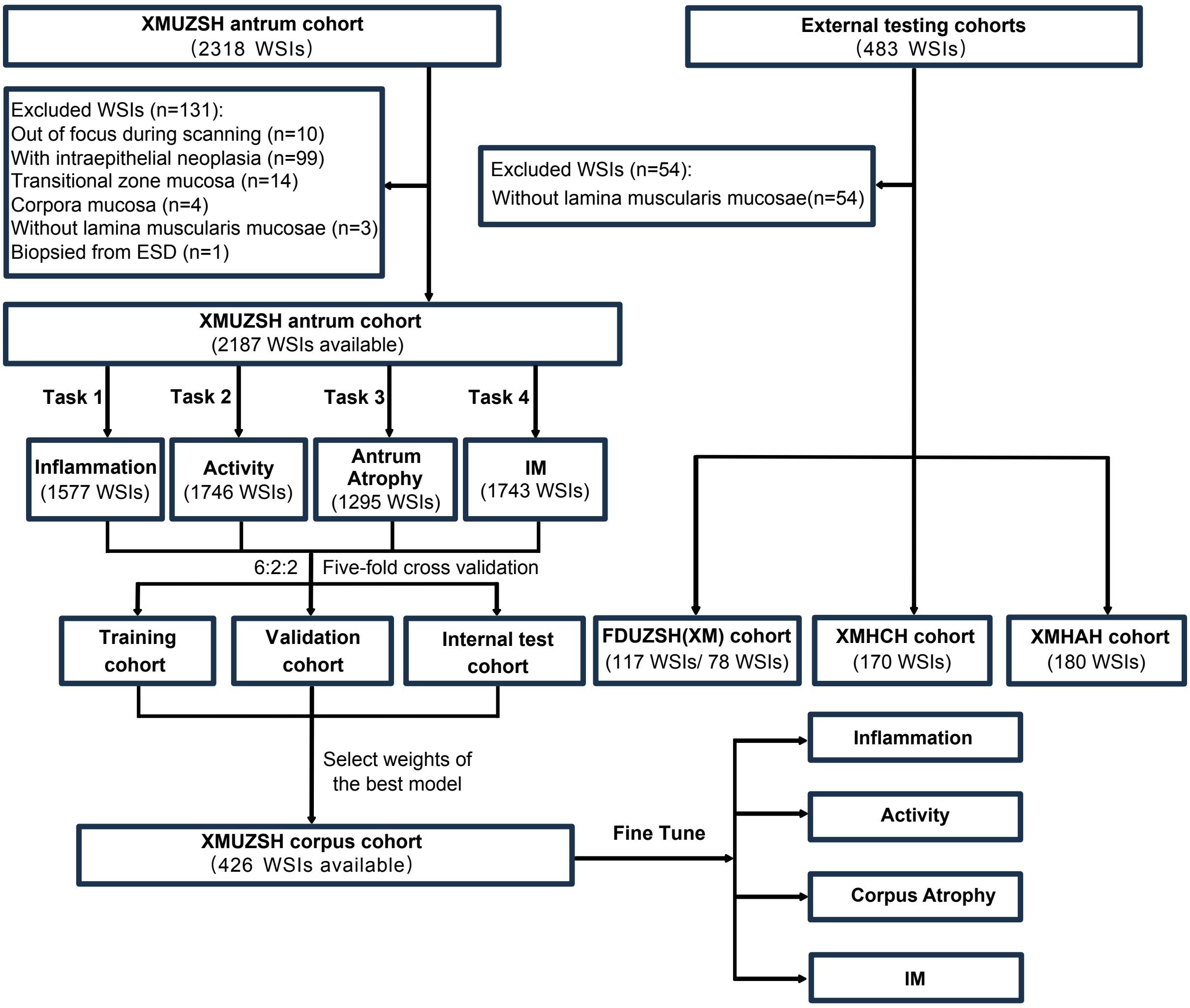

Figure S1. Overview of the study design. WSIs = whole slide images. XMUZSH = Zhongshan Hospital of the Xiamen University. FDUZSH(XM) = Zhongshan Hospital, Fudan University (Xiamen Branch). XMHCH = Xiamen Haicang Hospital. XMHAH = Xiamen Humanity Hospital. IM = Intestinal Metaplasia.

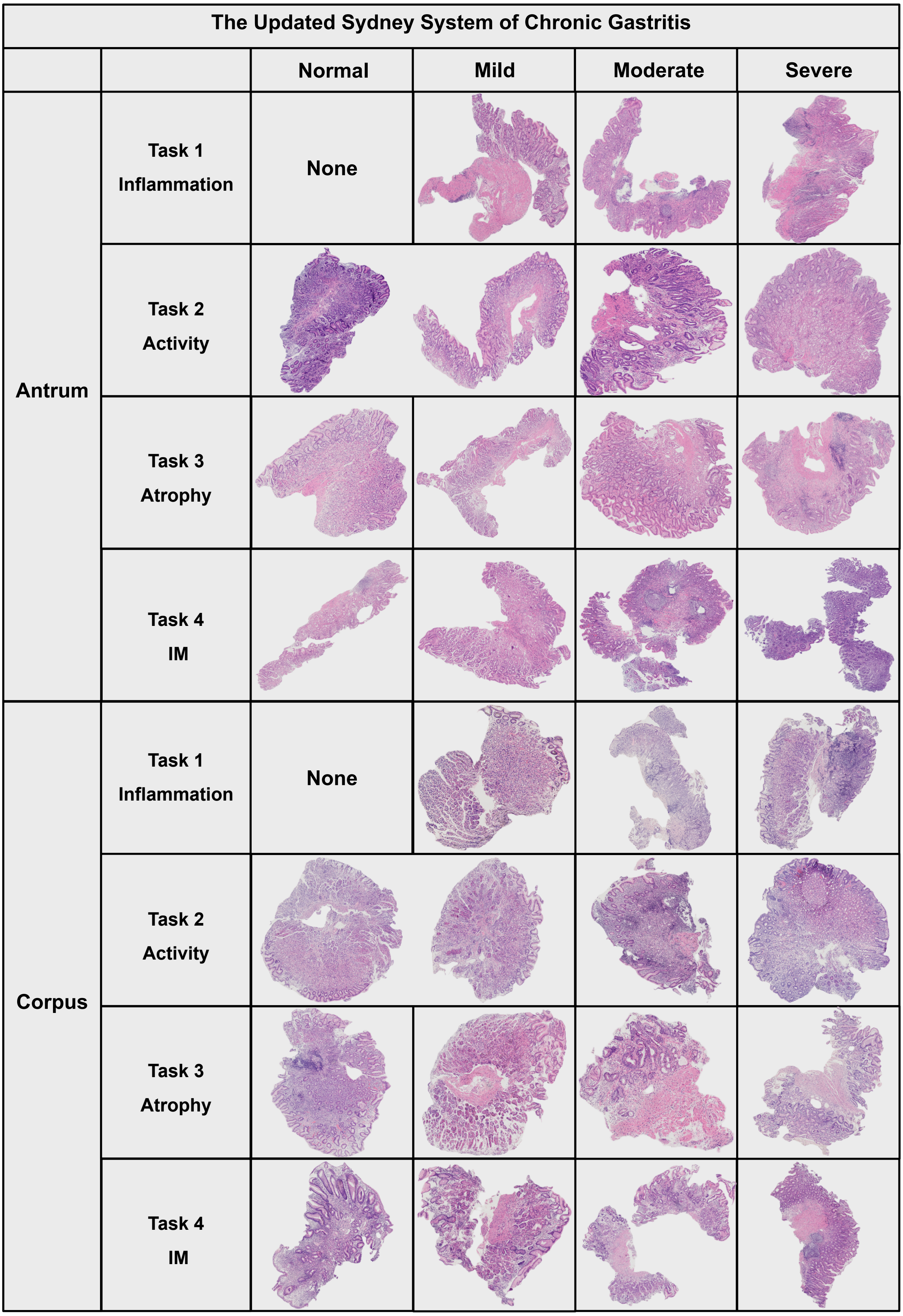

Figure S2. Detailed examples of the updated Sydney system for chronic gastritis.

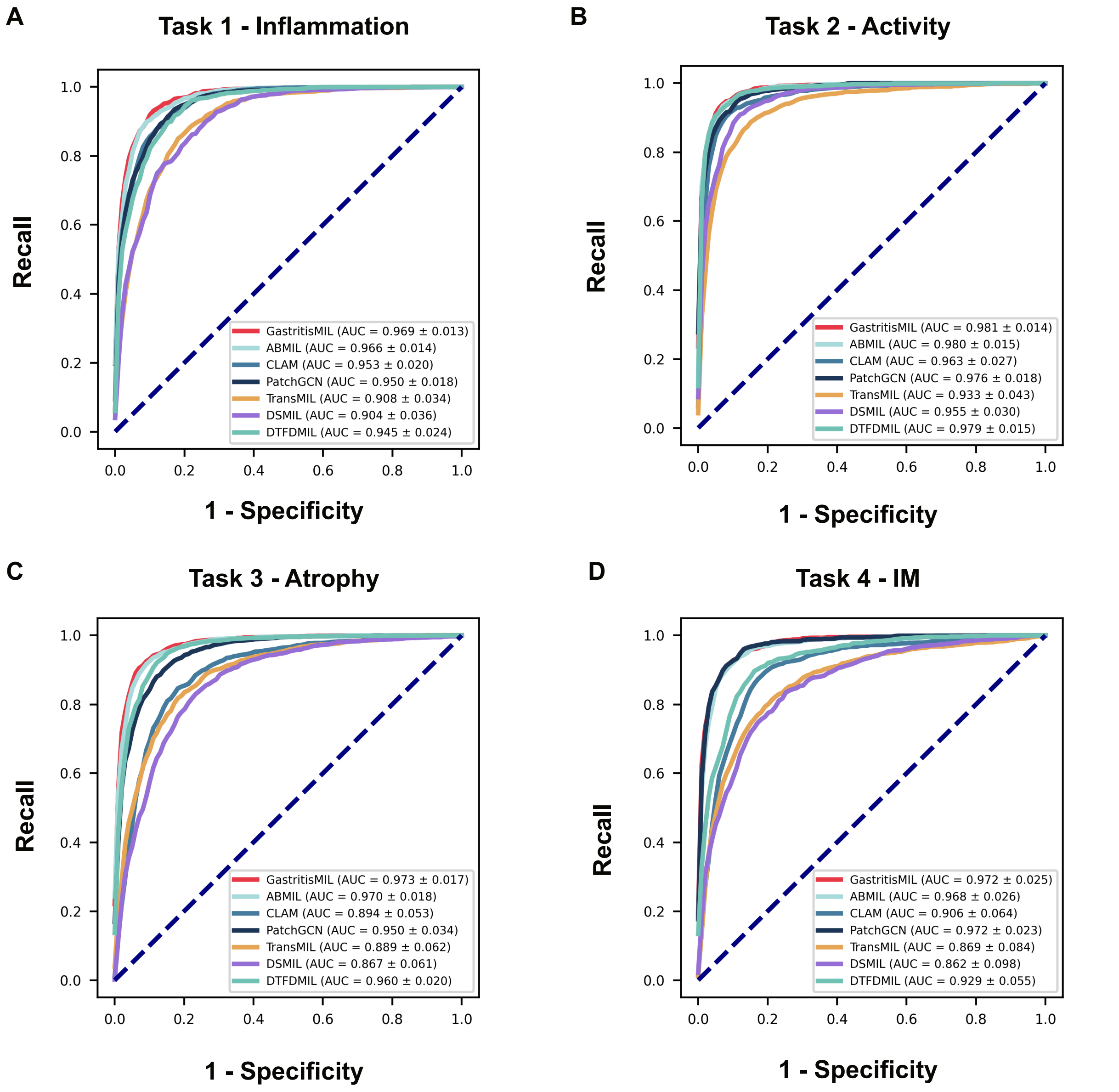

Figure S3. Comparison of ROC curves between GastritisMIL and another five frameworks utilizing ResNet50 at 40x magnification (ABMIL, CLAM, PatchGCN, TransMIL, DSMIL and DTFDMIL.)

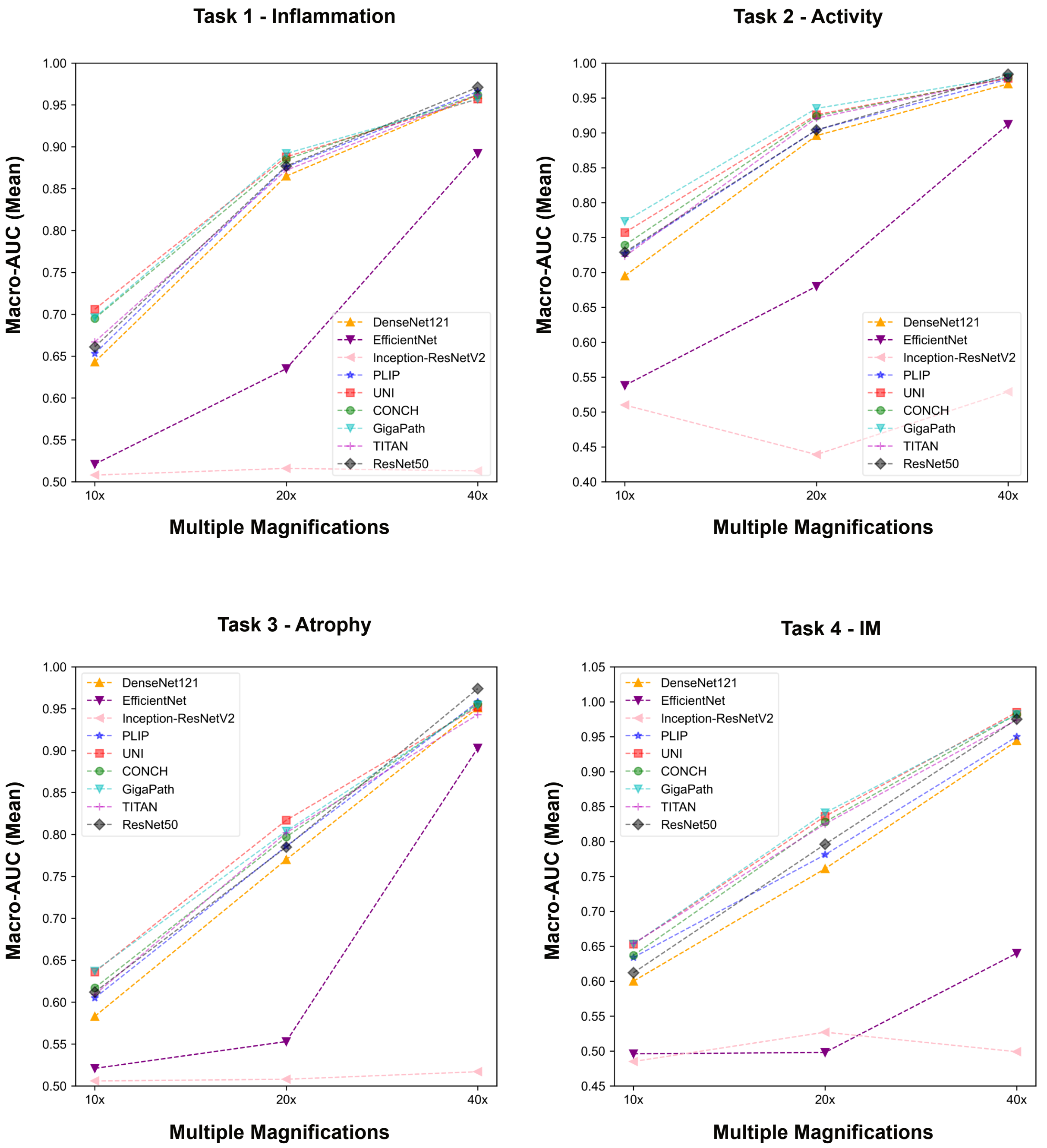

Figure S4. Comparison of Macro-AUC (Mean) among different feature extraction backbones (DenseNet121, EfficientNet, Inception-ResNetV2, PLIP, UNI, CONCH, GigaPath, TITAN and ResNet50) at multiple magnifications (10x, 20x and 40x).

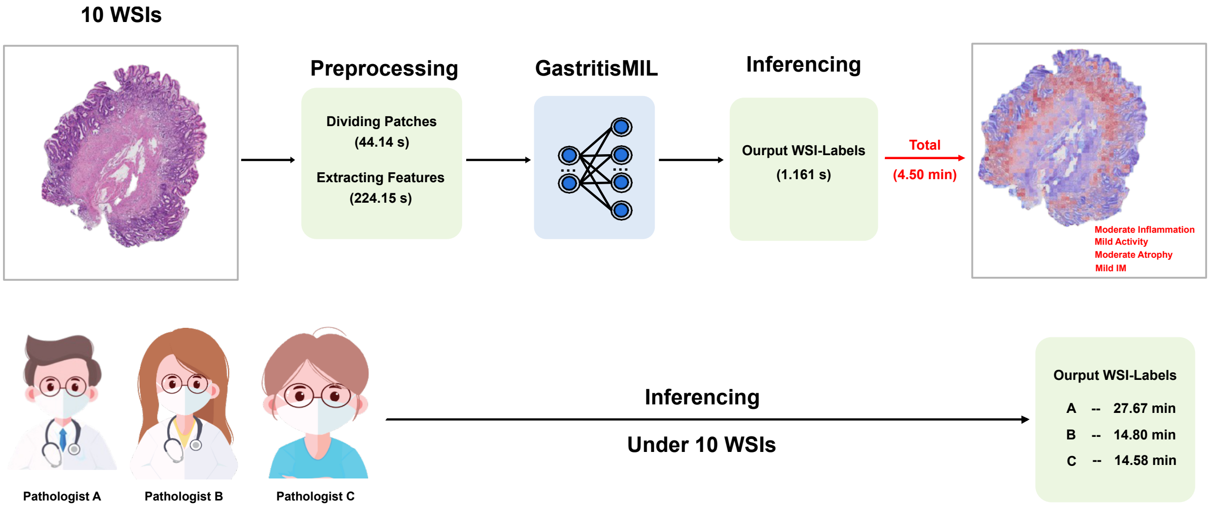

Figure S5. Comparison of judgement time on an independent external test set from 10 WSIs.

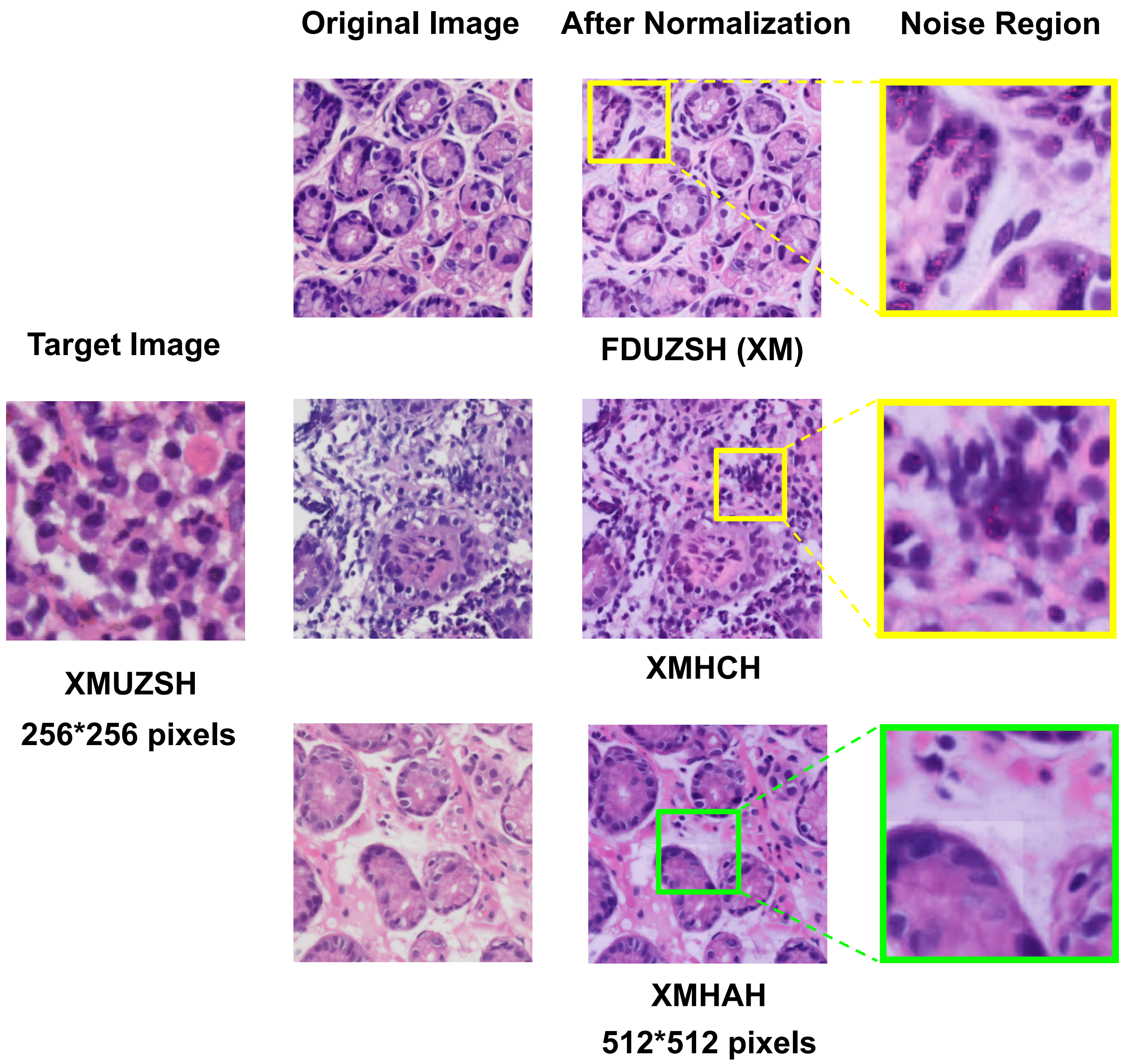

Figure S6. Comparison of the effect of coloring normalization (Macenko method) on our dataset. The green box represents noticeable tiling artifacts. The yellow box represents significant noise in some scaled images.

Supplemental Tables

XMUZSH Antrum Cohort	Task 1 - Inflammation	Task 2 - Activity	Task 3 - Antrum Atrophy	Task 4 - IM
**AUC**	0.971 ± 0.003	0.984 ± 0.003	0.974 ± 0.003	0.975 ± 0.003
**Accuracy**	0.892 ± 0.006	0.922 ± 0.006	0.866 ± 0.009	0.887 ± 0.005
**Precision**	0.842 ± 0.018	0.898 ± 0.012	0.872 ± 0.012	0.850 ± 0.010
**Recall**	0.824 ± 0.006	0.898 ± 0.010	0.834 ± 0.018	0.808 ± 0.010
**F1-score**	0.832 ± 0.009	0.900 ± 0.010	0.846 ± 0.013	0.818 ± 0.008
**CUI+**	0.706 ± 0.013	0.812 ± 0.017	0.725 ± 0.020	0.691 ± 0.013
**CUI-**	0.765 ± 0.007	0.860 ± 0.012	0.794 ± 0.020	0.761 ± 0.012
**XMUZSH Corpus Cohort**	**Task 1 - Inflammation**	**Task 2 - Activity**	**Task 3 - Corpus Atrophy**	**Task 4 - IM**
**AUC**	0.980 ± 0.008	0.977 ± 0.008	0.977 ± 0.005	0.989 ± 0.005
**Accuracy**	0.906 ± 0.013	0.942 ± 0.010	0.920 ± 0.009	0.962 ± 0.004
**Precision**	0.900 ± 0.006	0.910 ± 0.025	0.852 ± 0.019	0.890 ± 0.018
**Recall**	0.878 ± 0.045	0.874 ± 0.040	0.832 ± 0.041	0.786 ± 0.021
**F1-score**	0.872 ± 0.030	0.870 ± 0.034	0.830 ± 0.032	0.790 ± 0.024
**CUI+**	0.783 ± 0.045	0.794 ± 0.047	0.719 ± 0.049	0.685 ± 0.029
**CUI-**	0.811 ± 0.051	0.826 ± 0.035	0.788 ± 0.042	0.777 ± 0.023

Table S1. Five-fold cross-validation performance of GastritisMIL on the XMUZSH antrum cohort and the fine-tuned model on the XMUZSH corpus cohort.
Data are represented as mean ± SD. AUC = area under the receiver-operating characteristic curve. CUI+= positive clinical utility index. CUI- = negative clinical utility index.

Pathology feature extraction backbone - PLIP

Method	Task 1 - Inflammation			Task 2 - Activity		
	AUC	ACC	F1-score	AUC	ACC	F1-score
**CLAM-SB**	0.961 ± 0.003	0.874 ± 0.004	0.776 ± 0.013	0.975 ± 0.004	0.893 ± 0.010	0.850 ± 0.017
**DSMIL**	0.934 ± 0.006	0.848 ± 0.006	0.726 ± 0.018	0.965 ± 0.004	0.872 ± 0.009	0.822 ± 0.014
**DTFDMIL**	0.945 ± 0.007	0.845 ± 0.004	0.610 ± 0.022	**0.979 ± 0.003**	**0.904 ± 0.005**	**0.869 ± 0.010**
**TransMIL**	0.948 ± 0.007	0.855 ± 0.007	0.762 ± 0.027	0.967 ± 0.004	0.882 ± 0.010	0.835 ± 0.014
**PatchGCN**	0.962 ± 0.002	0.874 ± 0.002	0.796 ± 0.016	0.973 ± 0.002	0.889 ± 0.007	0.850 ± 0.011
**ABMIL**	0.965 ± 0.003	0.883 ± 0.007	**0.814 ± 0.018**	0.976 ± 0.002	0.896 ± 0.006	0.857 ± 0.009
**GastritisMIL (Ours)**	**0.966 ± 0.002**	**0.883 ± 0.005**	0.806 ± 0.009	0.977 ± 0.003	0.900 ± 0.010	0.864 ± 0.015

Method	Task 3 - Antrum Atrophy			Task 4 - Intestinal Metaplasia		
	AUC	ACC	F1-score	AUC	ACC	F1-score
**CLAM-SB**	0.936 ± 0.006	0.781 ± 0.014	0.710 ± 0.024	0.909 ± 0.004	0.802 ± 0.005	0.619 ± 0.011
**DSMIL**	0.917 ± 0.004	0.730 ± 0.003	0.623 ± 0.014	0.912 ± 0.004	0.802 ± 0.007	0.643 ± 0.010
**DTFDMIL**	0.934 ± 0.006	0.769 ± 0.013	0.583 ± 0.007	0.908 ± 0.011	0.784 ± 0.008	0.575 ± 0.017
**TransMIL**	0.912 ± 0.007	0.700 ± 0.015	0.618 ± 0.036	0.924 ± 0.002	0.805 ± 0.007	0.664 ± 0.015
**PatchGCN**	0.953 ± 0.004	0.801 ± 0.006	0.776 ± 0.011	**0.959 ± 0.003**	**0.881 ± 0.003**	**0.804 ± 0.005**
**ABMIL**	0.957 ± 0.004	**0.822 ± 0.011**	**0.793 ± 0.019**	0.953 ± 0.003	0.854 ± 0.010	0.753 ± 0.018
**GastritisMIL (Ours)**	**0.958 ± 0.005**	0.805 ± 0.014	0.753 ± 0.034	0.950 ± 0.003	0.846 ± 0.008	0.753 ± 0.019

Pathology feature extraction backbone - GigaPath

Method	Task 1 - Inflammation			Task 2 - Activity		
	AUC	ACC	F1-score	AUC	ACC	F1-score
**CLAM-SB**	**0.960 ± 0.003**	0.856 ± 0.005	0.760 ± 0.016	0.978 ± 0.002	0.904 ± 0.007	0.860 ± 0.010
**DSMIL**	0.951 ± 0.005	0.856 ± 0.005	0.738 ± 0.017	0.975 ± 0.003	0.906 ± 0.006	0.875 ± 0.008
**DTFDMIL**	0.937 ± 0.006	0.832 ± 0.006	0.690 ± 0.031	0.981 ± 0.003	**0.918 ± 0.010**	**0.882 ± 0.017**
**TransMIL**	0.880 ± 0.005	0.788 ± 0.005	0.602 ± 0.024	0.958 ± 0.007	0.868 ± 0.012	0.814 ± 0.018
**PatchGCN**	0.959 ± 0.004	**0.866 ± 0.002**	0.750 ± 0.024	0.981 ± 0.003	0.912 ± 0.008	0.874 ± 0.012
**ABMIL**	0.959 ± 0.005	0.854 ± 0.013	0.762 ± 0.015	**0.983 ± 0.003**	0.910 ± 0.009	0.876 ± 0.011
**GastritisMIL (Ours)**	0.957 ± 0.005	0.866 ± 0.008	**0.796 ± 0.012**	0.980 ± 0.002	0.912 ± 0.006	0.880 ± 0.010

Method	Task 3 - Antrum Atrophy			Task 4 - Intestinal Metaplasia		
	AUC	ACC	F1-score	AUC	ACC	F1-score
**CLAM-SB**	0.891 ± 0.021	0.702 ± 0.027	0.610 ± 0.047	0.910 ± 0.042	0.824 ± 0.037	0.640 ± 0.076
**DSMIL**	0.928 ± 0.005	0.753 ± 0.010	0.679 ± 0.018	0.969 ± 0.003	0.892 ± 0.007	0.790 ± 0.014
**DTFDMIL**	0.934 ± 0.003	0.776 ± 0.005	0.698 ± 0.012	0.969 ± 0.006	0.888 ± 0.007	0.776 ± 0.018
**TransMIL**	0.830 ± 0.008	0.574 ± 0.015	0.514 ± 0.025	0.946 ± 0.004	0.839 ± 0.009	0.680 ± 0.017
**PatchGCN**	0.948 ± 0.002	0.786 ± 0.004	0.760 ± 0.014	0.982 ± 0.003	0.912 ± 0.011	0.838 ± 0.017
**ABMIL**	**0.954 ± 0.003**	0.796 ± 0.010	0.770 ± 0.014	**0.983 ± 0.002**	**0.924 ± 0.005**	**0.858 ± 0.011**
**GastritisMIL (Ours)**	0.954 ± 0.004	**0.814 ± 0.010**	**0.796 ± 0.014**	0.982 ± 0.001	0.918 ± 0.008	0.852 ± 0.014

Table S2. Diagnostic performance of GastritisMIL in XMUZSH antrum cohort utilizing PLIP and GigaPath (internal test cohort) at 40x magnification.

Data are represented as mean ± SD. The best values are **highlighted**. The second-best values are underlined. AUC = area under the receiver-operating characteristic curve.

Comparison of different pathology feature extraction backbones at 40x								
**Method**	**Task 1 - Inflammation**				**Task 2 - Activity**			
	**AUC**	**ACC**	**F1-score**	**P-value***	**AUC**	**ACC**	**F1-score**	**P-value***
**DenseNet121**	0.962 ± 0.002	0.870 ± 0.004	0.796 ± 0.013	0.069	0.970 ± 0.004	0.893 ± 0.010	0.851 ± 0.015	0.006
**EfficientNet**	0.892 ± 0.017	0.800 ± 0.004	0.659 ± 0.009	<0.001	0.912 ± 0.002	0.797 ± 0.007	0.698 ± 0.009	<0.001
**Inception-ResNetV2**	0.513 ± 0.028	0.608 ± 0.001	0.252 ± 0.000	<0.001	0.529 ± 0.014	0.618 ± 0.001	0.255 ± 0.000	<0.001
**PLIP**	0.966 ± 0.002	0.883 ± 0.005	0.806 ± 0.009	0.498	0.977 ± 0.003	0.900 ± 0.010	0.864 ± 0.015	0.169
**UNI**	0.957 ± 0.003	0.864 ± 0.005	0.782 ± 0.011	0.014	0.978 ± 0.004	0.911 ± 0.010	0.877 ± 0.014	0.022
**CONCH**	0.962 ± 0.003	0.880 ± 0.005	0.797 ± 0.012	0.190	0.979 ± 0.003	0.906 ± 0.008	0.874 ± 0.011	0.227
**GigaPath**	0.957 ± 0.005	0.866 ± 0.008	0.796 ± 0.012	<0.001	0.980 ± 0.002	0.912 ± 0.006	0.880 ± 0.010	0.355
**TITAN**	0.962 ± 0.004	0.877 ± 0.005	0.796 ± 0.016	0.082	0.979 ± 0.002	0.907 ± 0.006	0.872 ± 0.011	0.380
**ResNet50**	**0.971 ± 0.003**	**0.892 ± 0.006**	**0.832 ± 0.009**	**reference**	**0.984 ± 0.003**	**0.922 ± 0.006**	**0.900 ± 0.010**	**reference**
**Method**	**Task 3 - Antrum Atrophy**				**Task 4 - Intestinal Metaplasia**			
	**AUC**	**ACC**	**F1-score**	**P-value***	**AUC**	**ACC**	**F1-score**	**P-value***
**DenseNet121**	0.951 ± 0.004	0.795 ± 0.010	0.752 ± 0.022	0.006	0.944 ± 0.004	0.839 ± 0.002	0.730 ± 0.006	<0.001
**EfficientNet**	0.903 ± 0.004	0.702 ± 0.008	0.645 ± 0.012	<0.001	0.640 ± 0.028	0.665 ± 0.003	0.224 ± 0.016	<0.001
**Inception-ResNetV2**	0.517 ± 0.010	0.389 ± 0.001	0.140 ± 0.000	<0.001	0.499 ± 0.016	0.664 ± 0.000	0.200 ± 0.000	<0.001
**PLIP**	0.958 ± 0.005	0.805 ± 0.014	0.753 ± 0.034	0.028	0.950 ± 0.003	0.846 ± 0.008	0.753 ± 0.019	<0.001
**UNI**	0.952 ± 0.006	0.796 ± 0.017	0.765 ± 0.023	<0.001	**0.985 ± 0.003**	0.931 ± 0.009	0.867 ± 0.015	0.024
**CONCH**	0.956 ± 0.004	0.797 ± 0.012	0.772 ± 0.007	0.008	0.982 ± 0.003	0.917 ± 0.007	0.852 ± 0.009	0.211
**GigaPath**	0.954 ± 0.004	0.814 ± 0.010	0.796 ± 0.014	<0.001	0.982 ± 0.001	**0.918 ± 0.008**	**0.852 ± 0.014**	0.376
**TITAN**	0.943 ± 0.006	0.775 ± 0.020	0.753 ± 0.023	<0.001	0.974 ± 0.002	0.905 ± 0.005	0.826 ± 0.008	0.843
**ResNet50**	**0.974 ± 0.003**	**0.866 ± 0.009**	**0.846 ± 0.013**	**reference**	0.975 ± 0.003	0.887 ± 0.005	0.818 ± 0.008	**reference**

Table S3. Diagnostic performance of GastritisMIL in XMUZSH antrum cohort utilizing different pathology feature extraction backbones (internal test cohort) at 40x magnification.

Data are represented as mean ± SD. The best values are **highlighted**. The second-best values are underlined. The P-value* indicates the difference in AUC between ResNet50 and another backbone, as determined by the DeLong's test. AUC = area under the receiver-operating characteristic curve.

Comparison of different magnification for feature extraction						
Magification	Task 1 - Inflammation			Task 2 - Activity		
	AUC	ACC	F1-score	AUC	ACC	F1-score
10x	0.661 ± 0.022	0.644 ± 0.006	0.380 ± 0.004	0.729 ± 0.013	0.674 ± 0.006	0.475 ± 0.013
20x	0.877 ± 0.006	0.765 ± 0.009	0.589 ± 0.024	0.904 ± 0.003	0.798 ± 0.008	0.709 ± 0.013
40x	**0.971 ± 0.003**	**0.892 ± 0.006**	**0.832 ± 0.009**	**0.984 ± 0.003**	**0.922 ± 0.006**	**0.900 ± 0.010**
Magification	Task 3 - Antrum Atrophy			Task 4 - Intestinal Metaplasia		
	AUC	ACC	F1-score	AUC	ACC	F1-score
10x	0.612 ± 0.020	0.449 ± 0.007	0.272 ± 0.010	0.612 ± 0.011	0.664 ± 0.005	0.284 ± 0.008
20x	0.785 ± 0.008	0.574 ± 0.022	0.441 ± 0.034	0.796 ± 0.007	0.729 ± 0.002	0.468 ± 0.008
40x	**0.974 ± 0.003**	**0.866 ± 0.009**	**0.846 ± 0.013**	**0.975 ± 0.003**	**0.887 ± 0.005**	**0.818 ± 0.008**

Table S4. Diagnostic performance of GastritisMIL in XMUZSH antrum cohort utilizing different magnification (internal test cohort).

Data are represented as mean ± SD. The best values are **highlighted**. The second-best values are underlined. AUC = area under the receiver-operating characteristic curve.

External Testing		AUC	Accuracy	F1-score	Precision	Recall	NPV
Task 1 - Inflammation	FDUZSH (XM)	0.937 ± 0.018	0.880 ± 0.029	0.914 ± 0.021	0.936 ± 0.015	0.912 ± 0.020	1.000 ± 0.085
	XMHCH	0.924 ± 0.017	0.829 ± 0.029	0.812 ± 0.035	0.877 ± 0.027	0.775 ± 0.039	0.940 ± 0.022
	XMHAH	0.913 ± 0.017	0.806 ± 0.030	0.631 ± 0.050	0.880 ± 0.114	0.633 ± 0.033	0.921 ± 0.068
Task 2 - Activity	FDUZSH (XM)	0.931 ± 0.018	0.829 ± 0.034	0.652 ± 0.061	0.621 ± 0.065	0.736 ± 0.040	1.000 ± 0.117
	XMHCH	0.923 ± 0.015	0.824 ± 0.029	0.785 ± 0.035	0.804 ± 0.034	0.800 ± 0.031	1.000 ± 0.064
	XMHAH	0.901 ± 0.017	0.800 ± 0.029	0.738 ± 0.036	0.754 ± 0.037	0.751 ± 0.032	1.000 ± 0.069
Task 3 - Atrophy	FDUZSH (XM)	0.938 ± 0.019	0.821 ± 0.044	0.811 ± 0.049	0.810 ± 0.052	0.814 ± 0.049	0.940 ± 0.037
	XMHCH	0.865 ± 0.024	0.735 ± 0.034	0.693 ± 0.051	0.828 ± 0.046	0.664 ± 0.044	0.952 ± 0.049
	XMHAH	0.883 ± 0.018	0.750 ± 0.031	0.725 ± 0.037	0.782 ± 0.031	0.731 ± 0.034	0.831 ± 0.053
Task 4 - IM	FDUZSH (XM)	0.952 ± 0.017	0.833 ± 0.042	0.816 ± 0.047	0.814 ± 0.048	0.819 ± 0.048	0.952 ± 0.008
	XMHCH	0.908 ± 0.016	0.812 ± 0.030	0.647 ± 0.065	0.754 ± 0.057	0.604 ± 0.064	0.970 ± 0.042
	XMHAH	0.928 ±0.015	0.800 ± 0.031	0.719 ± 0.040	0.725 ± 0.040	0.728 ± 0.042	0.924 ± 0.019

Table S5. AUC, accuracy, F1-score, precision, recall, NPV of our model (GastritisMIL) in the three external cohorts with images at 40x magnification.

Data are represented as mean ± SD. AUC = area under the receiver-operating characteristic curve

External Testing		Accuracy	F1-score	Precision	Recall	P-value	Kappa#
Task 1 - Inflammation	**GastritisMIL**	0.880 ± 0.029	0.914 ± 0.021	0.936 ± 0.015	0.912 ± 0.020	reference	reference
	**Junior Pathologist A**	0.803 ± 0.037	0.707 ± 0.087	0.773 ± 0.112	0.686 ± 0.084	0.188	0.526
	**Expert Pathologist B**	0.846 ± 0.034	0.792 ± 0.059	0.768 ± 0.056	0.898 ± 0.036	0.597	0.634
	**Expert Pathologist C**	0.872 ± 0.031	0.762 ± 0.072	0.743 ± 0.074	0.791 ± 0.087	1.000	0.656
Task 2 - Activity	**GastritisMIL**	0.829 ± 0.034	0.652 ± 0.059	0.621 ± 0.063	0.736 ± 0.037	reference	reference
	**Junior Pathologist A**	0.803 ± 0.039	0.397 ± 0.041	0.397 ± 0.051	0.405 ± 0.037	0.678	0.357
	**Expert Pathologist B**	0.906 ± 0.027	0.802 ± 0.069	0.816 ± 0.069	0.814 ± 0.071	0.122	0.714
	**Expert Pathologist C**	0.932 ± 0.023	0.875 ± 0.055	0.895 ± 0.037	0.879 ± 0.060	0.029	0.724
Task 3 - Atrophy	**GastritisMIL**	0.821 ± 0.043	0.811 ± 0.048	0.810 ± 0.050	0.814 ± 0.048	reference	reference
	**Junior Pathologist A**	0.641 ± 0.055	0.647 ± 0.056	0.634 ± 0.056	0.695 ± 0.051	0.038	0.719
	**Expert Pathologist B**	0.897 ± 0.034	0.908 ± 0.033	0.903 ± 0.036	0.915 ± 0.032	0.263	0.825
	**Expert Pathologist C**	0.872 ± 0.037	0.865 ± 0.043	0.868 ± 0.043	0.872 ± 0.040	0.503	0.825
Task 4 - IM	**GastritisMIL**	0.833 ± 0.042	0.816 ± 0.047	0.814 ± 0.048	0.819 ± 0.048	reference	reference
	**Junior Pathologist A**	0.833 ± 0.044	0.803 ± 0.049	0.819 ± 0.045	0.815 ± 0.044	1.000	0.878
	**Expert Pathologist B**	0.833 ± 0.043	0.806 ± 0.049	0.810 ± 0.049	0.812 ± 0.047	1.000	0.872
	**Expert Pathologist C**	0.872 ± 0.037	0.848 ± 0.045	0.855 ± 0.044	0.850 ± 0.043	0.607	0.921

Table S6. Accuracy, F1-score, precision, recall of our model (GastritisMIL) and human Pathologists in the external test cohort of FDUZSH (XM).

Data are represented as mean ± SD.

* Difference of accuracy between GastritisMIL and human Pathologists, tested by Paired chi-square test (McNamar’s test).

# inter-observer agreement of GastritisMIL and each human Pathologist, evaluated by quadratic weighted kappa coefficient.

External Testing		Accuracy	F1-score	Precision	Recall	P-value*	Kappa#
Task 1 - Inflammation	**GastritisMIL**	0.829 ± 0.029	0.812 ± 0.035	0.877 ± 0.027	0.775 ± 0.039	reference	reference
	**Junior Pathologist A**	0.588 ± 0.039	0.603 ± 0.039	0.596 ± 0.041	0.616 ± 0.042	<0.001	0.497
	**Expert Pathologist B**	0.800 ± 0.030	0.800 ± 0.030	0.783 ± 0.030	0.868 ± 0.019	0.568	0.696
	**Expert Pathologist C**	0.859 ± 0.028	0.836 ± 0.034	0.852 ± 0.035	0.826 ± 0.036	0.542	0.665
Task 2 - Activity	**GastritisMIL**	0.824 ± 0.029	0.785 ± 0.035	0.804 ± 0.034	0.800 ± 0.031	reference	reference
	**Junior Pathologist A**	0.641 ± 0.038	0.498 ± 0.043	0.624 ± 0.049	0.507 ± 0.034	<0.001	0.358
	**Expert Pathologist B**	0.829 ± 0.030	0.794 ± 0.035	0.791 ± 0.035	0.798 ± 0.035	1.000	0.752
	**Expert Pathologist C**	0.853 ± 0.027	0.819 ± 0.032	0.850 ± 0.024	0.829 ± 0.030	0.568	0.718
Task 3 - Atrophy	**GastritisMIL**	0.735 ± 0.034	0.693 ± 0.051	0.828 ± 0.046	0.664 ± 0.044	reference	reference
	**Junior Pathologist A**	0.671 ± 0.035	0.549 ± 0.042	0.587 ± 0.054	0.550 ± 0.037	0.260	0.578
	**Expert Pathologist B**	0.724 ± 0.036	0.700 ± 0.042	0.685 ± 0.042	0.753 ± 0.036	0.915	0.606
	**Expert Pathologist C**	0.753 ± 0.033	0.735 ± 0.038	0.717 ± 0.038	0.782 ± 0.033	0.824	0.568
Task 4 - IM	**GastritisMIL**	0.812 ± 0.030	0.647 ± 0.067	0.754 ± 0.054	0.604 ± 0.065	reference	reference
	**Junior Pathologist A**	0.859 ± 0.026	0.560 ± 0.060	0.754 ± 0.131	0.548 ± 0.049	0.200	0.687
	**Expert Pathologist B**	0.859 ± 0.027	0.684 ± 0.060	0.692 ± 0.061	0.683 ± 0.064	0.200	0.775
	**Expert Pathologist C**	0.847 ± 0.027	0.746 ± 0.048	0.746 ± 0.044	0.821 ± 0.043	0.392	0.700

Table S7. Accuracy, F1-score, precision, recall of our model (GastritisMIL) and human Pathologists in the external test cohort of XMHCH.

Data are represented as mean ± SD.

* Difference of accuracy between GastritisMIL and human Pathologists, tested by Paired chi-square test (McNamar's test).

# inter-observer agreement of GastritisMIL and each human Pathologist, evaluated by quadratic weighted kappa coefficient.

External Testing		Accuracy	F1-score	Precision	Recall	P-value*	Kappa#
Task 1 - Inflammation	**GastritisMIL**	0.806 ± 0.030	0.631 ± 0.050	0.880 ± 0.114	0.633 ± 0.033	reference	reference
	**Junior Pathologist A**	0.794 ± 0.031	0.756 ± 0.043	0.785 ± 0.046	0.736 ± 0.047	0.892	0.536
	**Expert Pathologist B**	0.789 ± 0.031	0.706 ± 0.049	0.766 ± 0.056	0.677 ± 0.048	0.788	0.493
	**Expert Pathologist C**	0.756 ± 0.031	0.772 ± 0.034	0.773 ± 0.036	0.837 ± 0.020	0.262	0.614
Task 2 - Activity	**GastritisMIL**	0.800 ± 0.029	0.738 ± 0.036	0.754 ± 0.037	0.751 ± 0.032	reference	reference
	**Junior Pathologist A**	0.650 ± 0.036	0.528 ± 0.041	0.671 ± 0.031	0.536 ± 0.035	0.003	0.403
	**Expert Pathologist B**	0.861 ± 0.026	0.831 ± 0.032	0.842 ± 0.029	0.833 ± 0.031	0.169	0.677
	**Expert Pathologist C**	0.872 ± 0.024	0.850 ± 0.028	0.841 ± 0.027	0.865 ± 0.028	0.053	0.796
Task 3 - Atrophy	**GastritisMIL**	0.750 ± 0.031	0.725 ± 0.037	0.782 ± 0.031	0.731 ± 0.034	reference	reference
	**Junior Pathologist A**	0.611 ± 0.035	0.538 ± 0.038	0.554 ± 0.041	0.552 ± 0.036	0.011	0.698
	**Expert Pathologist B**	0.778 ± 0.032	0.750 ± 0.035	0.760 ± 0.033	0.785 ± 0.029	0.635	0.691
	**Expert Pathologist C**	0.822 ± 0.029	0.799 ± 0.033	0.813 ± 0.032	0.796 ± 0.033	0.079	0.814
Task 4 - IM	**GastritisMIL**	0.800 ± 0.031	0.719 ± 0.040	0.725 ± 0.040	0.728 ± 0.042	reference	reference
	**Junior Pathologist A**	0.833 ± 0.027	0.763 ± 0.036	0.767 ± 0.036	0.811 ± 0.031	0.48	0.877
	**Expert Pathologist B**	0.878 ± 0.024	0.832 ± 0.032	0.830 ± 0.031	0.848 ± 0.033	0.059	0.868
	**Expert Pathologist C**	0.861 ± 0.026	0.808 ± 0.035	0.798 ± 0.036	0.822 ± 0.036	0.135	0.895

Table S8. Accuracy, F1-score, precision, recall of our model (GastritisMIL) and human Pathologists in the external test cohort of XMHAH.

Data are represented as mean ± SD.

* Difference of accuracy between GastritisMIL and human Pathologists, tested by Paired chi-square test (McNamar's test).

# inter-observer agreement of GastritisMIL and each human Pathologist, evaluated by quadratic weighted kappa coefficient.

Comparison of algorithm performance in recent CG-AI research								
**Research**	**Task 1 - Inflammation**				**Task 2 - Activity**			
	**Discrimination**	**AUC**	**Recall**	**Specificity**	**Discrimination**	**AUC**	**Recall**	**Specificity**
**LR**	NR	NR	NR	NR	NR	NR	NR	NR
**AMMNet**	NR	NR	NR	NR	whether activity	0.930	NR	NR
**GasMIL**	specific grade of Inflammation	0.970	0.860	0.860	specific grade of activity	0.981	0.900	0.900
**GastritisMIL (Ours)**	specific grade of Inflammation	**0.971**	**0.824**	**0.940**	specific grade of activity	**0.984**	**0.898**	**0.967**
**Research**	**Task 3 - Antrum Atrophy**				**Task 4 - Intestinal Metaplasia**			
	**Discrimination**	**AUC**	**Recall**	**Specificity**	**Discrimination**	**AUC**	**Recall**	**Specificity**
**LR**	moderate or severe atrophy	0.818	0.724	0.896	NR	NR	NR	NR
**AMMNet**	whether atrophy	0.970	NR	NR	whether IM	0.940	NR	NR
**GasMIL**	specific grade of atrophy	0.877	0.700	0.700	specific grade of IM	0.884	0.690	0.690
**GastritisMIL (Ours)**	specific grade of atrophy	**0.974**	**0.834**	**0.960**	specific grade of IM	**0.975**	**0.808**	**0.953**

Table S9. Comparison of algorithm performance in recent CG-AI research.
AUC = area under the receiver-operating characteristic curve. NR = not report.

Age-Stratified Performance Analysis Across Internal and External Cohorts								
**Age Group**	**Task 1 - Inflammation**				**Task 2 - Activity**			
	**XMUZSH (Internal)**	**FDUZSH (XM)**	**XMHCH**	**XMHAH**	**XMUZSH (Internal)**	**FDUZSH (XM)**	**XMHCH**	**XMHAH**
**Age < 45**	0.913 (24.9%)	0.889 (15.4%)	0.868 (31.2%)	0.818 (36.7%)	0.923 (52.0%)	0.833 (15.4%)	0.792 (31.2%)	0.909 (36.7%)
**45 ≤ Age ≤ 60**	0.886 (50.2%)	0.873 (53.8%)	0.822 (42.9%)	0.778 (35.0%)	0.925 (48.3%)	0.857 (53.8%)	0.781 (42.9%)	0.730 (35.0%)
**Age > 60**	0.880 (24.9%)	0.889 (30.7%)	0.795 (25.9%)	0.824 (28.3%)	0.916 (26.5%)	0.778 (30.7%)	0.909 (25.9%)	0.745 (28.3%)
**Average**	0.892	0.880	0.829	0.806	0.922	0.829	0.824	0.800
**Age Group**	**Task 3 - Antrum Atrophy**				**Task 4 - Intestinal Metaplasia**			
	**XMUZSH (Internal)**	**FDUZSH (XM)**	**XMHCH**	**XMHAH**	**XMUZSH (Internal)**	**FDUZSH (XM)**	**XMHCH**	**XMHAH**
**Age < 45**	0.872 (26.0%)	0.938 (20.5%)	0.660 (31.2%)	0.727 (36.7%)	0.896 (24.8%)	0.750 (20.5%)	0.849 (31.2%)	0.803 (36.7%)
**45 ≤ Age ≤ 60**	0.843 (47.8%)	0.811 (47.4%)	0.753 (42.9%)	0.746 (35.0%)	0.878 (48.7%)	0.892 (47.4%)	0.767 (42.9%)	0.857 (35.0%)
**Age > 60**	0.900 (26.2%)	0.760 (32.1%)	0.795 (25.9%)	0.784 (28.3%)	0.896 (26.4%)	0.800 (32.1%)	0.841 (25.9%)	0.725 (28.3%)
**Average**	0.866	0.821	0.735	0.750	0.896	0.833	0.812	0.800

Table S10. Age-stratified performance analysis across internal and three external cohorts.
Data are represented as accuracy (Proportion of the corresponding age group).

Option	External Testing		AUC	Accuracy	F1-score	Precision	Recall	NPV
Without Staining Normalization	Task 1 - Inflammation	FDUZSH (XM)	0.937 ± 0.018	0.880 ± 0.029	0.914 ± 0.021	0.936 ± 0.015	0.912 ± 0.020	1.000 ± 0.085
		XMHCH	0.924 ± 0.017	0.829 ± 0.029	0.812 ± 0.035	0.877 ± 0.027	0.775 ± 0.039	0.940 ± 0.022
		XMHAH	0.913 ± 0.017	0.806 ± 0.030	0.631 ± 0.050	0.880 ± 0.114	0.633 ± 0.033	0.921 ± 0.068
	Task 2 - Activity	FDUZSH (XM)	0.931 ± 0.018	0.829 ± 0.034	0.652 ± 0.061	0.621 ± 0.065	0.736 ± 0.040	1.000 ± 0.117
		XMHCH	0.923 ± 0.015	0.824 ± 0.029	0.785 ± 0.035	0.804 ± 0.034	0.800 ± 0.031	1.000 ± 0.064
		XMHAH	0.901 ± 0.017	0.800 ± 0.029	0.738 ± 0.036	0.754 ± 0.037	0.751 ± 0.032	1.000 ± 0.069
	Task 3 - Atrophy	FDUZSH (XM)	0.938 ± 0.019	0.821 ± 0.044	0.811 ± 0.049	0.810 ± 0.052	0.814 ± 0.049	0.940 ± 0.037
		XMHCH	0.865 ± 0.024	0.735 ± 0.034	0.693 ± 0.051	0.828 ± 0.046	0.664 ± 0.044	0.952 ± 0.049
		XMHAH	0.883 ± 0.018	0.750 ± 0.031	0.725 ± 0.037	0.782 ± 0.031	0.731 ± 0.034	0.831 ± 0.053
	Task 4 - IM	FDUZSH (XM)	0.952 ± 0.017	0.833 ± 0.042	0.816 ± 0.047	0.814 ± 0.048	0.819 ± 0.048	0.952 ± 0.008
		XMHCH	0.908 ± 0.016	0.812 ± 0.030	0.647 ± 0.065	0.754 ± 0.057	0.604 ± 0.064	0.970 ± 0.042
		XMHAH	0.928 ±0.015	0.800 ± 0.031	0.719 ± 0.040	0.725 ± 0.040	0.728 ± 0.042	0.924 ± 0.019
With Staining Normalization	Task 1 - Inflammation	FDUZSH (XM)	0.913 ± 0.021	0.761 ± 0.039	0.647 ± 0.076	0.689 ± 0.083	0.645 ± 0.087	0.973 ± 0.092
		XMHCH	0.925 ± 0.015	0.794 ± 0.031	0.766 ± 0.038	0.853 ± 0.024	0.729 ± 0.039	0.916 ± 0.032
		XMHAH	0.916 ± 0.017	0.744 ± 0.033	0.590 ± 0.049	0.857 ± 0.111	0.594 ± 0.033	0.921 ± 0.103
	Task 2 - Activity	FDUZSH (XM)	0.911 ± 0.028	0.829 ± 0.035	0.685 ± 0.068	0.674 ± 0.072	0.702 ± 0.074	0.972 ± 0.135
		XMHCH	0.911 ± 0.016	0.776 ± 0.033	0.717 ± 0.037	0.725 ± 0.038	0.735 ± 0.033	0.981 ± 0.065
		XMHAH	0.897 ± 0.018	0.783 ± 0.031	0.699 ± 0.036	0.711 ± 0.040	0.715 ± 0.030	1.000 ± 0.072
	Task 3 - Atrophy	FDUZSH (XM)	0.883 ± 0.030	0.692 ± 0.052	0.694 ± 0.056	0.696 ± 0.056	0.707 ± 0.055	0.967 ± 0.064
		XMHCH	0.803 ± 0.027	0.600 ± 0.038	0.549 ± 0.043	0.569 ± 0.043	0.554 ± 0.045	0.949 ± 0.062
		XMHAH	0.815 ± 0.020	0.556 ± 0.036	0.476 ± 0.040	0.605 ± 0.050	0.468 ± 0.034	0.884 ± 0.030
	Task 4 - IM	FDUZSH (XM)	0.883 ± 0.030	0.833 ± 0.042	0.812 ± 0.049	0.819 ± 0.048	0.820 ± 0.046	1.000 ± 0.035
		XMHCH	0.803 ± 0.027	0.718 ± 0.035	0.492 ± 0.068	0.647 ± 0.077	0.464 ± 0.062	0.964 ± 0.072
		XMHAH	0.815 ± 0.020	0.728 ± 0.033	0.632 ± 0.041	0.628 ± 0.040	0.644 ± 0.044	0.927 ± 0.030

Table S11. Impact of Staining Normalization on Model Performance Across External Test Cohorts.

Data are represented as mean ± SD. AUC = area under the receiver-operating characteristic curve

Age Distribution Analysis Across Internal and External Cohorts						
**Cohort**	**Task 1 - Inflammation**			**Task 3 - Antrum Atrophy**		
	**Age < 45**	**45 ≤ Age ≤ 60**	**Age > 60**	**Age < 45**	**45 ≤ Age ≤ 60**	**Age > 60**
**Training Cohort**	25.6%	48.8%	25.6%	23.6%	48.9%	27.5%
**Internal Test Cohort**	25.0%	50.0%	25.0%	26.0%	48.3%	25.7%
**FDUZSH (XM)**	15.4%	53.8%	30.8%	15.4%	53.8%	30.8%
**XMHCH**	31.2%	42.9%	25.9%	31.2%	42.9%	25.9%
**XMHAH**	35.7%	35.0%	28.3%	35.7%	35.0%	28.3%
**Cohort**	**Task 2 - Activity**			**Task 4 - Intestinal Metaplasia**		
	**Age < 45**	**45 ≤ Age ≤ 60**	**Age > 60**	**Age < 45**	**45 ≤ Age ≤ 60**	**Age > 60**
**Training Cohort**	25.6%	48.6%	25.7%	25.6%	49.1%	25.4%
**Internal Test Cohort**	23.9%	48.3%	27.8%	26.1%	45.8%	28.1%
**FDUZSH (XM)**	20.5%	47.4%	32.1%	20.5%	47.4%	32.1%
**XMHCH**	31.2%	42.9%	25.9%	31.2%	42.9%	25.9%
**XMHAH**	36.7%	35.0%	28.3%	36.7%	35.0%	28.3%

Table S12. Age Distribution Analysis Across Internal and External Cohorts.

Label Distribution Analysis Across Internal and External Cohorts							
**Cohort**	**Task 1 - Inflammation**			**Task 3 - Antrum Atrophy**			
	**Normal to Mild**	**Moderate**	**Severe**	**Normal**	**Mild**	**Moderate**	**Severe**
**Training Cohort**	30.9%	60.8%	8.3%	37.1%	39.0%	19.3%	4.6%
**Internal Test Cohort**	31.0%	60.8%	8.2%	37.5%	38.6%	19.3%	4.6%
**FDUZSH (XM)**	45.30%	50.4%	4.3%	15.4%	32.1%	37.2%	15.4%
**XMHCH**	29.4%	54.1%	16.5%	34.7%	32.9%	25.3%	54.1%
**XMHAH**	53.3%	37.8%	8.9%	14.4%	23.9%	21.7%	40.0%
**Cohort**	**Task 2 - Activity**			**Task 4 - Intestinal Metaplasia**			
	**Normal to Mild**	**Moderate**	**Severe**	**Normal**	**Mild**	**Moderate**	**Severe**
**Training Cohort**	61.8%	21.1%	17.1%	66.4%	14.4%	11.7%	7.5%
**Internal Test Cohort**	61.7%	21.1%	17.1%	66.5%	14.6%	11.5%	7.4%
**FDUZSH (XM)**	78.6%	14.5%	6.8%	38.5%	17.9%	23.1%	20.5%
**XMHCH**	51.8%	20.6%	27.6%	66.5%	22.4%	5.9%	5.3%
**XMHAH**	50.0%	18.9%	31.1%	49.4%	10.0%	24.4%	16.1%

Table S13. Label Distribution Analysis Across Internal and External Cohorts.

Cohort		Task 1 & Compression Error		Task 3 & Structure Confusion Error		Task 4 & Structure Confusion Error	
		Proportion	P-value*	Proportion	P-value*	Proportion	P-value*
External Cohort	FDUZSH (XM)	42.9% (6/14)	1.000	61.1% (11/18)	0.430	46.2% (6/13)	0.776
	XMHCH	44.8% (13/29)	0.757	57.8% (26/45)	0.333	45.5% (15/33)	0.450
	XMHAH	54.3% (19/35)	0.163	51.1% (23/45)	0.864	47.2% (17/36)	0.547
Internal Cohort	XMUZSH	39.8% (68/171)	reference	48.3% (84/174)	reference	54.3% (107/197)	reference

Table S14. Proportions of Error Types Among Total Failure Cases in Different Tasks Across Multi-center Cohorts.

* Difference of error proportion between XMUZSH cohort and another external cohort, tested by Chi-square test.
