# Supplementary material for: GastritisMIL: An interpretable deep learning model for the comprehensive histological assessment of chronic gastritis
Source: Patterns (N Y). 2025 Jun 10;6(8):101286. doi: 10.1016/j.patter.2025.101286 (PMC12365534; doi:10.1016/j.patter.2025.101286)
Supplement: Document S2. Article plus supplemental information [file mmc2.pdf]

Article

Patterns

GastritisMIL: An interpretable deep learning model for the comprehensive histological assessment of chronic gastritis

Graphical abstract

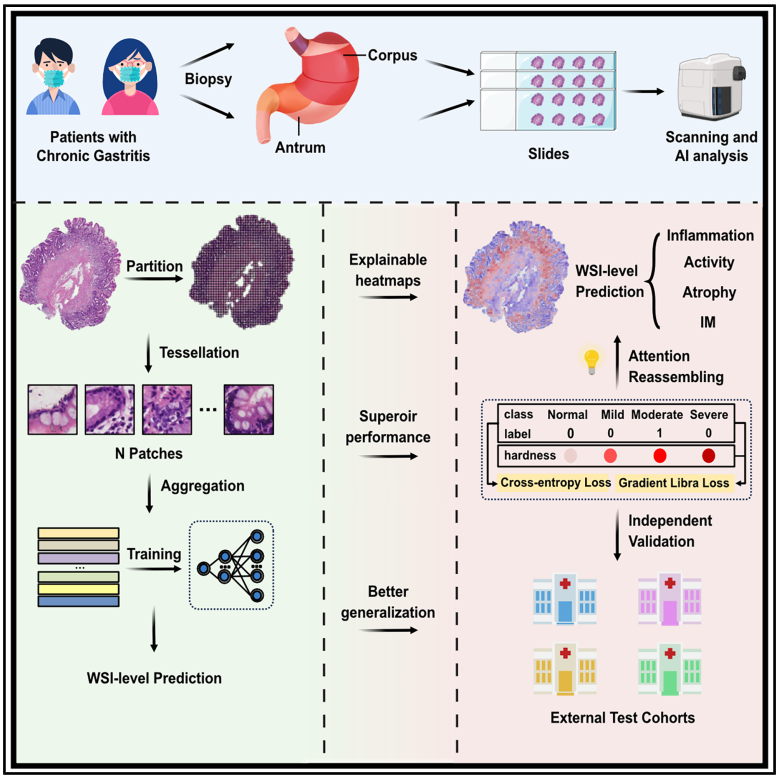

Highlights

- Harnessing DL for chronic gastritis patients' histological alterations severity
- Using visualizable heatmaps to help pathologists rapidly localize suspicious lesions
- Superior performance and high generalizability demonstrate its clinical value

Authors

Kun Xia, Yihuang Hu, Shuntian Cai, ..., Liansheng Wang, Jianlin Ren, Hongzhi Xu

Correspondence

lswang@xmu.edu.cn (L.W.),
renjianl@xmu.edu.cn (J.R.),
xuhongzhi@xmu.edu.cn (H.X.)

In brief

As the precursor to gastric cancer, the impending challenge of chronic gastritis necessitates better management. GastritisMIL provides an interpretable AI-driven model adept at comprehensively assessing histological alterations for chronic gastritis patients undergoing endoscopic biopsy. By not just solely diagnosing but also explaining localizing lesions with better generalization, the research paves the way for rapid diagnosis and personalized follow-up interventions.

Xia et al., 2025, Patterns *6*, 101286
August 8, 2025 © 2025 The Author(s). Published by Elsevier Inc.
https://doi.org/10.1016/j.patter.2025.101286

CellPress

Please cite this article in press as: Xia et al., GastritisMIL: An interpretable deep learning model for the comprehensive histological assessment of chronic gastritis, Patterns (2025), https://doi.org/10.1016/j.patter.2025.101286

Patterns

CellPress
OPEN ACCESS

Article

GastritisMIL: An interpretable deep learning model for the comprehensive histological assessment of chronic gastritis

Kun Xia,[1,14] Yihuang Hu,[2,14] Shuntian Cai,[1,3,4,5,14] Mengjie Lin,[6,14] Mingzhi Lu,[7] Huadong Lu,[8] Yuhan Ye,[6] Fenglian Lin,[6] Liang Gao,[9] Qingan Xia,[10] Ruihua Tian,[11] Weiping Lin,[2] Lei Xie,[1] Decheng Tan,[12] Yapi Lu,[1] Xunting Lin,[1] Xiaoning Yang,[1,3,4,5] Lingfeng Zhong,[1] Lei Xu,[13] Zhixin Zhang,[13] Liansheng Wang,[2,4,*] Jianlin Ren,[1,3,4,5,*] and Hongzhi Xu[1,3,4,5,15,*]

[1]Department of Gastroenterology, The National Key Clinical Specialty, Zhongshan Hospital of Xiamen University, School of Medicine, Xiamen University, Xiamen 361004, Fujian Province, P.R. China
[2]Department of Computer Science, School of Informatics, Xiamen University, Xiamen 361005, Fujian Province, P.R. China
[3]Clinical Research Center for Gut Microbiota and Digestive Diseases of Fujian Province, Xiamen Key Laboratory of Intestinal Microbiome and Human Health, Xiamen 361004, Fujian Province, P.R. China
[4]Department of Digestive Disease, Institute for Microbial Ecology, Xiamen University, Xiamen 361004, Fujian Province, P.R. China
[5]Xiamen Gastroenterology Quality Control Center, Xiamen 361004, Fujian Province, P.R. China
[6]Department of Pathology, Zhongshan Hospital of Xiamen University, School of Medicine, Xiamen University, Xiamen 361004, Fujian Province, P.R. China
[7]Department of Pathology, Xiamen Humanity Hospital, 3777 Xianyue Road Hull District, Xiamen 361006, Fujian Province, P.R. China
[8]Department of Pathology, Zhongshan Hospital (Xiamen), Fudan University, Xiamen Clinical Research Center for Cancer Therapy, Xiamen 361015, Fujian Province, P.R. China
[9]Department of Pathology, Xiamen Haicang Hospital, Xiamen 361026, Fujian Province, P.R. China
[10]Department of Pathology, TangShan GongRen Hospital, Tangshan 063003, Hebei Province, P.R. China
[11]Department of Pathology, Women and Children's Hospital, School of Medicine, Xiamen University, Xiamen 361003, P.R. China
[12]School of Life Sciences, Xiamen University, Xiamen 361002, Fujian Province, P.R. China
[13]Department of Gastroenterology, Ningbo First Hospital, Ningbo 315211, P.R. China
[14]These authors contributed equally
[15]Lead contact
*Correspondence: lswang@xmu.edu.cn (L.W.), renjianl@xmu.edu.cn (J.R.), xuhongzhi@xmu.edu.cn (H.X.)
https://doi.org/10.1016/j.patter.2025.101286

THE BIGGER PICTURE Chronic gastritis (CG) is the most common chronic digestive disease, with significant limitations in endoscopic evaluation of gastric atrophy and intestinal metaplasia (IM). While histological examination remains the diagnostic gold standard, the complexity of CG evaluation calls for innovative approaches to improve accuracy and efficiency. Notably, the early onset of atrophy of gastric mucosal glands and IM correlates with elevated grades of OLGA/OLGIM and reduced follow-up intervals, indicating an increased risk of early-stage gastric cancer. This study provides GastritisMIL, an interpretable deep learning (DL) model developed using the largest CG-related whole slide image (WSI) dataset annotated with fine-grained labels based on the updated Sydney system. Our model demonstrates superior performance in classifying histological alterations across three external test cohorts, significantly surpassing junior pathologists and aligning closely with senior pathologists' assessments. By delivering rapid and precise pathological insights, the model enhances clinicians' ability to monitor disease progression, optimize endoscopic strategies, and strengthen surveillance for high-risk patients. This work establishes a paradigm for integrating DL into clinical workflows for pathological assessment of CG, with the potential to streamline diagnostic sufficiency and improve patient outcomes.

SUMMARY

The comprehensive histological assessment of chronic gastritis is imperative for guiding endoscopic follow-up strategies and surveillance of early-stage gastric cancer, yet rapid and objective assessment remains challenging in clinical workflows. We propose a powerful deep learning model (GastritisMIL) to effectively identify pathological alterations on H&E-stained biopsy slides, thereby expediting pathologists' evaluation

© 2025 The Author(s). Published by Elsevier Inc.
This is an open access article under the CC BY license (http://creativecommons.org/licenses/by/4.0/).

and improving decision-making regarding follow-up intervals. We have trained and tested GastritisMIL by using retrospective data from 2,744 patients and evaluated discriminative performance across three medical centers (467 patients). GastritisMIL attained areas under the receiver operating curve greater than 0.971 in four tasks (inflammation, activity, atrophy, and intestinal metaplasia) and superior performance comparable to that of two senior pathologists. Specifically, interpretable attention heatmaps generated by GastritisMIL effectively assist junior pathologists in locating suspicious lesion regions across the entire field and minimizing missed diagnosis risk. Moreover, the high generalizability of this developed model across multiple external cohorts demonstrates its potential translational value.

INTRODUCTION

Chronic gastritis (CG) is widely recognized as the most common chronic digestive disease. Endoscopic assessment is the main diagnostic method (nearly 90%) for CG due to its visual immediacy and convenience.[1] However, endoscopy struggles with accurately evaluating gastric atrophy and intestinal metaplasia (IM) and requires fine-grained grading labels of alterations. Instead, histological assessment, the gold standard, provides more precise diagnostics, especially when there are discrepancies between endoscopic and microscopic findings.[2] It is found that gastroscopic diagnosis for CG is only about half as accurate as pathological examination, limiting its applicability for prolonged surveillance.[3] Early onset of atrophy and IM in the gastric mucosal glands correlates with elevated grades of the operative link for gastritis assessment (OLGA)/operative link for gastric intestinal metaplasia (OLGIM)[4–6] and reduced follow-up intervals, indicating an increased risk of early-stage gastric cancer (GC). These findings underscore the necessity for timely endoscopic surveillance, recommending intervals of no more than 3 years to monitor these high-risk patients effectively. Therefore, pathological examination has emerged as a more authoritative approach in routine clinical practice, essential for long-term monitoring and optimizing endoscopic follow-up strategies for CG patients.

Several methods has been developed to evaluate histological alterations in CG,[7–9] with recent clinical guidelines recommending the visual analog scoring method of the updated Sydney system, which integrates inflammation, activity, atrophy, and IM into the diagnostic criteria for pathology reports.[9–11] Pathologists routinely grade tissue samples from the gastric antrum, corpus, and fundus based on standardized biopsies extending to the lamina muscularis mucosae, assigning grades of normal, mild, moderate, or severe. However, the high volume of routine gastroscopic biopsies, coupled with the global shortage of anatomical pathologists, imposes substantial diagnostic burdens on hospitals.[12] Additionally, variability in pathologists' expertise and the inherent subjectivity of semi-quantitative assessments often compromise the accuracy (ACC) of disease state evaluations in patients with CG.[11,13,14] Hence, these challenges underscore a pressing clinical need for a streamlined and objective tool capable of accurately characterizing disease status in CG, thereby maximizing therapeutic benefits.

Artificial intelligence (AI) has revolutionized medicine, offering significant potential to address existing challenges. Deep learning (DL) is reshaping biomedical research, personalized medicine, and computer-aided diagnosis (CAD), potentially advancing global healthcare significantly.[15] High-capacity convolutional neural network (CNN) models have achieved notable success, surpassing human performance in some domains.[16] Particularly in computational pathology, CNNs have emerged as an effective DL technique for classifying histological images, continuously enhancing the interpretability of their outputs.[17,18]

Recent research has primarily focused on training DL models on H&E-stained whole slide images (WSIs) for precise clinical diagnosis and prognostic analysis of GC,[19–23] while comparatively less attention has been given to morphological grading and subtype classification in CG.[24–27] Steinbuss et al. utilized a CNN to classify gastritis subtypes on a small dataset (n = 135),[24] while Huang et al. applied weakly supervised learning to train a dichotomous model, achieving approximately 94% ACC in identifying atrophy, activity, and IM in CG.[25] Similarly, Lan et al. employed machine learning approaches with manually selected quantitative features to construct models that achieved an area under the receiver operating curve (AUC) of 0.901 for moderate to severe gastric atrophy.[27] Despite these advances, several challenges remain. First, most studies are constrained by small and single datasets, lacking multicenter validation, which raises concerns about overfitting and limited generalizability, conceivably leading models to focus on irrelevant features. Second, the majority of research has targeted specific CG subtypes, neglecting the more prevalent *Helicobacter pylo*ri (HP)-associated CG. Third, current evaluations frequently lack fine-grained grading of histological severity, especially for key parameters such as inflammation, activity, atrophy, and IM. Fourth, existing models heavily depend on manual pixel-level annotations and offer limited interpretability at the WSI level, hindering their ability to generate intuitive visualizations that could assist pathologists in rapidly localizing abnormal lesions. To overcome these limitations, we seek to develop clinically impactful tools that enhance diagnostic ACC, generalizability, and interpretability while integrating seamlessly into routine pathology workflows.

In this study, we complied the largest dataset of CG-related WSIs to date, annotated with fine-grained labels according to the updated Sydney system. Leveraging this high-quality dataset, we developed GastritisMIL, a pioneering DL model for precise classification of histological alterations in CG. First, GastritisMIL demonstrated superior classification performance in evaluating gastric mucosa across three multicenter external test cohorts, owing to its advanced generalization capabilities. Second, GastritisMIL exhibited greater coherence with senior pathologists and surpassed junior pathologists in several tasks. Third, GastritisMIL generated interpretable heatmaps for diverse

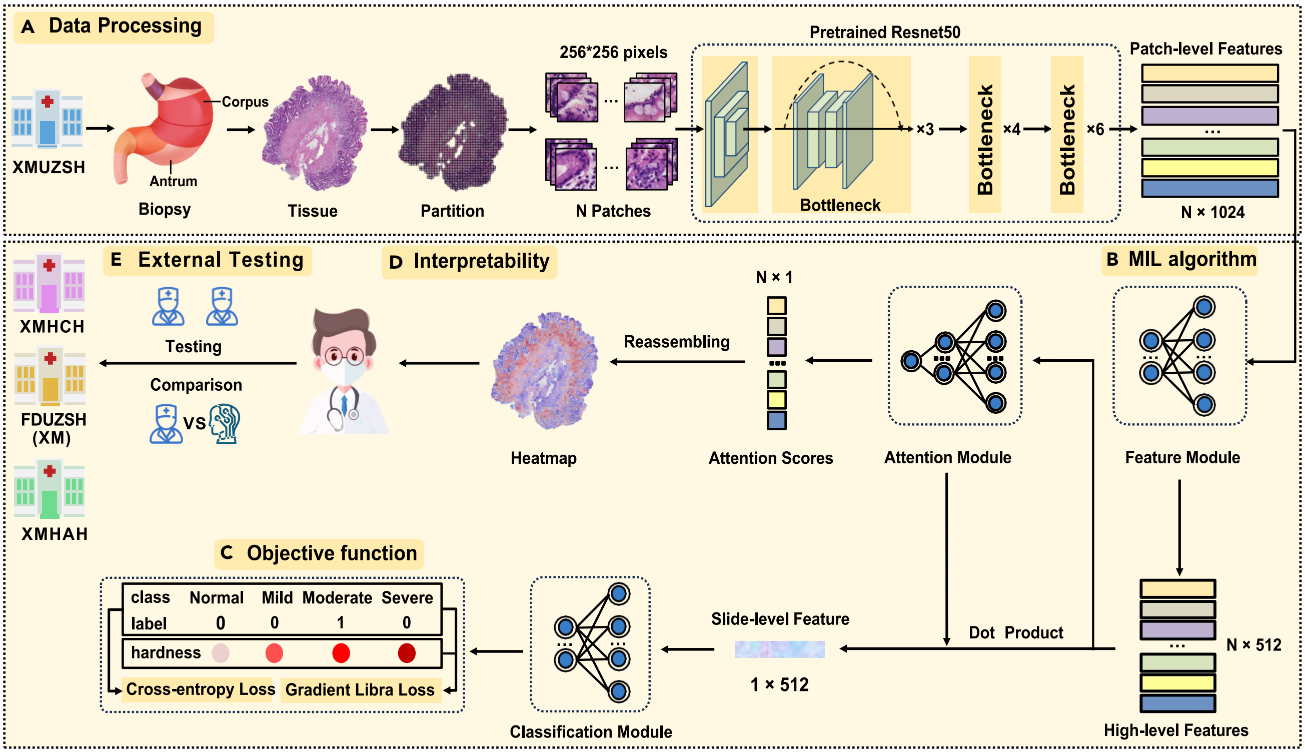

Figure 1. Overview of the algorithm—GastritisMIL

(A) Data processing and feature extracting workflow for model training from WSIs to patch-level features.
(B) The architecture of the GastritisMIL algorithm.
(C) The architecture of our designed objective function based on predicted grade.
(D) The interpretable output of WSI-level heatmaps.
(E) The external testing for GastritisMIL among three cohorts and comparison with three pathologists.
WSI, whole slide image; XMUZSH, Zhongshan Hospital of the Xiamen University; FDUZSH(XM), Zhongshan Hospital, Fudan University (Xiamen branch); XMHCH, Xiamen Haicang Hospital; XMHAH, Xiamen Humanity Hospital.

histological features, substantially mitigating the "black box" limitations of DL methods. These features enabled faster and more accurate identification of pathological alterations, effectively assisting pathologists in drafting reports and optimizing endoscopic follow-up strategies for CG patients.

RESULTS

Characteristics of patients and lesions

The overall study design is illustrated in Figures 1 and S1. Initially, a total of 2,744 patients were included from Zhongshan Hospital of Xiamen University (XMUZSH) (each patient corresponds to a single WSI). Quality control conducted by expert pathologist C (M. Lin) determined that 2,613 H&E-stained WSIs (mean age, 52.382 years ± 12.443; range, 18–88 years) from the XMUZSH cohort were suitable for use. Among them, for gastric antrum, 1,577 were suitable for the "inflammation" task (task 1), 1,746 for the "activity" task (task 2), 1,295 for the "antrum atrophy" task (task 3), and 1,743 for the "IM" task (task 4). Considering the histological similarities between gastric antrum and corpus, their cellular composition and functions differ significantly. Next, we performed model fine-tuning on an XMUZSH corpus cohort of 426 participants. The Zhongshan Hospital, Fudan University (Xiamen Branch) (FDUZSH(XM)), cohort included 117 participants assigned to tasks 1 and 2 and 78 to tasks 3 and 4. The second external cohort, Xiamen Haicang Hospital (XMHCH), comprised 170 participants in all tasks, and the third, Xiamen Humanity Hospital (XMHAH), comprised 180 participants in all tasks. The number of severely atrophic lesions was notably lower in all cohorts. Given the challenges of biopsying completely normal gastric mucosa, devoid of chronic inflammation cells and neutrophils, in real-world clinical settings, our dataset did not encompass such samples. Detailed data distributions are described in Table 1 and Figure S2.

Performance assessment for multiclass classification

Table 2 presents the performance comparison of GastritisMIL with six DL methods (CLAM-SB,[28] DSMIL,[29] DTFDMIL,[30] TransMIL[31] PatchGCN,[32] and ABMIL[33]) across four tasks, demonstrating the superior performance of GastritisMIL in most tasks and its robust classification capabilities. Specifically, GastritisMIL achieved the highest AUC in three tasks: inflammation (0.971 ± 0.003), activity (0.984 ± 0.003), and antrum atrophy (0.974 ± 0.003). Notably, in terms of AUC, ACC, and F1 score, GastritisMIL performed robustly and consistently surpassed the other frameworks for categorical effects by a significant margin across the above three tasks, showcasing its ability to accurately assess complex histological features. As shown in Table 2 and

Table 1. Baseline characteristics of included WSIs

Cohort	Sex (male)	Age (years)	Task	Normal	Mild	Moderate	Severe	Total
XMUZSH antrum cohort (*n* = 2,187)	931 (59.0%)	52.065 ± 11.962	task 1—inflammation	none	488 (30.9%)	959 (60.8%)	130 (8.2%)	1,577
	1,046 (59.9%)	52.282 ±12.513	task 2—activity	none	1,079 (61.8%)	369 (21.1%)	298 (17.1%)	1,746
	779 (60.2%)	52.252 ±12.261	task 3—antrum atrophy	481 (37.1%)	504 (38.9%)	250 (19.3%)	60 (4.6%)	1,295
	1,045 (60.0%)	52.234 ±12.463	task 4—IM	1,158 (66.4%)	253 (14.5%)	202 (11.6%)	130 (7.5%)	1,743
XMUZSH corpus cohort (*n* = 426)	211 (49.5%)	52.197 ±11.988	task 1—inflammation	none	301 (70.7%)	113 (26.5%)	12 (2.8%)	426
			task 2—activity	none	368 (86.4%)	45 (10.6%)	13 (3.1%)	426
			task 3—corpus atrophy	337 (79.1%)	38 (8.9%)	31 (7.3%)	20 (4.7%)	426
			task 4—IM	381 (89.4%)	20 (4.7%)	10 (2.3%)	15 (3.5%)	426
FDUZSH(XM) cohort (*n* = 117)	68 (58.1%)	55.784 ±10.679	task 1—inflammation	none	53 (45.3%)	59 (50.4%)	5 (4.3%)	117
			task 2—activity	none	92 (78.6%)	17 (14.5%)	8 (6.8%)	117
			task 3—antrum atrophy	12 (15.4%)	25 (32.1%)	29 (37.2%)	12 (15.4%)	78
			task 4—IM	30 (38.5%)	14 (17.9%)	18 (23.1%)	16 (20.5%)	78
XMHCH cohort (*n* = 170)	91 (53.5%)	51.876 ±12.167	task 1—inflammation	none	50 (29.4%)	92 (54.1%)	28 (16.5%)	170
			task 2—activity	none	88 (51.8%)	35 (20.6%)	47 (27.6%)	170
			task 3—antrum atrophy	59 (34.7%)	56 (32.9%)	43 (25.3%)	12 (7.1%)	170
			task 4—IM	113 (66.5%)	38 (22.4%)	10 (5.9%)	9 (5.3%)	170
XMHAH cohort (*n* = 180)	111 (61.7%)	50.810 ±13.554	task 1—inflammation	none	96 (53.3%)	68 (37.8%)	16 (8.9%)	180
			task 2—activity	none	90 (50.0%)	34 (18.9%)	56 (31.1%)	180
			task 3—antrum atrophy	26 (14.4%)	43 (23.9%)	39 (21.7%)	72 (40.0%)	180
			task 4—IM	89 (49.4%)	18 (10.0%)	44 (24.4%)	29 (16.1%)	180

XMUZSH, Zhongshan Hospital of the Xiamen University; FDUZSH(XM), Zhongshan Hospital, Fudan University (Xiamen branch); XMHCH, Xiamen Haicang Hospital; XMHAH, Xiamen Humanity Hospital; IM, intestinal metaplasia.

Table 2. Diagnostic performance of GastritisMIL in XMUZSH, including the antrum cohort utilizing ResNet50 (internal test cohort)

	Task 1—inflammation			Task 2—activity		
Method	AUC	ACC	F1 score	AUC	ACC	F1 score
CLAM-SB[28]	0.957 ± 0.002	0.868 ± 0.007	0.766 ± 0.010	0.967 ± 0.006	0.892 ± 0.009	0.852 ± 0.016
DSMIL[29]	0.905 ± 0.008	0.818 ± 0.002	0.600 ± 0.009	0.956 ± 0.004	0.860 ± 0.008	0.796 ± 0.010
DTFDMIL[30]	0.954 ± 0.005	0.860 ± 0.008	0.710 ± 0.039	0.981 ± 0.003	*0.916 ± 0.009*	*0.886 ± 0.011*
TransMIL[31]	0.916 ± 0.012	0.810 ± 0.009	0.668 ± 0.030	0.939 ± 0.016	0.840 ± 0.023	0.776 ± 0.040
PatchGCN[32]	0.953 ± 0.003	0.858 ± 0.007	0.770 ± 0.007	0.978 ± 0.003	0.904 ± 0.009	0.868 ± 0.014
ABMIL[33]	*0.969 ± 0.003*	*0.879 ± 0.006*	*0.812 ± 0.012*	*0.982 ± 0.003*	0.910 ± 0.007	0.876 ± 0.010
GastritisMIL (ours)	**0.971 ± 0.003**	**0.892 ± 0.006**	**0.832 ± 0.009**	**0.984 ± 0.003**	**0.922 ± 0.006**	**0.900 ± 0.010**
Method	Task 3—antrum atrophy			Task 4—intestinal metaplasia		
	AUC	ACC	F1 score	AUC	ACC	F1 score
CLAM-SB[28]	0.896 ± 0.009	0.694 ± 0.019	0.524 ± 0.014	0.909 ± 0.005	0.788 ± 0.007	0.602 ± 0.002
DSMIL[29]	0.868 ± 0.009	0.658 ± 0.016	0.486 ± 0.014	0.866 ± 0.009	0.748 ± 0.008	0.526 ± 0.018
DTFDMIL[30]	0.961 ± 0.005	0.810 ± 0.012	0.616 ± 0.009	0.939 ± 0.011	0.818 ± 0.012	0.630 ± 0.037
TransMIL[31]	0.894 ± 0.016	0.696 ± 0.029	0.632 ± 0.046	0.872 ± 0.035	0.778 ± 0.034	0.604 ± 0.066
PatchGCN[32]	0.952 ± 0.004	0.800 ± 0.005	0.776 ± 0.015	*0.973 ± 0.003*	**0.892 ± 0.008**	**0.828 ± 0.012**
ABMIL[33]	*0.970 ± 0.002*	*0.847 ± 0.011*	*0.814 ± 0.021*	0.972 ± 0.002	0.886 ± 0.005	0.802 ± 0.013
GastritisMIL (ours)	**0.974 ± 0.003**	**0.866 ± 0.009**	**0.846 ± 0.013**	**0.975 ± 0.003**	*0.887 ± 0.005*	*0.818 ± 0.008*

Data are metrics as mean ± SD. The best values are highlighted in bold face. The second-best values are in italic. AUC, area under the receiver-operating characteristic curve; ACC, accuracy.

Figure S3, GastritisMIL exhibited greater ACC than the other six methods in detecting atrophy changes, a critical indicator for assessing the high risk of GC development. Although PatchGCN slightly outperformed GastritisMIL in task 4 (IM), with a marginally higher ACC (0.892 ± 0.008 vs. 0.887 ± 0.005) and F1 score (0.828 ± 0.012 vs. 0.818 ± 0.008), GastritisMIL still demonstrated competitive performance, ranking second in both metrics while matching PatchGCN in ACC (0.887 ± 0.005). Moreover, its average clinical utility index (CUI) consistently exceeded 0.640 (Table S1), especially in task 2, in which it achieved "excellent utility" (CUI+, 0.812 ± 0.005; CUI−, 0.860 ± 0.012). These results underscore the effectiveness of GastritisMIL in classifying histological features associated with inflammation, activity, atrophy, and IM in CG patients. While PatchGCN excelled slightly in a single task, the overall balanced and superior performance of GastritisMIL across multiple tasks demonstrated its potential as a state-of-the-art solution for pathology image analysis.

On the XMUZSH corpus cohort (fine-tuning cohort), GastritisMIL achieved an ACC exceeding 90% across all tasks, with a particularly notable performance of 96% ACC in detecting glandular IM (Table 2). In identifying the extent of neutrophil infiltration (task 2), GastritisMIL maintained a balanced AUC (0.984 ± 0.003 and 0.977 ± 0.008) and CUI (CUI+/CUI−: 0.812 ± 0.017/0.860 ± 0.012 and 0.794 ± 0.047/0.826 ± 0.035) on the internal antrum and corpus cohort. However, its discriminatory ability between mild and moderate levels for corpus was relatively weaker. A comprehensive analysis of confusion matrices for antrum and corpus across the four tasks revealed the percentage of correct predictions (darker cell color indicates higher prediction ACC) (Figure 2). All receiver operating curves (ROCs) from 5-fold cross-validation results are displayed in Figures 2E–2H and 2M–2P. Notably, our model evidently reduced the likelihood that junior pathologists would overlook subtle hard-to-detect cellular and tissue transformations, such as unusual cell infiltration and glandular variation, as corroborated by follow-up comparisons with three pathologists with varying experience levels.

Selection of the feature exaction backbone of GastritisMIL

On the internal test cohort, Macro-AUC of the GastritisMIL framework (Figures 1 and S4) surpassed that of the other six models in both the ternary (tasks 1 and 2) and the quaternary (tasks 3 and 4) classifications on multiple different feature extraction backbones at 40× magnification (Figure 1; Tables 2, S2, and S3). A deep residual neural network (ResNet50[34]), along with another three ImageNet-based architectures (DenseNet121,[35] EfficientNet,[36] and Inception-ResNetV2[37]) and five pathologic foundation models (PLIP,[38] UNI,[39] CONCH,[40] GigaPath,[41] and TITAN[42]), was employed to extract histological features of WSIs. These methods were then used to compare the performance in classifying the severity of abstract changes. ResNet50 outperformed all other backbones in task 1, task 2, and task 3, but slightly lagged behind GigaPath in task 4, which achieved the best results, specifically in identifying IM severity. Across four downstream tasks, the internal test set performance of several mainstream methods essentially outperformed that of the pretrained pathological foundation models when utilizing features extracted by ResNet50. Compared with GigaPath and PLIP, ResNet50 demonstrated more robust performance, particularly excelling in assessing glandular atrophy (task 3) in CG (ACC: 0.866 ± 0.009 vs. 0.822 ± 0.011 vs. 0.814 ± 0.010) (Table S2).

Furthermore, we conducted a more rigorous and comprehensive performance comparison across multiple magnifications to evaluate each backbone under its optimal magnification performance levels (Figure S4). It is noteworthy that all backbone architectures achieved their peak classification performance when evaluated at 40× magnification. Moreover, ResNet50

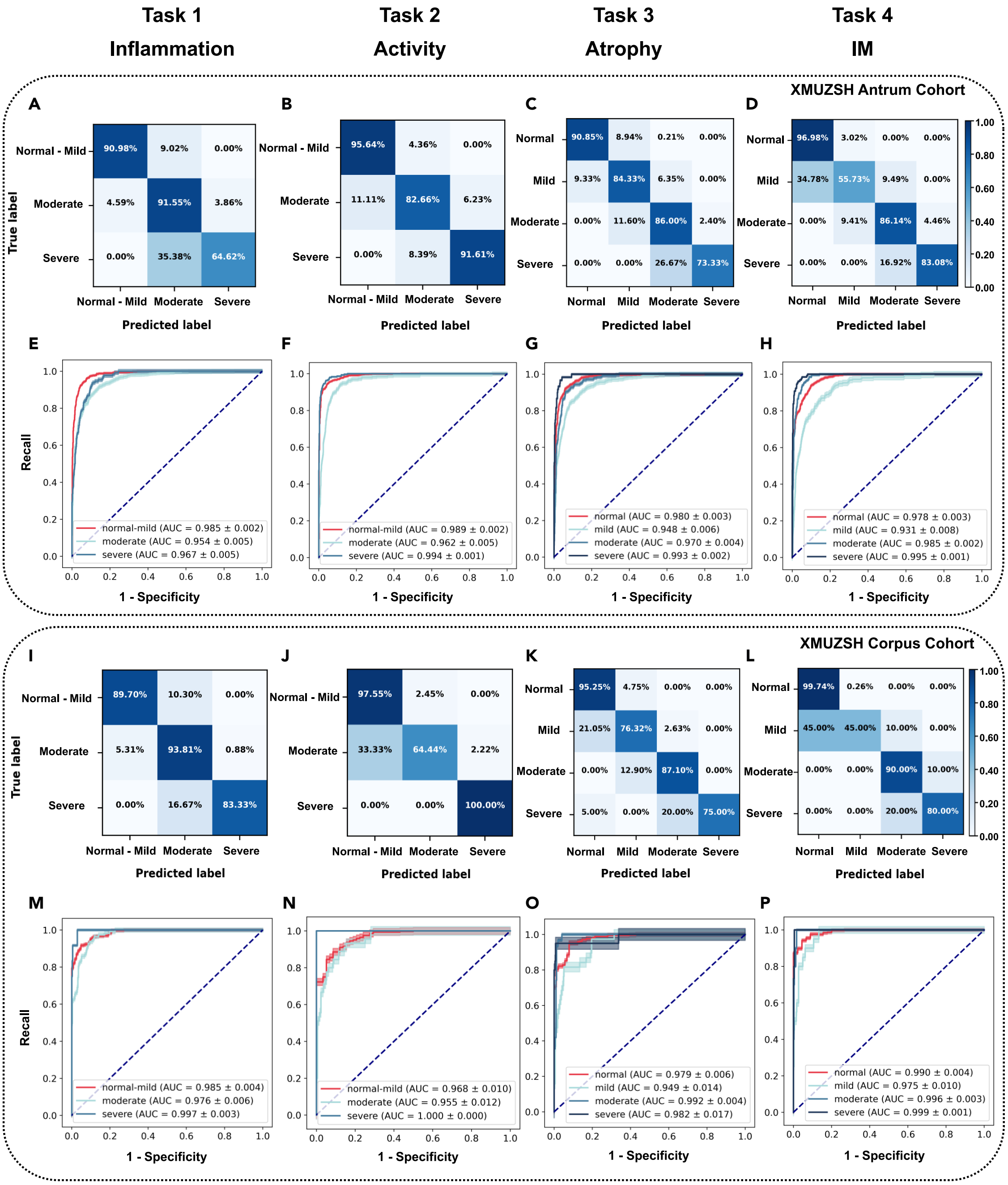

Figure 2. Diagnostic ability of GastritisMIL for the internal test cohort including antrum and corpus (the mean result of 5-fold cross-validation)
(A–D and I–L) Confusion matrices of ternary (tasks 1 and 2) and quaternary (tasks 3 and 4) classification in the internal test cohort (A–D for antrum, I–L for corpus).
(E–H and M–P) ROC curves of GastritisMIL in evaluating various alterations of the above four tasks (E–H for antrum, M–P for corpus).

demonstrated the most balanced and outstanding overall classification performance at 40× magnification. Consequently, we selected 1,024-dimensional feature representations generated from ResNet50 for subsequent training.

Selection of the proper magnification of WSIs

Our experiments across four tasks demonstrated that sampling at 40× magnification resulted in the best performance for the model. As shown in Figure S4 and Table S4, in task 1 (inflammation), the AUC at 40× (0.971 ± 0.003) was significantly higher than at 20× (0.877 ± 0.006) and 10× (0.661 ± 0.022), while the F1 score at 40× (0.832 ± 0.009) also outperformed the other magnification levels. Similarly, comparable results were observed in the other three tasks, where 40× magnification also outperformed 20× and 10× in terms of AUC, ACC, and F1 score. These results suggest that higher magnification levels,

CellPress
OPEN ACCESS

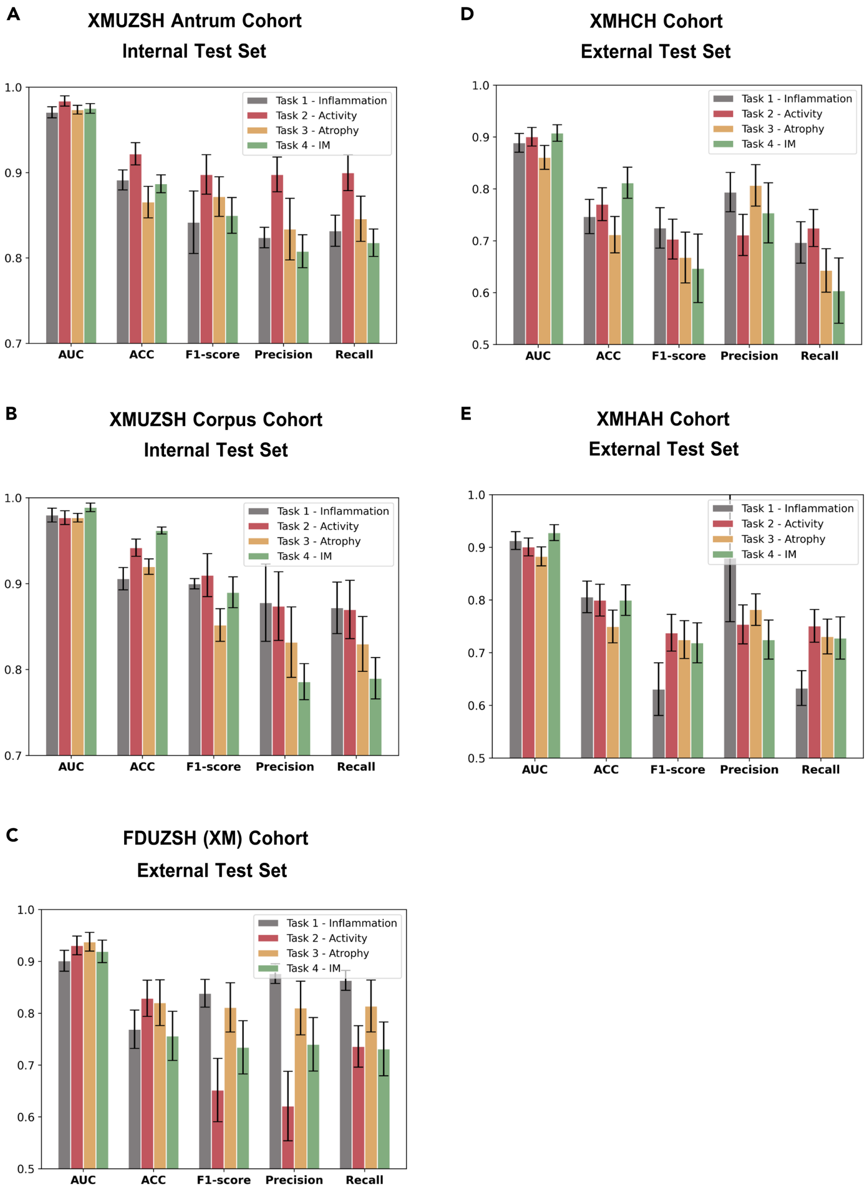

Figure 3. Bar plots displaying AUC, ACC, F1 score, precision, and recall of GastritisMIL among internal and three external cohorts across four tasks, including inflammation (task 1), activity (task 2), atrophy (task 3), and IM (task 4)

(A) XMUZSH antrum cohort (internal test cohort 1).
(B) XMUZSH corpus cohort (internal test cohort 2).
(C) FDUZSH(XM) cohort (external test cohort 1).
(D) XMHCH cohort (external test cohort 2).
(E) XMHAH cohort (external test cohort 3).

particularly 40×, provide more detailed semantic information, which is critical for accurate feature extraction and improved model performance across a range of tasks.

Independent external test cohorts of GastritisMIL

To validate the generalization of GastritisMIL, approximately 467 WSIs from three external test cohorts were utilized to assess its diagnostic performance at the WSI level. Table S5 shows the AUC, ACC, F1 score, precision, recall, and negative predictive value (NPV) of GastritisMIL in the three external cohorts. In task 1, our model achieved an average precision exceeding 0.936, which is better than the performance in other tasks within the same three cohorts. However, mild neutrophilic infiltration was occasionally misidentified as moderate. In addition, the AUC of GastritisMIL in task 2 generally exceeded 0.900, underscoring its robustness and superiority across diverse data cohorts. Furthermore, in tasks 3 and 4, which are critical for reliably evaluating the risk of early-stage GC, our model attained the highest AUC (0.937 ± 0.018) on the FDUZSH(XM) cohort for atrophy and for IM (0.952 ± 0.017). Similarly, our model intermittently misdiagnosed subtle moderate atrophic changes, which are challenging to discern, as either mild or severe atrophic lesions, reflecting the complexities of gastritis diagnosis. In addition to these metrics, on the FDUZSH(XM) cohort, the average NPVs for tasks 1 and 2 were close to unity, illustrating the proficient predictive ACC for negative class samples in the ternary classification framework. The results of the internal and external test cohorts are presented as bar plots in Figure 3.

Classification performance of proposed model vs. pathologists

By overlaying the model-generated attention scores for each patch onto the original WSIs, a visual heatmap was created, aiding pathologists with varying expertise in diagnosing gastritis-related pathologies (Figure 4). Tables S5–S8 present the diagnostic performance of our model across the three external cohorts (FDUZSH(XM), XMHCH, and XMHAH), each assessed by three pathologists (5, 10, and 12 years experience, respectively) with different degrees of diagnostic experience. In our analysis of multiclass classification tasks, the model consistently demonstrated superior ACC over junior pathologist A in most assessments, aligning closely with the diagnostic performances of the two senior pathologists.

In predicting the severity of inflammatory lesions (task 1), GastritisMIL outperformed all three pathologists across 180 WSIs on the XMHAH cohort, especially senior pathologist C (0.806 ± 0.030 vs. 0.756 ± 0.031; p = 0.262, McNemar's test) (Table S8). For task 2, aimed at predicting the extent of neutrophil infiltration, the model essentially outperformed pathologist A (0.829 vs. 0.803, p = 0.678, McNemar's test; 0.824 vs. 0.641, $p < 0.001$, McNemar's test; 0.800 vs. 0.650, p = 0.003, McNemar's test) on WSIs, but not the two senior pathologists (0.829 vs. 0.906 and 0.932, 0.824 vs. 0.829 and

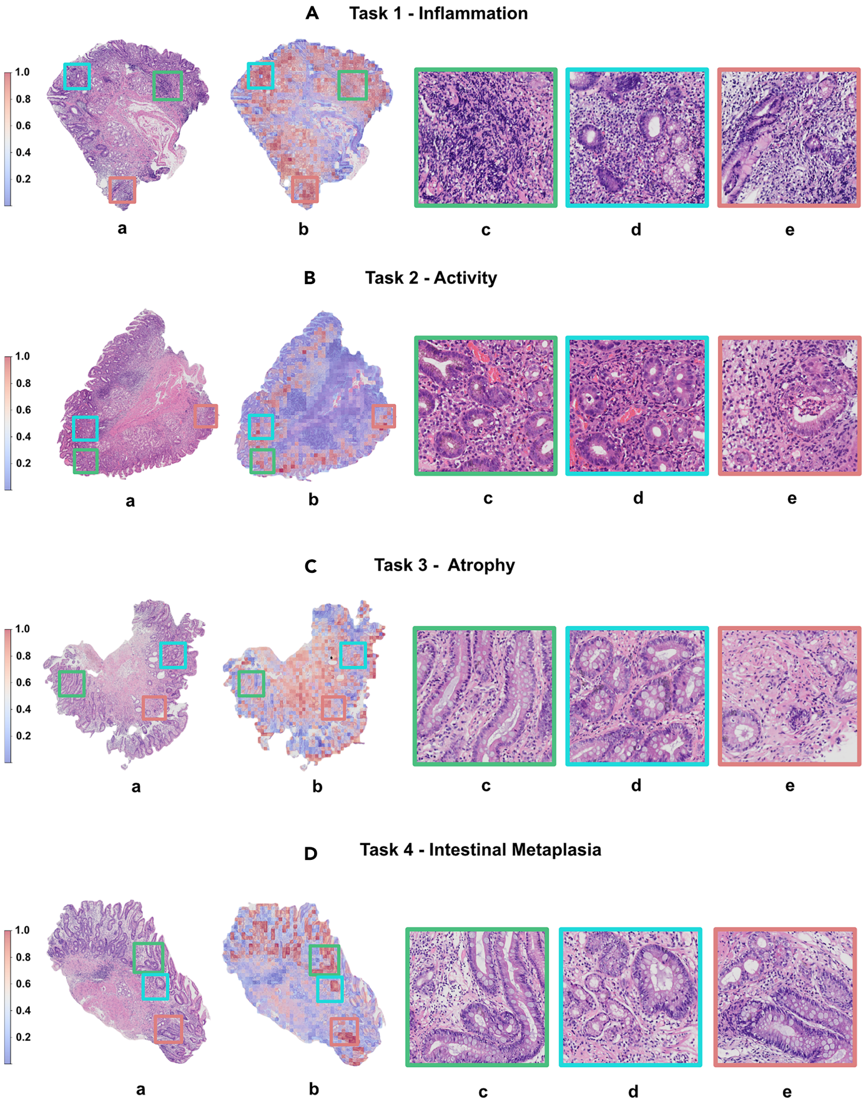

Figure 4. Visualization heatmaps of the internal test cohort (XMUZSH) through mapping on the model-generated attention scores for each patch back onto original WSIs

The transition from pale blue to dark red denotes heightened model attention toward the severity of the lesion. (A)–(D) depict the original WSIs and interpretable heatmaps of the GastritisMIL's performance across four tasks according to the updated Sydney system. (c)–(e) show representative districts with evident alterations from the original WSI.

0.853, and 0.800 vs. 0.861 and 0.872, respectively) (Figure 3; Tables S6–S8). In addition, in the multiclass classification of antrum atrophy, a subjective indicator in clinical settings, our model significantly surpassed junior pathologist A on both the FDUZSH(XM) and the XMHAH cohorts (0.821 vs. 0.641, p = 0.038, McNemar's test, and 0.750 vs. 0.611, p = 0.011, McNemar's test, respectively), exhibiting approximately 15% higher diagnostic ACC (Tables S6 and S8). Furthermore, in evaluating IM, although our model's average ACC of approximately 80.0% did not exceed that of the three pathologists, it did have higher weighted kappa over 0.850 on the FDUZSH(XM) and XMHAH cohort (Tables S6 and S8), demonstrating nearly identical performance compared to the two gastrointestinal pathology specialists.

Radar charts intuitively describe the comparison between our model and the three pathologists across the three external test cohorts (Figure 5). And the specific label distribution of GastritisMIL and the three pathologists on three external test cohorts is displayed in Figure 6. Furthermore, we implemented age-stratified performance analysis across internal and three external cohorts (Tables S10 and S12).

Comparison of judgment time between proposed model and pathologists

We randomly selected 10 WSIs from three external test cohorts (not involved in model training) to create a small cohort for comparing the judgment time of GastritisMIL with that of three pathologists (A, B, and C) under the same clinical scenario. To ensure fairness, they were provided with the same set of 10 WSIs on laptops, rather than examining glass slides through a microscope, thus maintaining consistency across the evaluation environment. The model's pipeline comprised three stages: dividing patches (44.14 s), extracting features (224.15 s), and inferencing WSI-level labels. The inference process for the four downstream tasks was conducted concurrently, with each task running on a separate NVIDIA GTX 3090 GPU, using an Intel Xeon Silver 4210R CPU @ 2.40 GHz. The specific inference times required for each task were as follows: 0.792 s for inflammation (task 1), 0.823 s for activity (task 2), 1.161 s for atrophy (task 3), and 0.748 s for IM (task 4). Moreover, we assessed the computational complexity of GastritisMIL by utilizing the floating point operations (FLOPs) metric, quantifying the number of FLOPs required for each inference. Task 1 and task 2 required 849,754,880 FLOPs, while task 3 and task 4 required 849,755,136 FLOPs. Despite its computational complexity, the inference process is suitable for standard GPUs and imposes minimal resource requirements.

As shown in Figure S5, our model required less than 5 min to complete tasks equivalent to the workload of a senior pathologist of 15 min (4.5 min vs. 14.80 min and 14.58 min), achieving an efficiency over six times that of a junior pathologist (4.5 min vs. 27.67 min). These results underscore the potential value of GastritisMIL in assisting pathologists with the rapid identification of histological alterations in CG, demonstrating its capacity to

Please cite this article in press as: Xia et al., GastritisMIL: An interpretable deep learning model for the comprehensive histological assessment of chronic gastritis, Patterns (2025), https://doi.org/10.1016/j.patter.2025.101286

CellPress
OPEN ACCESS

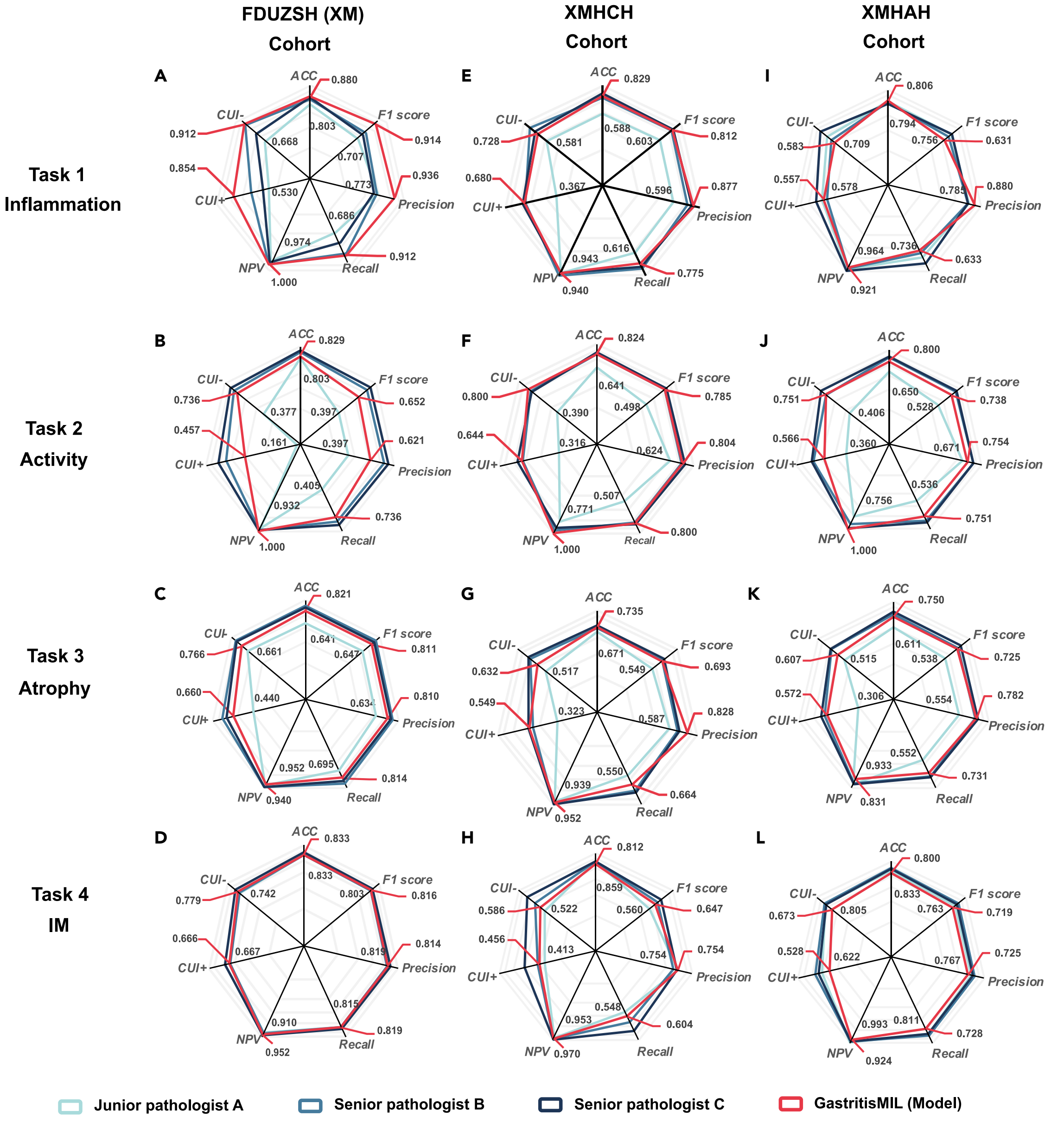

Figure 5. Radar plots of three external test cohorts comparing the performance of the model with that of three human pathologists

Each plot displays results from one external cohort, with separate lines representing the model and each pathologist. ACC, accuracy; NPV, negative predictive value; CUI+, positive clinical utility index; CUI−, negative clinical utility index.

substantially alleviate the clinical workload and enhance diagnostic efficiency. Generally speaking, this comprehensive time analysis ensures a more accurate and realistic comparison of the model's efficiency relative to pathologists during digital pathology workflow.

It is conceivable that integrating the model into the pathology department's slide viewing system will enable pathologists to rapidly obtain multitask evaluation of batched WSIs along with severity-based visualization heatmaps, requiring only secondary review of the inference results to effectively reduce workload and enable accurate localization of abnormalities.

Comparison of GastritisMIL performance with recent CG-AI research

Due to the lack of publicly available code, related datasets, and detailed workflows, we could compare computational results of GastritisMIL with their reported outcomes from only three recent representative studies (LR,[27] GasMIL,[43] and AMMNet[44]) in four tasks (as shown in Table S9). In comparison, GastritisMIL consistently outperformed them across four tasks, particularly in inflammation (AUC = 0.971), activity (AUC = 0.984), antrum atrophy (AUC = 0.984), and IM (AUC = 0.975), achieving the highest AUC, recall, and specificity. Moreover, GastritisMIL excelled over GasMIL in the semi-quantitative assessment of atrophy

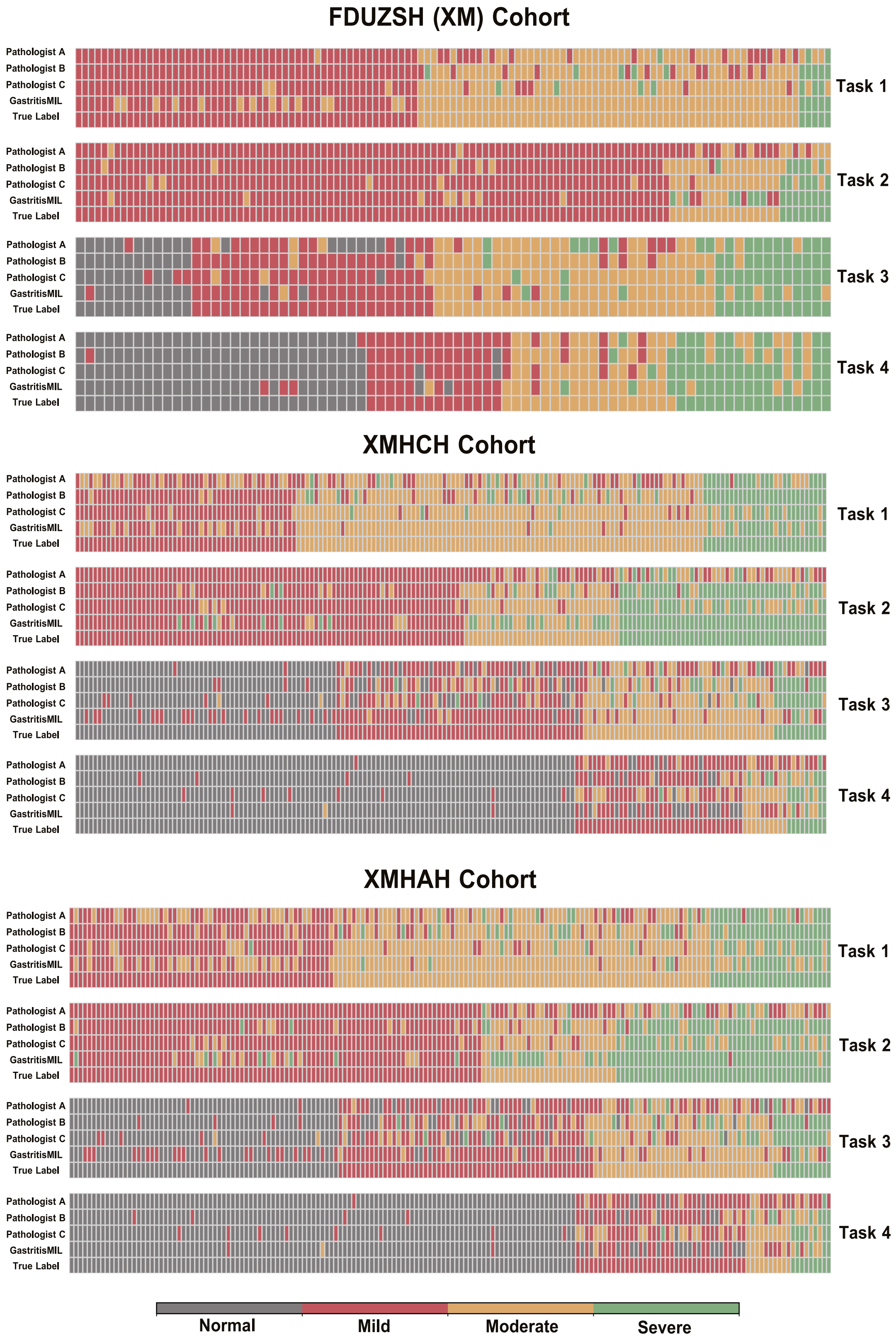

Figure 6. The specific label distribution of GastritisMIL and three pathologists on three external test cohorts

and IM, highlighting its potential value in evaluating glandular loss and newly formed intestinal glands.

DISCUSSION

Continual follow-up evaluation of histological alterations within a proper interval is essential for the timely detection and treatment of early-stage GC.[4,5,14] With the increasing discrepancy between endoscopic observations and microscopic evaluations of gastric biopsy specimens, particularly in the era of digital pathology, a streamlined and objective tool with excellent ACC and recall is needed.

To the best of our knowledge, GastritisMIL is the most comprehensive assessment tool for CG, including gastric antrum and corpus (Figure 1), utilizing the largest multicenter datasets of CG WSIs to precisely categorize various histological lesions, including inflammation, activity, atrophy, and IM. We compared its performance to that of three human pathologists to validate its generalization and robustness. Our results demonstrated that the GastritisMIL model outperformed the junior pathologist in most tasks. Pathological images of CG contain abundant semantic information, which the model can discern through a feature vector generated by the pretrained ResNet50 network.[34] Consequently, high-quality visualization heatmaps can be created to assist pathologists in rapidly locating current microscopic alterations. Therefore, GastritisMIL offers a valuable tool for gastrointestinal pathologists and can significantly alleviate diagnostic burdens, enhance slide review efficiency, and minimize the risk of overlooking rare lesions, thereby alerting the associated risks and enhancing endoscopists' clinical decision-making process for individually optimizing endoscopic follow-up intervals for CG patients.

In recent CG pathology-related research, DL methods have garnered attention. Although most studies have developed relatively advanced models for diagnosing CG, their generalizability among other centers still needs further confirmation due to some limitations, such as the absence of multicenter validation and a thorough grading method, as well as dependence on manual delineations. Recently, a study from Wangjing Hospital established a classification model for atrophy and IM based on the updated Sydney system to assess the GC risk from WSIs.[43] Compared to the classification of inflammation and activity, their model exhibited a notable decrease in terms of AUC for the above two tasks (0.877 [95% CI 0.855–0.897] and 0.884 [95% CI 0.862–0.902), highlighting challenges in achieving optimal predictive efficiency and revealing issues with relatively weaker patch-level interpretability.

Our study addressed the aforementioned limitations and offers notable advances. Notably, we trained GastritisMIL, a weakly supervised DL framework, eliminating the need for manual delineation of lesion areas by pathologists. Instead, our model uses slide-level digital labels derived from infiltration ratios. We capitalized on high-quality data from labels meticulously scrutinized by two senior pathologists, significantly reducing the manual workload. Moreover, our model achieved an average AUC exceeding 0.970 across all four tasks, a mean recall surpassing 0.800, and an average CUI exceeding 0.700, surpassing the good utility threshold of 0.640. These results show that the model outperformed most models published to date. Moreover, in this multicenter study, our model demonstrated robust generalizability in three external cohorts on 467 real-world digital slides from three tertiary hospitals instead of deriving from public datasets. The model consistently achieved an AUC above 0.900, its results aligned closely with the assessments of two senior pathologists (quadratic weighted kappa coefficient showed good concordance), and it outperformed the less experienced pathologist in most tasks. Finally, our model accurately identified potential areas of interest in the 40× field of view and generated high-quality, slide-level visualization heatmaps (Figure 4). The red gradations in these heatmaps represent the model's focus on the severity of suspicious lesions. Unlike previous studies that focused on visualizing select top-k high-probability patches, our approach delivers interpretable results across all WSIs, capturing both global histological alterations and detailed features of all foreground patches. This offers an objective tool to facilitate pathologists in drafting pathological reports within a short time.

After visualizing all slides from internal and external cohort, two pathologists (M. Lin and Y.Y.) meticulously reviewed these WSIs. In task 1 (Figure 4A), we observed that our model accurately identified regions that are abundant in chronic inflammatory cells, such as lymphocytes and plasma cells, and delineated these areas clearly from the surrounding normal tissue within the entire slide. However, in 39.8% of failure cases (68 out of 171) from task 1, the nuclei of glandular epithelial cells within the intrinsic glands were prone to misidentification as chronic inflammatory cells due to compression caused by cellular mucus present in the glands. In task 2 (Figure 4B), some regions with segmented neutrophils were also precisely localized. In tasks 3 and 4 (Figures 4C and 4D), the heatmaps successfully delineated the stratified boundaries between metaplastic intrinsic glands and normal glands. Additionally, we discerned that epithelial cells in some gastric pits were mistakenly identified as exhibiting atrophic changes, likely due to structural similarities between the pit epithelium and the intrinsic glands, which may lead to confusion in interpretation. The quantitative analysis revealed that 48.3% (84 out of 174) of failure cases in task 3 and 54.3% (107 out of 197) of the failure cases in task 4 were associated with difficulties in differentiating between pit and glandular epithelium. The chi-squared test result demonstrated that there was no significant difference in the occurrence of this error between tasks 3 and 4 ($p = 0.290$). Further analysis of the 107 structural confusion errors in task 4 revealed that 74.8% (80 out of 107) involved misclassifications between mild IM and normal slides, while 44.1% (37 out of 84) of the errors in task 3 were due to confusion between mild atrophy and normal slides. These findings suggest that normal and mild IM are more susceptible to structural confusion errors than normal and mild atrophy, likely due to an imbalance in the training cohort, where non-IM slides comprise approximately five times the proportion of mild IM slides (66.4% vs. 14.4%).

In addition, we similarly conducted a comprehensive analysis of all failure cases from three external test cohorts across three downstream tasks (Table S14). Our findings indicate that there was no statistically significant difference in the error proportions for the aforementioned classification errors between the external cohorts and internal cohorts in each specific task (as determined by the chi-squared test). This suggests that, while the observed

error patterns exist across different cohorts, their impact is not statistically significant. Overall, our visualization results effectively highlight suspicious lesion areas and provide rapid diagnostic results to pathologists, significantly reducing their workload and the risk of missed diagnoses. Once our model is implemented, pathologists can streamline their workflow by simply reviewing and validating its output results, greatly facilitating its integration into clinical practice.

Significant variations in H&E staining protocols across hospitals could introduce irrelevant variables that negatively impact the model's decision-making performance and generalizability. In response to this concern, we explored staining normalization techniques,[45–47] specifically utilizing the Macenko[46] method to normalize all WSIs in our dataset based on a target image derived from the XMUZSH cohort. However, our results indicated a general deterioration in the model's performance across the three external test cohorts (Table S11). A retrospective analysis indicated that the current normalization methods, although beneficial, function primarily at the patch level, which may fail to fully capture the comprehensive range of color variations present at the WSI level. This limitation can result in inconsistencies in staining representation and the emergence of discernible tiling artifacts (Figure S6). Additionally, some scaled images exhibited significant noise, further indicating a lack of fidelity in the normalization process. These findings suggest that patch-based normalization may result in a "distorted" staining style, contributing to the loss of critical histological information and diminishing the model's generalizability.

Furthermore, we identified data distribution shifts between the external test sets and the training set as a key factor contributing to performance degradation. An analysis of age and label distributions revealed that the internal test set closely resembles the training set, yielding the highest ACC (Tables S12 and S13). In contrast, external test sets exhibited varying degrees of distributional differences, particularly in IM (task 4), where data labels were concentrated in categories with limited samples ("moderate" and "severe"). Given the relatively small overall sample size in the external test set, even minor classification errors can lead to significant fluctuations in performance. This imbalance, compounded by the subjective nature of evaluating IM and atrophy, presents additional challenges for prediction. Despite these hurdles, our model consistently outperformed prior studies, such as GasMIL,[43] in terms of AUC and recall across the majority of external test sets in tasks 3 and 4. Collectively, these findings underscore the need for advanced normalization strategies and the importance of employing larger, more balanced external datasets to enhance model generalizability and reliability in clinical settings.

Our study also has other limitations. First, our model directly outputs the severity of histological alterations based on WSIs, rather than the risk of early-stage GC development following OLGA/OLGIM staging system.[4,5] Although this approach achieved more accurate assessment performance to a certain extent, future work should aim to optimize GastritisMIL to enable direct stratification of GC risk, providing greater clinical utility in clinical workflows. Second, we may not incorporate relevant diagnostic information from endoscopic images, potentially overlooking significant macroscopic changes. Future efforts will focus on integrating features from pathological and endoscopic images to develop endoscopy-pathology foundation models, pretrained on large real-world multimodal CG datasets.[48] Third, while our results indicated that our model performs comparable to expert pathologists in statistical terms, slight ACC gaps persist in certain tasks, likely due to challenges in identifying subtle histological variations in tasks 3 and 4. Future work will focus on integrating multiscale features and introducing an adaptive attention mechanism to better capture critical histopathological features of atrophy and IM across various magnifications (e.g., 10×, 20×, and 40×). Finally, although we have demonstrated outstanding performance across four cohorts, the generalizability and applicability of GastritisMIL still require further validation through larger prospective clinical cohorts on a national scale.

In conclusion, GastritisMIL harnesses the power of DL to create an objective classification tool for inflammation, activity, atrophy, and IM, displaying excellent performance compared to all existing models in CG pathology. Rigorous validation on external cohorts demonstrated a diagnostic ACC comparable to that of pathologists with over a decade of experience and a significant value in assisting assessment. This promising development significantly reduces the workload of pathologists and provides a concise assessment of CG, thereby enhancing guidance for stratifying the risk of early-stage GC in the future clinical practice.

METHODS

Method details

Patient population

To enhance generalizability, four cohorts were retrospectively collected from the following institutions: XMUZSH, FDUZSH (XM), XMHCH, and XMHAH. A total of 2,187 XMUZSH slides, each containing a patient sample, were analyzed, including 800 WSIs that will soon be publicly available on the ScienceDB platform.[49] Patients diagnosed with CG underwent standard upper endoscopy (EC450, EC580, EC600, and CF-EC760, Fuji Medical Systems) targeting the gastric antrum. Images from eligible XMUZSH patients, collected between March 2020 and August 2023, were randomly assigned to the train, validation, and internal test cohorts in a 6:2:2 ratio. Samples from the three other tertiary hospitals served as external test cohorts to validate the generalizability of GastritisMIL.

Patient inclusion criteria were as follows: (1) age 18 or older; (2) definitive diagnosis confirmed via endoscopic examination, regardless of HP infection status; and (3) availability of clear, undamaged pathological slides with sufficient clinical data, free from loss, deterioration, or mold.

Concurrently, exclusion criteria for pathology slides included: (1) histological findings of intraepithelial neoplasia or previous upper gastrointestinal surgery; (2) incomplete biopsy sampling of the lamina muscularis mucosae or WSIs indicating "chronic superficial mucosa inflammation, transitional zone mucosa, and corpora mucosa; (3) CG patients simultaneously diagnosed with gastric ulcers, polyps, or neoplasia via gastroduodenoscopy; and (4) intermediate to advanced gastric carcinoma diagnosed prior to gastroscopy.

Sample collection

At least two targeted mucosal specimens were collected from the most representative areas of inflammation or suspected early intraepithelial neoplasia of the gastric antrum and corpus, corresponding to the endoscopically evaluated sites. Each pathological specimen was stored in a separate bottle according to the biopsy site, as required by the updated Sydney system. Subsequently, samples from identical sections were collectively processed for a single biopsy. Following formalin fixation, paraffin embedding, and H&E staining, the biopsies were digitally scanned at 40× magnification (0.086 μm per pixel) using a ZEISS Axio Scan 7 digital slide scanner (Carl Zeiss Microscopy GmbH, Jena, Germany). Prior to the review of these slides, all personal information regarding the patients had been anonymized through a rigorous de-identification process. This includes removing identifiable details such as names, medical record numbers, and other sensitive data to ensure WSIs and labels cannot be traced back to individual patients. Two senior pathologist (M. Lin and R.T.) verified the quality of all converted WSIs, and the high-quality slides were used to train and test GastritisMIL, achieving excellent ACC in evaluating histological changes (Figure S1).

To minimize the influence of subjective factors in image annotation, typical pathological slides and corresponding labels were presented before review. Two expert pathologists (M. Lin and R. T.), each with over 10 years of experience, meticulously reviewed the labels from XMUZSH. Additionally, two expert pathologists, B (Y.Y.) and C (M. Lin), with over 10 years of experience, along with a junior pathologist A (F.L.), with approximately 5 years of experience, marked the WSIs from three external test cohorts. Labels were finalized when two or more pathologists agreed; otherwise, expert C's opinion was adopted following group discussion. Their evaluations focused on inflammation, discerned through monocytes, lymphocytes, and plasma cells in the mucosal layer, and "activity," marked by neutrophil infiltration. These assessments were classified as normal to mild, moderate, and severe. Additionally, they assessed the degree of atrophy and IM, labeled as normal, mild, moderate, or severe (Figure S2).

Construction of classification model—GastritisMIL

Due to the large size of WSIs, employing them as direct inputs for traditional CNNs poses challenges during training. Multiple instance learning (MIL) addresses this by grouping samples into bags with weak slide-level labels, allowing for analysis without detailed annotations. This makes MIL particularly suitable for pathological image analysis in clinical settings. The structure of GastritisMIL, based on MIL, is detailed in Figure 1.

Given the high resolution of pathological images, data preprocessing is essential to reduce computational and interference load. We used image-processing techniques to remove irrelevant background information and isolate tissue regions from WSIs at 40× magnification and then partitioned the tissue into non-overlapping 256 × 256-pixel patches using a sliding window strategy. A pretrained ResNet50 was employed to extract features from each patch, converting them into a 1,024-length feature vectors. These vectors were aggregated into a bag representing each slide for input into the MIL algorithm.

MIL algorithms such as ABMIL,[33] PatchGCN,[32] CLAM-SB,[28] TransMIL,[31] DSMIL,[29] and DTFDMIL[30] are widely applied in pathological diagnosis. Among these, ABMIL stands out for its simplicity and efficiency in allocating attention to critical instances. We selected it as the baseline model to compare its performance on the same dataset with the above mainstream methods, particularly using three feature extractor backbones (ResNet50,[34] PLIP,[38] and GigaPath[41]) for further evaluation (Tables 2 and S2; Figure S3). Additionally, we tested various ImageNet-based architectures and pathology-based models (DenseNet121,[35] EfficientNet,[36] Inception-ResNetV2,[37] PLIP,[38] UNI,[39] CONCH,[40] GigaPath,[41] and TITAN[42]) as feature extractors, comparing their performance with the features extracted by ResNet50 on the same internal test set (Tables S2 and S3). To mitigate domain shifts between natural and pathological images, ResNet50-extracted features were transformed into high-level features via a feature module. Each high-level feature was assigned an attention score from the attention module, and the fused slide-level features were generated by calculating the dot product of these attention scores and high-level features. Finally, these fused slide-level features were input into the classification module to assess inflammation, activity, atrophy, and IM.

In our analysis of images at different magnification levels, we observed that higher magnifications (40×) captured more detailed and semantically rich information, which facilitates more precise feature representation and significantly enhances model performance. In contrast, lower magnifications (e.g., 10× and 20×) provide a broader field of view, potentially aiding in the detection of large-scale tissue structure variations in CG. Therefore, we performed downsampling experiments on gastric pathological WSIs at different magnification levels (10×, 20×, and 40×) to identify the optimal sampling strategy.

The design of the objective function is critical for training MIL algorithms, as it directly influences performance and generalization. Most algorithms use cross-entropy loss, but this often biases results toward more populous classes due to class imbalance, overshadowing losses from minority classes and leading to suboptimal classifications. In our dataset (Table 1), particularly in the atrophy task (task 3), severe cases were underrepresented. Similar imbalances exist across other tasks. To mitigate this, we adopted gradient-Libra loss, which leverages the gradients to dynamically calibrate the hardness of different categories and rebalances the gradients of different samples, improving GastritisMIL's performance on long-tailed data.[50]

While mucosal structures of the gastric antrum and body share certain similarities in overall histology, they exhibit significant differences in cellular composition and functional roles. Considering that biopsy specimens from antrum account for the majority of clinical samples, we prioritized training GastritisMIL on a dataset of gastric antrum slides to ensure the model effectively captures critical features. 5-fold cross-validation was performed, and the best-performing fold model was subsequently fine-tuned on gastric body slides of 426 patients to evaluate its classification performance across four histological tasks (inflammation, activity, atrophy, and IM).

Interpretability

CNNs have demonstrated exceptional performance and robust interpretability for medical diagnoses. For instance, traditional visualization techniques such as CAM and Grad-CAM, though beneficial, produce abstract outputs that may challenge pathologists and are not applicable to MIL

methods.[51,52] In MIL frameworks, attention scores clarify the contributions of high-level features from specific patches to overall slide classification. Visualizing these attention scores as heatmaps highlights crucial tissue regions, thereby assisting pathologists in rapidly identifying and localizing critical diagnostic areas (Figure 4).

External testing

Due to variations in staining procedures and operational standards across four medical centers, models trained at one center might not perform as well at others. To thoroughly assess the model's generalization capabilities and enhance the credibility of results, we gathered data from three external test cohorts and compared the performance of GastritisMIL with that of three pathologists, providing a comprehensive evaluation of its effectiveness across different clinical settings (Figure 5; Tables S3–S6).

Statistical analysis

Continuous variables were presented as the mean ± standard deviation (SD). Pathology labels, annotated by two senior pathologists based on original reports, were displayed as semi-quantitative averages. The primary evaluation metrics for GastritisMIL are ACC, AUC, recall, precision, NPV, and F1 score. These assessments were conducted using the scikit-learn Python toolkit. Additionally, CUI was employed to assess clinical efficacy, categorizing scores into excellent (CUI $\geq$ 0.810), good (CUI $\geq$ 0.640), satisfactory (CUI $\geq$ 0.490), and poor utility (CUI < 0.490).[53] We employed DeLong's test to evaluate the differences in Macro-AUC performance across different feature extraction architectures at 40× magnification. And paired chi-squared tests (McNemar's test) were utilized to assess ACC differences between the model and the pathologists. Secondary metrics consisted of statistical p values and quadratic weighted kappa coefficients between the model and the three human pathologists across three external test cohorts. Moreover, to compare the differences in classification errors across multiple center cohorts, we employed the chi-squared test to assess the corresponding error proportion. Statistical significance was defined as $p < 0.050$. All analyses were performed using Python 3.9.20 with CUDA 11.1 support on a NVIDIA GTX 3090 GPU.

Ethics statement

As a retrospective study, informed consent from patients was waived. However, additional consent for the release of certain pathology data was obtained from the local Ethical Review Board of Zhongshan Hospital Affiliated to Xiamen University (XMZSYY-AF-SC-12-03). Data from the external test cohort were tentatively withheld to ensure privacy and confidentiality.

RESOURCE AVAILABILITY

Lead contact

Hongzhi Xu is the lead contact of this study and can be reached via email (xuhongzhi@xmu.edu.cn).

Materials availability

This study did not generate new materials.

Data and code availability

Our dataset required one and a half years of meticulous collection and rigorous review. This extensive effort has resulted in a valuable resource that bridges a gap in CG assessment. To enable future researchers to utilize a similar dataset for developing more efficient models and advancing data science, the data for which permission has been granted (800 WSIs and associated labels) can currently be released. To the best of our knowledge, these data will currently be the first and largest public collection of digital pathology slides in CG. The dataset, code, and model weights supporting the findings of this study are openly available in ScienceDB (https://doi.org/10.57760/sciencedb.19700) and on GitHub at https://github.com/nicedoctor123/Gastritis-MIL-pathology.

ACKNOWLEDGMENTS

We received support from the Xiamen Key Programs of Medicine and Health (3502Z20204007), the Natural Science Foundation of Xiamen City (3502Z202374017 and 3502Z20227351), the Xiamen High-Quality Development Young and Middle-Aged Backbone Talent Program (2024GZL-GG13), the Natural Science Foundation of Fujian Province (2024J011328), and the Science and Technology Programs of the Tan Kah Kee Innovation Laboratory (RD2022050901).

AUTHOR CONTRIBUTIONS

Conceptualization, H.X., K.X., S.C., and M. Lin; data curation, K.X., M. Lu, H.L., L.G., Y.L., X.L., X.Y., L. Xie, D.T., L. Xu, and Z.Z.; formal analysis, M. Lin, R.T., Y.Y., F.L., and Q.X.; funding acquisition, S.C., M. Lin, L.Z., and J.R.; investigation, K.X. and Y.H.; methodology, K.X. and Y.H.; project administration, L.W., J.R., and H.X.; resources, M. Lin, M. Lu, H.L., and L.G.; supervision, L.W., J.R., and H.X.; validation, K.X.; visualization, K.X.; writing – original draft, K.X.; writing – review & editing, K.X., Y.H., W.L., L.W., and H.X.

DECLARATION OF INTERESTS

The authors declare no competing interests.

SUPPLEMENTAL INFORMATION

Supplemental information can be found online at https://doi.org/10.1016/j.patter.2025.101286.

Received: January 1, 2025
Revised: February 11, 2025
Accepted: May 8, 2025
Published: June 9, 2025

REFERENCES

1. Jiang, J.-X., Liu, Q., Mao, X.-Y., Zhang, H.-H., Zhang, G.-X., and Xu, S.-F. (2016). Downward trend in the prevalence of Helicobacter pylori infections and corresponding frequent upper gastrointestinal diseases profile changes in Southeastern China between 2003 and 2012. SpringerPlus *5*, 1601. https://doi.org/10.1186/s40064-016-3185-2.
2. Chinese Society of Gastroenterology Cancer Collaboration Group of Chinese Society of Gastroenterology Chinese Medical Association (2023). Guidelines for diagnosis and treatment of chronic gastritis in China (2022, Shanghai). J. Dig. Dis. *24*, 150–180. https://doi.org/10.1111/1751-2980.13193.
3. Alenezy, A.K., and Hassan, T.M. (2014). Helicobacter pylori associated chronic gastritis endoscopic and pathological findings, comparative study. Int. J. Genet. Mol. Biol. *62*, 23–28. https://doi.org/10.5897/NGMB2014.0096.
4. den Hollander, W.J., Holster, I.L., den Hoed, C.M., Capelle, L.G., Tang, T. J., Anten, M.-P., Prytz-Berset, I., Witteman, E.M., Ter Borg, F., Hartog, G. d., et al. (2019). Surveillance of premalignant gastric lesions: a multicentre

prospective cohort study from low incidence regions. Gut *68*, 585–593. https://doi.org/10.1136/gutjnl-2017-314498.

5. Capelle, L.G., de Vries, A.C., Haringsma, J., Ter Borg, F., de Vries, R.A., Bruno, M.J., van Dekken, H., Meijer, J., van Grieken, N.C.T., and Kuipers, E.J. (2010). The staging of gastritis with the OLGA system by using intestinal metaplasia as an accurate alternative for atrophic gastritis. Gastrointest. Endosc. *71*, 1150–1158. https://doi.org/10.1016/j.gie.2009.12.029.
6. Deane, C., Kelly, O., and O'Morain, C. (2024). Disparities in Gastric Cancer Screening Worldwide. Cancer Screening and Prevention *3*, 184–190. https://doi.org/10.14218/CSP.2024.00008.
7. Correa, P. (1988). Chronic gastritis: a clinico-pathological classification. Am. J. Gastroenterol. *83*, 504–509.
8. Price, A.B., and Misiewicz, J.J. (1992). Grading and classification of chronic gastritis: the response of the working party. Gastroenterology *103*, 1116–1117. https://doi.org/10.1016/0016-5085(92)90066-8.
9. Dixon, M.F., Genta, R.M., Yardley, J.H., and Correa, P. (1996). Classification and grading of gastritis. The updated Sydney System. International Workshop on the Histopathology of Gastritis, Houston 1994. Am. J. Surg. Pathol. *20*, 1161–1181. https://doi.org/10.1097/00000478-199610000-00001.
10. Sugano, K., Tack, J., Kuipers, E.J., Graham, D.Y., El-Omar, E.M., Miura, S., Haruma, K., Asaka, M., Uemura, N., and Malfertheiner, P.; faculty members of Kyoto Global Consensus Conference (2015). Kyoto global consensus report on Helicobacter pylori gastritis. Gut *64*, 1353–1367. https://doi.org/10.1136/gutjnl-2015-309252.
11. Shah, S.C., Piazuelo, M.B., Kuipers, E.J., and Li, D. (2021). AGA Clinical Practice Update on the Diagnosis and Management of Atrophic Gastritis: Expert Review. Gastroenterology *161*, 1325–1332.e7. https://doi.org/10.1053/j.gastro.2021.06.078.
12. Metter, D.M., Colgan, T.J., Leung, S.T., Timmons, C.F., and Park, J.Y. (2019). Trends in the US and Canadian pathologist workforces from 2007 to 2017. JAMA Netw. Open *2*, e194337. https://doi.org/10.1001/jamanetworkopen.2019.4337.
13. Banks, M., Graham, D., Jansen, M., Gotoda, T., Coda, S., di Pietro, M., Uedo, N., Bhandari, P., Pritchard, D.M., Kuipers, E.J., et al. (2019). British Society of Gastroenterology guidelines on the diagnosis and management of patients at risk of gastric adenocarcinoma. Gut *68*, 1545–1575. https://doi.org/10.1136/gutjnl-2018-318126.
14. Pimentel-Nunes, P., Libânio, D., Marcos-Pinto, R., Areia, M., Leja, M., Esposito, G., Garrido, M., Kikuste, I., Megraud, F., Matysiak-Budnik, T., et al. (2019). Management of epithelial precancerous conditions and lesions in the stomach (MAPS II): European Society of Gastrointestinal Endoscopy (ESGE), European Helicobacter and Microbiota Study Group (EHMSG), European Society of Pathology (ESP), and Sociedade Portuguesa de Endoscopia Digestiva (SPED) guideline update 2019. Endoscopy *51*, 365–388. https://doi.org/10.1055/a-0859-1883.
15. Haug, C.J., and Drazen, J.M. (2023). Artificial Intelligence and Machine Learning in Clinical Medicine, 2023. N. Engl. J. Med. *388*, 1201–1208. 30. https://doi.org/10.1056/NEJMra2302038.
16. Tan, M., and Le, Q. (2021). Efficientnetv2: Smaller models and faster training. In International conference on machine learning (PMLR), pp. 10096–10106.
17. Jin, L., Shi, F., Chun, Q., Chen, H., Ma, Y., Wu, S., Hameed, N.U.F., Mei, C., Lu, J., Zhang, J., et al. (2021). Artificial intelligence neuropathologist for glioma classification using deep learning on hematoxylin and eosin stained slide images and molecular markers. Neuro Oncol. *23*, 44–52. 30. https://doi.org/10.1093/neuonc/noaa163.
18. van der Velden, B.H.M., Kuijf, H.J., Gilhuijs, K.G.A., and Viergever, M.A. (2022). Explainable artificial intelligence (XAI) in deep learning-based medical image analysis. Med. Image Anal. *79*, 102470. https://doi.org/10.1016/j.media.2022.102470.
19. Kather, J.N., Pearson, A.T., Halama, N., Jäger, D., Krause, J., Loosen, S. H., Marx, A., Boor, P., Tacke, F., Neumann, U.P., et al. (2019). Deep learning can predict microsatellite instability directly from histology in gastrointestinal cancer. Nat. Med. *25*, 1054–1056. https://doi.org/10.1038/s41591-019-0462-y.
20. Song, Z., Zou, S., Zhou, W., Huang, Y., Shao, L., Yuan, J., Gou, X., Jin, W., Wang, Z., Chen, X., et al. (2020). Clinically applicable histopathological diagnosis system for gastric cancer detection using deep learning. Nat. Commun. *11*, 4294. 27. https://doi.org/10.1038/s41467-020-18147-8.
21. Huang, B., Tian, S., Zhan, N., Ma, J., Huang, Z., Zhang, C., Zhang, H., Ming, F., Liao, F., Ji, M., et al. (2021a). Accurate diagnosis and prognosis prediction of gastric cancer using deep learning on digital pathological images: A retrospective multicentre study. EBioMedicine *73*, 103631. https://doi.org/10.1016/j.ebiom.2021.103631.
22. Ba, W., Wang, S., Shang, M., Zhang, Z., Wu, H., Yu, C., Xing, R., Wang, W., Wang, L., Liu, C., et al. (2022). Assessment of deep learning assistance for the pathological diagnosis of gastric cancer. Mod. Pathol. *35*, 1262–1268. https://doi.org/10.1038/s41379-022-01073-z.
23. Fu, B., Zhang, M., He, J., Cao, Y., Guo, Y., and Wang, R. (2022). StoHisNet: A hybrid multi-classification model with CNN and Transformer for gastric pathology images. Comput. Methods Progr. Biomed. *221*, 106924. https://doi.org/10.1016/j.cmpb.2022.106924.
24. Steinbuss, G., Kriegsmann, K., and Kriegsmann, M. (2020). Identification of Gastritis Subtypes by Convolutional Neuronal Networks on Histological Images of Antrum and Corpus Biopsies. Int. J. Mol. Sci. *21*, 6652. 11. https://doi.org/10.3390/ijms21186652.
25. Huang, D., Wang, Y., You, Q.H., Wang, X., Zhang, J.Y., Ding, X., Zhang, B. B., Cui, H.Y., Zhao, J.X., and Sheng, W.Q. (2021b). A novel attention fusion network-based multiple instance learning framework to automate diagnosis of chronic gastritis with multiple indicators. Zhonghua Bing li xue za zhi= Chinese Journal of Pathology *50*, 1116–1121. https://doi.org/10.3760/cma.j.cn112151-20210314-00204.
26. Braatz, J., Rajpurkar, P., Zhang, S., Ng, A.Y., and Shen, J. (2022). Deep Learning-Based Sparse Whole-Slide Image Analysis for the Diagnosis of Gastric Intestinal Metaplasia. Preprint at arXiv. https://arxiv.org/abs/2201.01449.
27. Lan, Y., Han, B., Zhai, T., Xu, Q., Li, Z., Liu, M., Xue, Y., and Xu, H. (2024). Clinical application of machine learning-based pathomics signature of gastric atrophy. Front. Oncol. *14*, 1289265. https://doi.org/10.3389/fonc.2024.1289265.
28. Lu, M.Y., Williamson, D.F.K., Chen, T.Y., Chen, R.J., Barbieri, M., and Mahmood, F. (2021). Data-efficient and weakly supervised computational pathology on whole-slide images. Nat. Biomed. Eng. *5*, 555–570. https://doi.org/10.1038/s41551-020-00682-w.
29. Li, B., Li, Y., and Eliceiri, K.W. (2021). Dual-stream multiple instance learning network for whole slide image classification with self-supervised contrastive learning. In Proceedings of the IEEE/CVF conference on computer vision and pattern recognition, pp. 14318–14328.
30. Zhang, H., Meng, Y., Zhao, Y., Qiao, Y., Yang, X., Coupland, S.E., and Zheng, Y. (2022). Dtfd-mil: Double-tier feature distillation multiple instance learning for histopathology whole slide image classification. In Proceedings of the IEEE/CVF conference on computer vision and pattern recognition, pp. 18802–18812.
31. Shao, Z., Bian, H., Chen, Y., Wang, Y., Zhang, J., Ji, X., and Zhang, Y. (2021). Transmil: Transformer based correlated multiple instance learning for whole slide image classification. Adv. Neural Inf. Process. Syst. *34*, 2136–2147.
32. Chen, R.J., Lu, M.Y., Shaban, M., Chen, C., Chen, T.Y., Williamson, D.F., and Mahmood, F. (2021). Whole slide images are 2d point clouds: Context-aware survival prediction using patch-based graph convolutional networks. In Medical Image Computing and Computer Assisted Intervention–MICCAI 2021: 24th International Conference, Strasbourg, France, September 27–October 1, 2021, Proceedings, Part VIII 24 (Springer), pp. 339–349.
33. Ilse, M., Tomczak, J.M., and Welling, M. (2018). Attention-based Deep Multiple Instance Learning. Preprint at arXiv. https://doi.org/10.48550/ARXIV.1802.04712.

34. He, K., Zhang, X., Ren, S., and Sun, J. (2016). Deep residual learning for image recognition. In Proceedings of the IEEE conference on computer vision and pattern recognition, pp. 770–778.

35. Huang, G., Liu, Z., Van Der Maaten, L., and Weinberger, K.Q. (2017). Densely connected convolutional networks. In Proceedings of the IEEE conference on computer vision and pattern recognition, pp. 4700–4708.

36. Tan, M., and Le, Q. (2019). Efficientnet: Rethinking model scaling for convolutional neural networks. In International conference on machine learning (PMLR), pp. 6105–6114.

37. Szegedy, C., Ioffe, S., Vanhoucke, V., and Alemi, A. (2017). Inception-v4, inception-resnet and the impact of residual connections on learning. In Proceedings of the AAAI Conference on Artificial Intelligence, *31*.

38. Huang, Z., Bianchi, F., Yuksekgonul, M., Montine, T.J., and Zou, J. (2023). A visual–language foundation model for pathology image analysis using medical Twitter. Nat. Med. *29*, 2307–2316. https://doi.org/10.1038/s41591-023-02504-3.

39. Chen, R.J., Ding, T., Lu, M.Y., Williamson, D.F.K., Jaume, G., Song, A.H., Chen, B., Zhang, A., Shao, D., Shaban, M., et al. (2024). Towards a General-Purpose Foundation Model for Computational Pathology. Nat. Med. *30*, 850–862. https://doi.org/10.1038/s41591-024-02857-3.

40. Lu, M.Y., Chen, B., Williamson, D.F.K., Chen, R.J., Liang, I., Ding, T., Jaume, G., Odintsov, I., Le, L.P., Gerber, G., et al. (2024). A visual-language foundation model for computational pathology. Nat. Med. *30*, 863–874. https://doi.org/10.1038/s41591-024-02856-4.

41. Xu, H., Usuyama, N., Bagga, J., Zhang, S., Rao, R., Naumann, T., Wong, C., Gero, Z., González, J., Gu, Y., et al. (2024). A whole-slide foundation model for digital pathology from real-world data. Nature *630*, 181–188. https://doi.org/10.1038/s41586-024-07441-w.

42. Ding, T., Wagner, S.J., Song, A.H., Chen, R.J., Lu, M.Y., Zhang, A., Vaidya, A.J., Jaume, G., Shaban, M., Kim, A., et al. (2024). Multimodal Whole Slide Foundation Model for Pathology. Preprint at arXiv. https://doi.org/10.48550/arXiv.2411.19666.

43. Fang, S., Liu, Z., Qiu, Q., Tang, Z., Yang, Y., Kuang, Z., Du, X., Xiao, S., Liu, Y., Luo, Y., et al. (2024). Diagnosing and grading gastric atrophy and intestinal metaplasia using semi-supervised deep learning on pathological images: development and validation study. Gastric Cancer: Official Journal of the International Gastric Cancer Association and the Japanese Gastric Cancer Association *27*, 343–354. https://doi.org/10.1007/s10120-023-01451-9.

44. Ma, M., Zeng, X., Qu, L., Sheng, X., Ren, H., Chen, W., Li, B., You, Q., Xiao, L., Wang, Y., et al. (2024). Advancing Automatic Gastritis Diagnosis: An Interpretable Multilabel Deep Learning Framework for the Simultaneous Assessment of Multiple Indicators. Am. J. Pathol. *194*, 1538–1549. https://doi.org/10.1016/j.ajpath.2024.04.007.

45. Reinhard, E., Adhikhmin, M., Gooch, B., and Shirley, P. (2001). Color transfer between images. IEEE Comput. Graph. Appl. *21*, 34–41. https://doi.org/10.1109/38.946629.

46. Macenko, M., Niethammer, M., Marron, J.S., Borland, D., Woosley, J.T., Guan, X., Schmitt, C., and Thomas, N.E. (2009). A method for normalizing histology slides for quantitative analysis. In 2009 IEEE international symposium on biomedical imaging: from nano to macro (IEEE), pp. 1107–1110.

47. Vahadane, A., Peng, T., Sethi, A., Albarqouni, S., Wang, L., Baust, M., Steiger, K., Schlitter, A.M., Esposito, I., and Navab, N. (2016). Structure-preserving color normalization and sparse stain separation for histological images. IEEE Trans. Med. Imag. *35*, 1962–1971. https://doi.org/10.1109/TMI.2016.2529665.

48. Kerdegari, H., Higgins, K., Veselkov, D., Laponogov, I., Polaka, I., Coimbra, M., Pescino, J.A., Leja, M., Dinis-Ribeiro, M., Kanonnikoff, T. F., et al. (2024). Foundational Models for Pathology and Endoscopy Images: Application for Gastric Inflammation. Preprint at arXiv. https://doi.org/10.48550/arXiv.2406.18249.

49. Xia, K., Hu, Y., Wang, L., and Xu, H. (2025). Comprehensive Assessment of Chronic Gastritis on WSI Data. Science Data Bank. https://doi.org/10.57760/sciencedb.19700.

50. Liu, M., Li, X., Gao, X., Chen, J., Shen, L., and Wu, H. (2022). Sample hardness based gradient loss for long-tailed cervical cell detection. In International Conference on Medical Image Computing and Computer-Assisted Intervention (Springer), pp. 109–119.

51. Zhou, B., Khosla, A., Lapedriza, A., Oliva, A., and Torralba, A. (2016). Learning deep features for discriminative localization. In Proceedings of the IEEE conference on computer vision and pattern recognition, pp. 2921–2929.

52. Selvaraju, R.R., Cogswell, M., Das, A., Vedantam, R., Parikh, D., and Batra, D. (2020). Grad-CAM: Visual Explanations from Deep Networks via Gradient-Based Localization. Int. J. Comput. Vis. *128*, 336–359. https://doi.org/10.1007/s11263-019-01228-7.

53. Østergaard, S.D., Dinesen, P.T., and Foldager, L. (2010). Quantifying the value of markers in screening programmes. Eur. J. Epidemiol. *25*, 151–154. https://doi.org/10.1007/s10654-010-9430-z.

Patterns, Volume *6*

Supplemental information

GastritisMIL: An interpretable deep learning model for the comprehensive histological assessment of chronic gastritis

Kun Xia, Yihuang Hu, Shuntian Cai, Mengjie Lin, Mingzhi Lu, Huadong Lu, Yuhan Ye, Fenglian Lin, Liang Gao, Qingan Xia, Ruihua Tian, Weiping Lin, Lei Xie, Decheng Tan, Yapi Lu, Xunting Lin, Xiaoning Yang, Lingfeng Zhong, Lei Xu, Zhixin Zhang, Liansheng Wang, Jianlin Ren, and Hongzhi Xu

Supplemental Figures

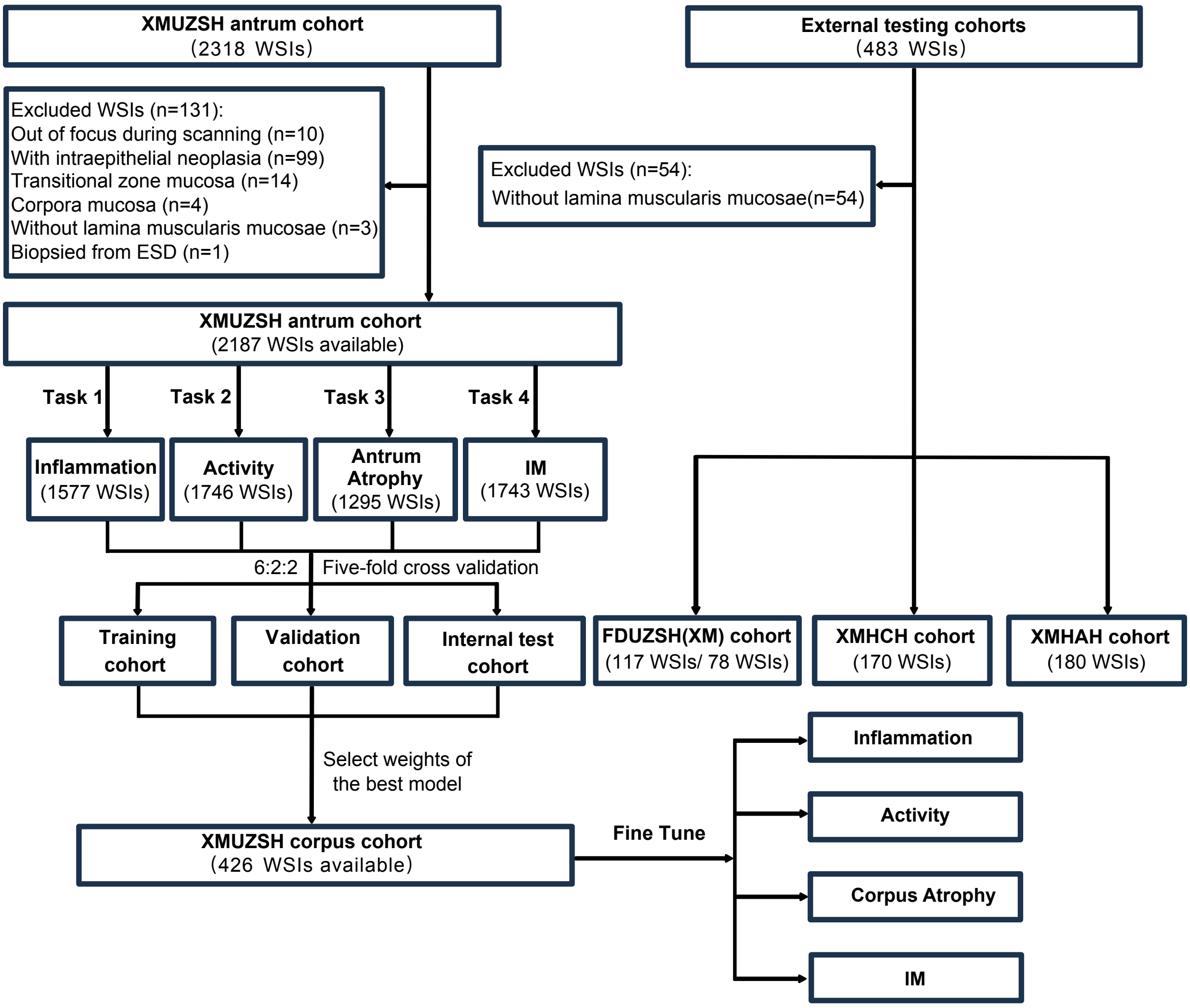

Figure S1. Overview of the study design. WSIs = whole slide images. XMUZSH = Zhongshan Hospital of the Xiamen University. FDUZSH(XM) = Zhongshan Hospital, Fudan University (Xiamen Branch). XMHCH = Xiamen Haicang Hospital. XMHAH = Xiamen Humanity Hospital. IM = Intestinal Metaplasia.

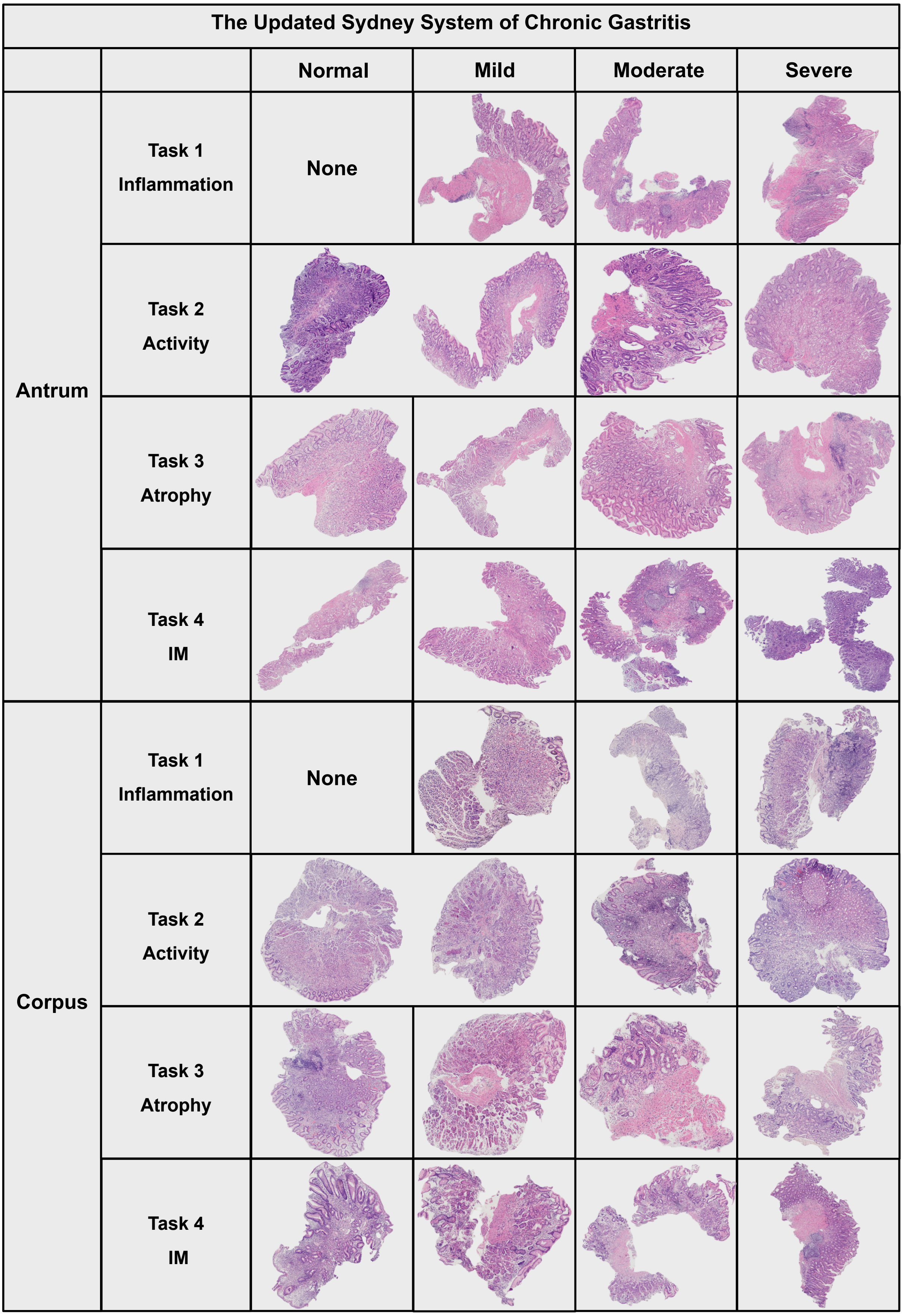

Figure S2. Detailed examples of the updated Sydney system for chronic gastritis.

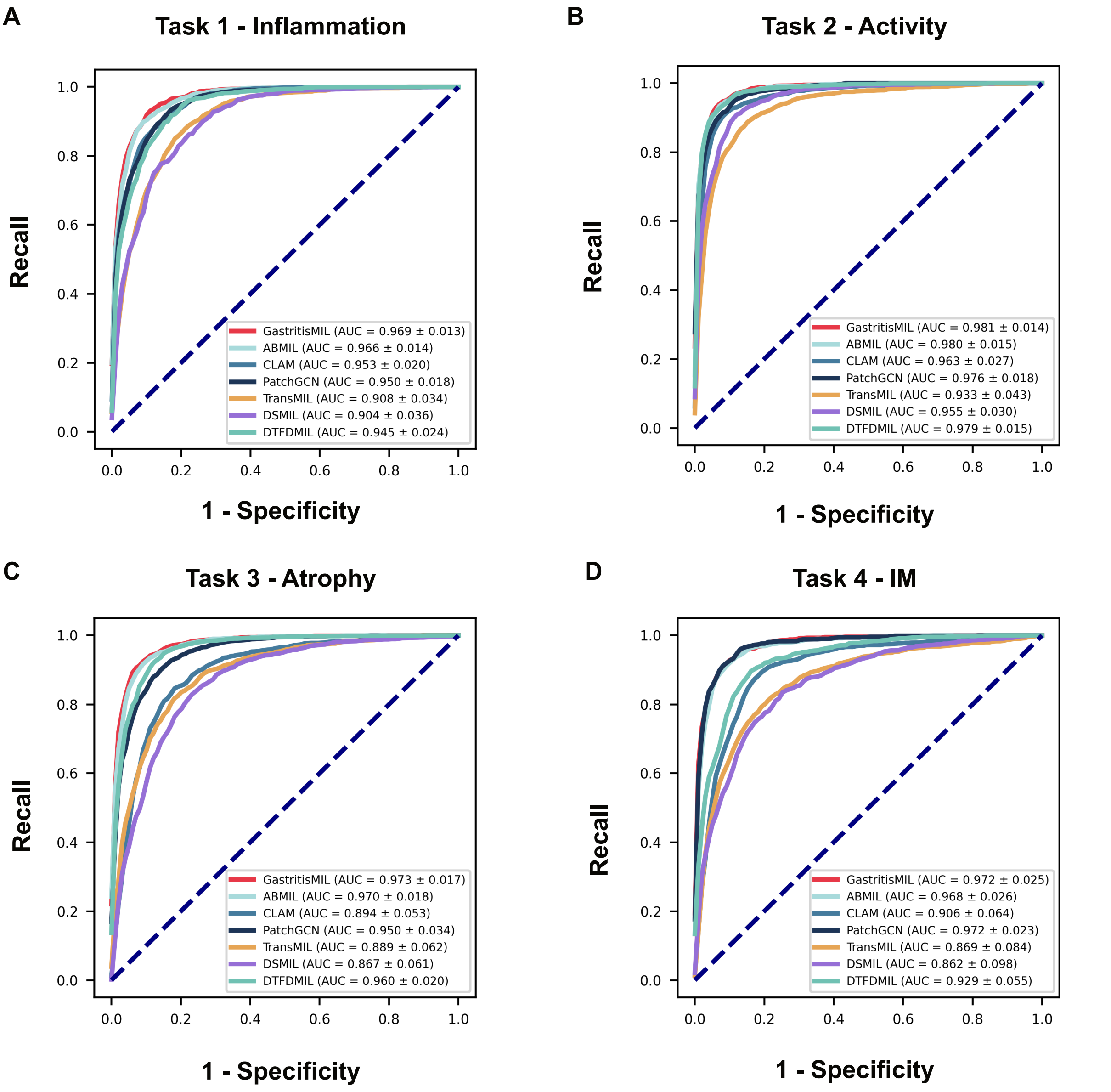

Figure S3. Comparison of ROC curves between GastritisMIL and another five frameworks utilizing ResNet50 at 40x magnification (ABMIL, CLAM, PatchGCN, TransMIL, DSMIL and DTFDMIL.)

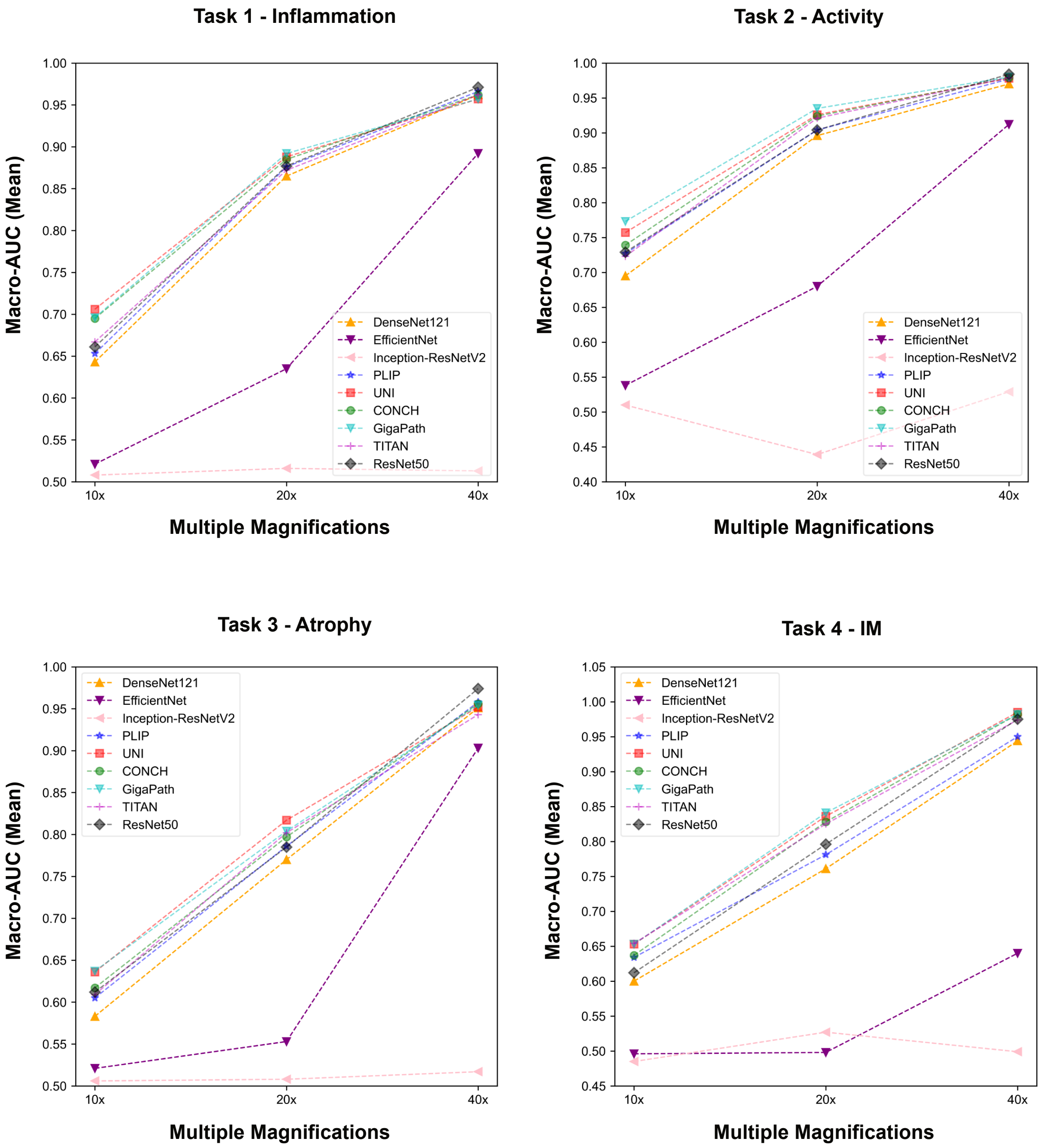

Figure S4. Comparison of Macro-AUC (Mean) among different feature extraction backbones (DenseNet121, EfficientNet, Inception-ResNetV2, PLIP, UNI, CONCH, GigaPath, TITAN and ResNet50) at multiple magnifications (10x, 20x and 40x).

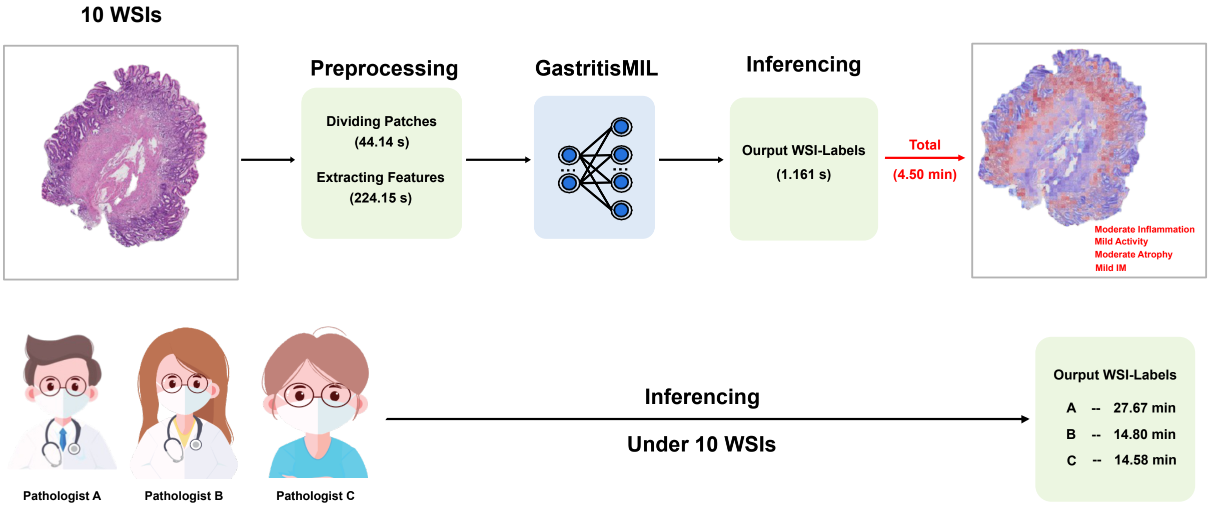

Figure S5. Comparison of judgement time on an independent external test set from 10 WSIs.

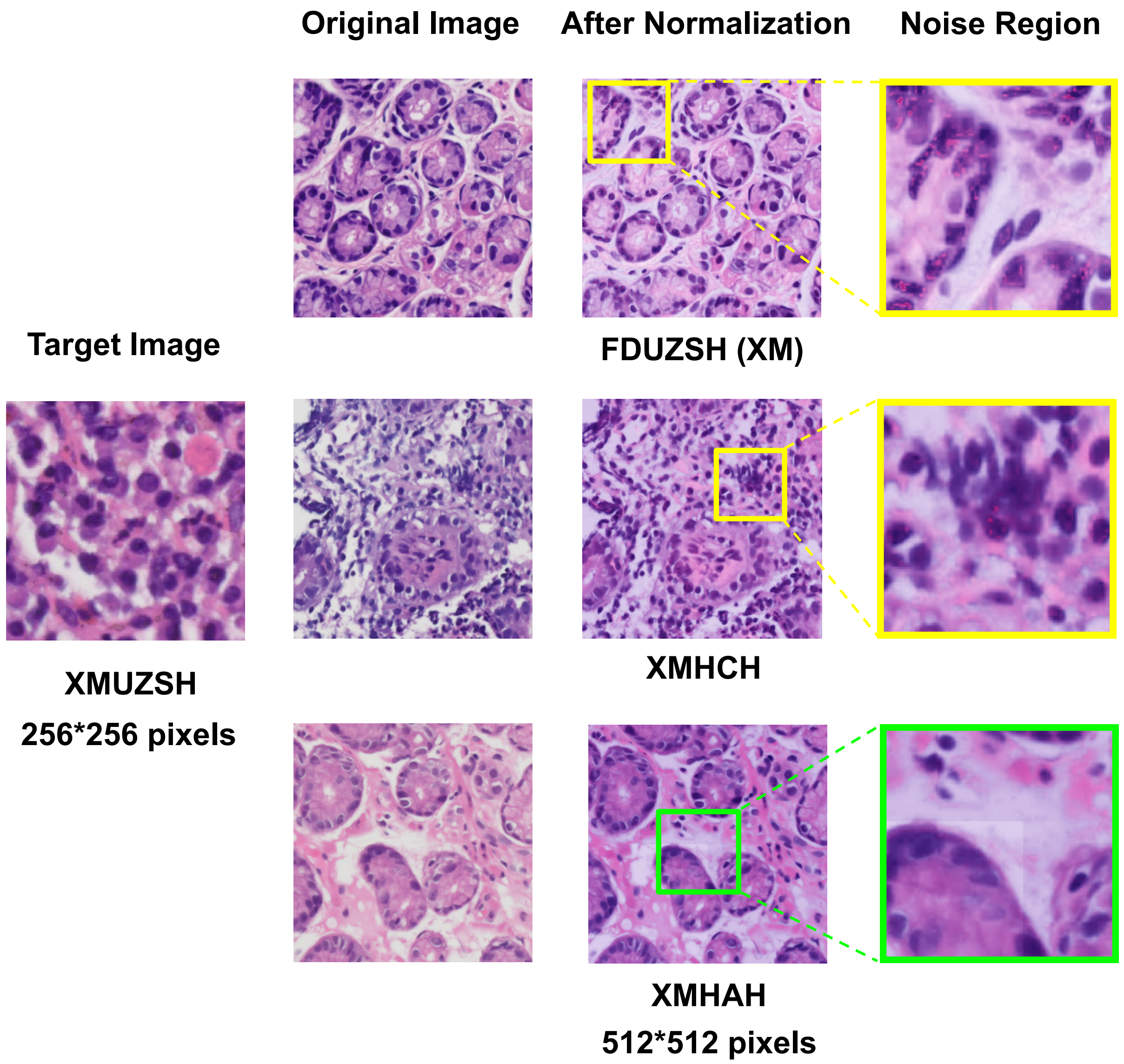

Figure S6. Comparison of the effect of coloring normalization (Macenko method) on our dataset. The green box represents noticeable tiling artifacts. The yellow box represents significant noise in some scaled images.

Supplemental Tables

XMUZSH Antrum Cohort	Task 1 - Inflammation	Task 2 - Activity	Task 3 - Antrum Atrophy	Task 4 - IM
**AUC**	0.971 ± 0.003	0.984 ± 0.003	0.974 ± 0.003	0.975 ± 0.003
**Accuracy**	0.892 ± 0.006	0.922 ± 0.006	0.866 ± 0.009	0.887 ± 0.005
**Precision**	0.842 ± 0.018	0.898 ± 0.012	0.872 ± 0.012	0.850 ± 0.010
**Recall**	0.824 ± 0.006	0.898 ± 0.010	0.834 ± 0.018	0.808 ± 0.010
**F1-score**	0.832 ± 0.009	0.900 ± 0.010	0.846 ± 0.013	0.818 ± 0.008
**CUI+**	0.706 ± 0.013	0.812 ± 0.017	0.725 ± 0.020	0.691 ± 0.013
**CUI-**	0.765 ± 0.007	0.860 ± 0.012	0.794 ± 0.020	0.761 ± 0.012
**XMUZSH Corpus Cohort**	**Task 1 - Inflammation**	**Task 2 - Activity**	**Task 3 - Corpus Atrophy**	**Task 4 - IM**
**AUC**	0.980 ± 0.008	0.977 ± 0.008	0.977 ± 0.005	0.989 ± 0.005
**Accuracy**	0.906 ± 0.013	0.942 ± 0.010	0.920 ± 0.009	0.962 ± 0.004
**Precision**	0.900 ± 0.006	0.910 ± 0.025	0.852 ± 0.019	0.890 ± 0.018
**Recall**	0.878 ± 0.045	0.874 ± 0.040	0.832 ± 0.041	0.786 ± 0.021
**F1-score**	0.872 ± 0.030	0.870 ± 0.034	0.830 ± 0.032	0.790 ± 0.024
**CUI+**	0.783 ± 0.045	0.794 ± 0.047	0.719 ± 0.049	0.685 ± 0.029
**CUI-**	0.811 ± 0.051	0.826 ± 0.035	0.788 ± 0.042	0.777 ± 0.023

Table S1. Five-fold cross-validation performance of GastritisMIL on the XMUZSH antrum cohort and the fine-tuned model on the XMUZSH corpus cohort.
Data are represented as mean ± SD. AUC = area under the receiver-operating characteristic curve. CUI+= positive clinical utility index. CUI- = negative clinical utility index.

Pathology feature extraction backbone - PLIP						
**Method**	**Task 1 - Inflammation**			**Task 2 - Activity**		
	**AUC**	**ACC**	**F1-score**	**AUC**	**ACC**	**F1-score**
**CLAM-SB**	0.961 ± 0.003	0.874 ± 0.004	0.776 ± 0.013	0.975 ± 0.004	0.893 ± 0.010	0.850 ± 0.017
**DSMIL**	0.934 ± 0.006	0.848 ± 0.006	0.726 ± 0.018	0.965 ± 0.004	0.872 ± 0.009	0.822 ± 0.014
**DTFDMIL**	0.945 ± 0.007	0.845 ± 0.004	0.610 ± 0.022	**0.979 ± 0.003**	**0.904 ± 0.005**	**0.869 ± 0.010**
**TransMIL**	0.948 ± 0.007	0.855 ± 0.007	0.762 ± 0.027	0.967 ± 0.004	0.882 ± 0.010	0.835 ± 0.014
**PatchGCN**	0.962 ± 0.002	0.874 ± 0.002	0.796 ± 0.016	0.973 ± 0.002	0.889 ± 0.007	0.850 ± 0.011
**ABMIL**	<u>0.965 ± 0.003</u>	<u>0.883 ± 0.007</u>	**0.814 ± 0.018**	0.976 ± 0.002	0.896 ± 0.006	0.857 ± 0.009
**GastritisMIL (Ours)**	**0.966 ± 0.002**	**0.883 ± 0.005**	<u>0.806 ± 0.009</u>	<u>0.977 ± 0.003</u>	<u>0.900 ± 0.010</u>	<u>0.864 ± 0.015</u>
**Method**	**Task 3 - Antrum Atrophy**			**Task 4 - Intestinal Metaplasia**		
	**AUC**	**ACC**	**F1-score**	**AUC**	**ACC**	**F1-score**
**CLAM-SB**	0.936 ± 0.006	0.781 ± 0.014	0.710 ± 0.024	0.909 ± 0.004	0.802 ± 0.005	0.619 ± 0.011
**DSMIL**	0.917 ± 0.004	0.730 ± 0.003	0.623 ± 0.014	0.912 ± 0.004	0.802 ± 0.007	0.643 ± 0.010
**DTFDMIL**	0.934 ± 0.006	0.769 ± 0.013	0.583 ± 0.007	0.908 ± 0.011	0.784 ± 0.008	0.575 ± 0.017
**TransMIL**	0.912 ± 0.007	0.700 ± 0.015	0.618 ± 0.036	0.924 ± 0.002	0.805 ± 0.007	0.664 ± 0.015
**PatchGCN**	0.953 ± 0.004	0.801 ± 0.006	<u>0.776 ± 0.011</u>	**0.959 ± 0.003**	**0.881 ± 0.003**	**0.804 ± 0.005**
**ABMIL**	<u>0.957 ± 0.004</u>	**0.822 ± 0.011**	**0.793 ± 0.019**	<u>0.953 ± 0.003</u>	<u>0.854 ± 0.010</u>	<u>0.753 ± 0.018</u>
**GastritisMIL (Ours)**	**0.958 ± 0.005**	<u>0.805 ± 0.014</u>	0.753 ± 0.034	0.950 ± 0.003	0.846 ± 0.008	0.753 ± 0.019
**Pathology feature extraction backbone - GigaPath**						
**Method**	**Task 1 - Inflammation**			**Task 2 - Activity**		
	**AUC**	**ACC**	**F1-score**	**AUC**	**ACC**	**F1-score**
**CLAM-SB**	**0.960 ± 0.003**	0.856 ± 0.005	0.760 ± 0.016	0.978 ± 0.002	0.904 ± 0.007	0.860 ± 0.010
**DSMIL**	0.951 ± 0.005	0.856 ± 0.005	0.738 ± 0.017	0.975 ± 0.003	0.906 ± 0.006	0.875 ± 0.008
**DTFDMIL**	0.937 ± 0.006	0.832 ± 0.006	0.690 ± 0.031	<u>0.981 ± 0.003</u>	**0.918 ± 0.010**	**0.882 ± 0.017**
**TransMIL**	0.880 ± 0.005	0.788 ± 0.005	0.602 ± 0.024	0.958 ± 0.007	0.868 ± 0.012	0.814 ± 0.018
**PatchGCN**	<u>0.959 ± 0.004</u>	**0.866 ± 0.002**	0.750 ± 0.024	<u>0.981 ± 0.003</u>	0.912 ± 0.008	0.874 ± 0.012
**ABMIL**	0.959 ± 0.005	0.854 ± 0.013	<u>0.762 ± 0.015</u>	**0.983 ± 0.003**	0.910 ± 0.009	0.876 ± 0.011
**GastritisMIL (Ours)**	0.957 ± 0.005	<u>0.866 ± 0.008</u>	**0.796 ± 0.012**	0.980 ± 0.002	<u>0.912 ± 0.006</u>	<u>0.880 ± 0.010</u>
**Method**	**Task 3 - Antrum Atrophy**			**Task 4 - Intestinal Metaplasia**		
	**AUC**	**ACC**	**F1-score**	**AUC**	**ACC**	**F1-score**
**CLAM-SB**	0.891 ± 0.021	0.702 ± 0.027	0.610 ± 0.047	0.910 ± 0.042	0.824 ± 0.037	0.640 ± 0.076
**DSMIL**	0.928 ± 0.005	0.753 ± 0.010	0.679 ± 0.018	0.969 ± 0.003	0.892 ± 0.007	0.790 ± 0.014
**DTFDMIL**	0.934 ± 0.003	0.776 ± 0.005	0.698 ± 0.012	0.969 ± 0.006	0.888 ± 0.007	0.776 ± 0.018
**TransMIL**	0.830 ± 0.008	0.574 ± 0.015	0.514 ± 0.025	0.946 ± 0.004	0.839 ± 0.009	0.680 ± 0.017
**PatchGCN**	0.948 ± 0.002	0.786 ± 0.004	0.760 ± 0.014	0.982 ± 0.003	0.912 ± 0.011	0.838 ± 0.017
**ABMIL**	**0.954 ± 0.003**	<u>0.796 ± 0.010</u>	<u>0.770 ± 0.014</u>	**0.983 ± 0.002**	**0.924 ± 0.005**	**0.858 ± 0.011**
**GastritisMIL (Ours)**	<u>0.954 ± 0.004</u>	**0.814 ± 0.010**	**0.796 ± 0.014**	<u>0.982 ± 0.001</u>	<u>0.918 ± 0.008</u>	<u>0.852 ± 0.014</u>

Table S2. Diagnostic performance of GastritisMIL in XMUZSH antrum cohort utilizing PLIP and GigaPath (internal test cohort) at 40x magnification.

Data are represented as mean ± SD. The best values are **highlighted**. The second-best values are <u>underlined</u>. AUC = area under the receiver-operating characteristic curve.

Comparison of different pathology feature extraction backbones at 40x								
Method	Task 1 - Inflammation				Task 2 - Activity			
	AUC	ACC	F1-score	P-value*	AUC	ACC	F1-score	P-value*
**DenseNet121**	0.962 ± 0.002	0.870 ± 0.004	0.796 ± 0.013	0.069	0.970 ± 0.004	0.893 ± 0.010	0.851 ± 0.015	0.006
**EfficientNet**	0.892 ± 0.017	0.800 ± 0.004	0.659 ± 0.009	<0.001	0.912 ± 0.002	0.797 ± 0.007	0.698 ± 0.009	<0.001
**Inception-ResNetV2**	0.513 ± 0.028	0.608 ± 0.001	0.252 ± 0.000	<0.001	0.529 ± 0.014	0.618 ± 0.001	0.255 ± 0.000	<0.001
**PLIP**	<u>0.966 ± 0.002</u>	<u>0.883 ± 0.005</u>	<u>0.806 ± 0.009</u>	0.498	0.977 ± 0.003	0.900 ± 0.010	0.864 ± 0.015	0.169
**UNI**	0.957 ± 0.003	0.864 ± 0.005	0.782 ± 0.011	0.014	0.978 ± 0.004	0.911 ± 0.010	0.877 ± 0.014	0.022
**CONCH**	0.962 ± 0.003	0.880 ± 0.005	0.797 ± 0.012	0.190	0.979 ± 0.003	0.906 ± 0.008	0.874 ± 0.011	0.227
**GigaPath**	0.957 ± 0.005	0.866 ± 0.008	0.796 ± 0.012	<0.001	<u>0.980 ± 0.002</u>	<u>0.912 ± 0.006</u>	<u>0.880 ± 0.010</u>	0.355
**TITAN**	0.962 ± 0.004	0.877 ± 0.005	0.796 ± 0.016	0.082	0.979 ± 0.002	0.907 ± 0.006	0.872 ± 0.011	0.380
**ResNet50**	**0.971 ± 0.003**	**0.892 ± 0.006**	**0.832 ± 0.009**	**reference**	**0.984 ± 0.003**	**0.922 ± 0.006**	**0.900 ± 0.010**	**reference**
Method	Task 3 - Antrum Atrophy				Task 4 - Intestinal Metaplasia			
	AUC	ACC	F1-score	P-value*	AUC	ACC	F1-score	P-value*
**DenseNet121**	0.951 ± 0.004	0.795 ± 0.010	0.752 ± 0.022	0.006	0.944 ± 0.004	0.839 ± 0.002	0.730 ± 0.006	<0.001
**EfficientNet**	0.903 ± 0.004	0.702 ± 0.008	0.645 ± 0.012	<0.001	0.640 ± 0.028	0.665 ± 0.003	0.224 ± 0.016	<0.001
**Inception-ResNetV2**	0.517 ± 0.010	0.389 ± 0.001	0.140 ± 0.000	<0.001	0.499 ± 0.016	0.664 ± 0.000	0.200 ± 0.000	<0.001
**PLIP**	<u>0.958 ± 0.005</u>	0.805 ± 0.014	0.753 ± 0.034	0.028	0.950 ± 0.003	0.846 ± 0.008	0.753 ± 0.019	<0.001
**UNI**	0.952 ± 0.006	0.796 ± 0.017	0.765 ± 0.023	<0.001	**0.985 ± 0.003**	0.931 ± 0.009	0.867 ± 0.015	0.024
**CONCH**	0.956 ± 0.004	0.797 ± 0.012	0.772 ± 0.007	0.008	0.982 ± 0.003	<u>0.917 ± 0.007</u>	<u>0.852 ± 0.009</u>	0.211
**GigaPath**	0.954 ± 0.004	<u>0.814 ± 0.010</u>	<u>0.796 ± 0.014</u>	<0.001	<u>0.982 ± 0.001</u>	**0.918 ± 0.008**	**0.852 ± 0.014**	0.376
**TITAN**	0.943 ± 0.006	0.775 ± 0.020	0.753 ± 0.023	<0.001	0.974 ± 0.002	0.905 ± 0.005	0.826 ± 0.008	0.843
**ResNet50**	**0.974 ± 0.003**	**0.866 ± 0.009**	**0.846 ± 0.013**	**reference**	0.975 ± 0.003	0.887 ± 0.005	0.818 ± 0.008	**reference**

Table S3. Diagnostic performance of GastritisMIL in XMUZSH antrum cohort utilizing different pathology feature extraction backbones (internal test cohort) at 40x magnification.

Data are represented as mean ± SD. The best values are **highlighted**. The second-best values are <u>underlined</u>. The P-value* indicates the difference in AUC between ResNet50 and another backbone, as determined by the DeLong's test. AUC = area under the receiver-operating characteristic curve.

Comparison of different magnification for feature extraction						
Magification	Task 1 - Inflammation			Task 2 - Activity		
	AUC	ACC	F1-score	AUC	ACC	F1-score
**10x**	0.661 ± 0.022	0.644 ± 0.006	0.380 ± 0.004	0.729 ± 0.013	0.674 ± 0.006	0.475 ± 0.013
**20x**	0.877 ± 0.006	0.765 ± 0.009	0.589 ± 0.024	0.904 ± 0.003	0.798 ± 0.008	0.709 ± 0.013
**40x**	**0.971 ± 0.003**	**0.892 ± 0.006**	**0.832 ± 0.009**	**0.984 ± 0.003**	**0.922 ± 0.006**	**0.900 ± 0.010**
Magification	Task 3 - Antrum Atrophy			Task 4 - Intestinal Metaplasia		
	AUC	ACC	F1-score	AUC	ACC	F1-score
**10x**	0.612 ± 0.020	0.449 ± 0.007	0.272 ± 0.010	0.612 ± 0.011	0.664 ± 0.005	0.284 ± 0.008
**20x**	0.785 ± 0.008	0.574 ± 0.022	0.441 ± 0.034	0.796 ± 0.007	0.729 ± 0.002	0.468 ± 0.008
**40x**	**0.974 ± 0.003**	**0.866 ± 0.009**	**0.846 ± 0.013**	**0.975 ± 0.003**	**0.887 ± 0.005**	**0.818 ± 0.008**

Table S4. Diagnostic performance of GastritisMIL in XMUZSH antrum cohort utilizing different magnification (internal test cohort).

Data are represented as mean ± SD. The best values are **highlighted**. The second-best values are underlined. AUC = area under the receiver-operating characteristic curve.

External Testing		AUC	Accuracy	F1-score	Precision	Recall	NPV
Task 1 - Inflammation	FDUZSH (XM)	0.937 ± 0.018	0.880 ± 0.029	0.914 ± 0.021	0.936 ± 0.015	0.912 ± 0.020	1.000 ± 0.085
	XMHCH	0.924 ± 0.017	0.829 ± 0.029	0.812 ± 0.035	0.877 ± 0.027	0.775 ± 0.039	0.940 ± 0.022
	XMHAH	0.913 ± 0.017	0.806 ± 0.030	0.631 ± 0.050	0.880 ± 0.114	0.633 ± 0.033	0.921 ± 0.068
Task 2 - Activity	FDUZSH (XM)	0.931 ± 0.018	0.829 ± 0.034	0.652 ± 0.061	0.621 ± 0.065	0.736 ± 0.040	1.000 ± 0.117
	XMHCH	0.923 ± 0.015	0.824 ± 0.029	0.785 ± 0.035	0.804 ± 0.034	0.800 ± 0.031	1.000 ± 0.064
	XMHAH	0.901 ± 0.017	0.800 ± 0.029	0.738 ± 0.036	0.754 ± 0.037	0.751 ± 0.032	1.000 ± 0.069
Task 3 - Atrophy	FDUZSH (XM)	0.938 ± 0.019	0.821 ± 0.044	0.811 ± 0.049	0.810 ± 0.052	0.814 ± 0.049	0.940 ± 0.037
	XMHCH	0.865 ± 0.024	0.735 ± 0.034	0.693 ± 0.051	0.828 ± 0.046	0.664 ± 0.044	0.952 ± 0.049
	XMHAH	0.883 ± 0.018	0.750 ± 0.031	0.725 ± 0.037	0.782 ± 0.031	0.731 ± 0.034	0.831 ± 0.053
Task 4 - IM	FDUZSH (XM)	0.952 ± 0.017	0.833 ± 0.042	0.816 ± 0.047	0.814 ± 0.048	0.819 ± 0.048	0.952 ± 0.008
	XMHCH	0.908 ± 0.016	0.812 ± 0.030	0.647 ± 0.065	0.754 ± 0.057	0.604 ± 0.064	0.970 ± 0.042
	XMHAH	0.928 ±0.015	0.800 ± 0.031	0.719 ± 0.040	0.725 ± 0.040	0.728 ± 0.042	0.924 ± 0.019

Table S5. AUC, accuracy, F1-score, precision, recall, NPV of our model (GastritisMIL) in the three external cohorts with images at 40x magnification.

Data are represented as mean ± SD. AUC = area under the receiver-operating characteristic curve

External Testing		Accuracy	F1-score	Precision	Recall	P-value	Kappa#
Task 1 - Inflammation	**GastritisMIL**	0.880 ± 0.029	0.914 ± 0.021	0.936 ± 0.015	0.912 ± 0.020	reference	reference
	**Junior Pathologist A**	0.803 ± 0.037	0.707 ± 0.087	0.773 ± 0.112	0.686 ± 0.084	0.188	0.526
	**Expert Pathologist B**	0.846 ± 0.034	0.792 ± 0.059	0.768 ± 0.056	0.898 ± 0.036	0.597	0.634
	**Expert Pathologist C**	0.872 ± 0.031	0.762 ± 0.072	0.743 ± 0.074	0.791 ± 0.087	1.000	0.656
Task 2 - Activity	**GastritisMIL**	0.829 ± 0.034	0.652 ± 0.059	0.621 ± 0.063	0.736 ± 0.037	reference	reference
	**Junior Pathologist A**	0.803 ± 0.039	0.397 ± 0.041	0.397 ± 0.051	0.405 ± 0.037	0.678	0.357
	**Expert Pathologist B**	0.906 ± 0.027	0.802 ± 0.069	0.816 ± 0.069	0.814 ± 0.071	0.122	0.714
	**Expert Pathologist C**	0.932 ± 0.023	0.875 ± 0.055	0.895 ± 0.037	0.879 ± 0.060	0.029	0.724
Task 3 - Atrophy	**GastritisMIL**	0.821 ± 0.043	0.811 ± 0.048	0.810 ± 0.050	0.814 ± 0.048	reference	reference
	**Junior Pathologist A**	0.641 ± 0.055	0.647 ± 0.056	0.634 ± 0.056	0.695 ± 0.051	0.038	0.719
	**Expert Pathologist B**	0.897 ± 0.034	0.908 ± 0.033	0.903 ± 0.036	0.915 ± 0.032	0.263	0.825
	**Expert Pathologist C**	0.872 ± 0.037	0.865 ± 0.043	0.868 ± 0.043	0.872 ± 0.040	0.503	0.825
Task 4 - IM	**GastritisMIL**	0.833 ± 0.042	0.816 ± 0.047	0.814 ± 0.048	0.819 ± 0.048	reference	reference
	**Junior Pathologist A**	0.833 ± 0.044	0.803 ± 0.049	0.819 ± 0.045	0.815 ± 0.044	1.000	0.878
	**Expert Pathologist B**	0.833 ± 0.043	0.806 ± 0.049	0.810 ± 0.049	0.812 ± 0.047	1.000	0.872
	**Expert Pathologist C**	0.872 ± 0.037	0.848 ± 0.045	0.855 ± 0.044	0.850 ± 0.043	0.607	0.921

Table S6. Accuracy, F1-score, precision, recall of our model (GastritisMIL) and human Pathologists in the external test cohort of FDUZSH (XM).

Data are represented as mean ± SD.

* Difference of accuracy between GastritisMIL and human Pathologists, tested by Paired chi-square test (McNamar’s test).

# inter-observer agreement of GastritisMIL and each human Pathologist, evaluated by quadratic weighted kappa coefficient.

External Testing		Accuracy	F1-score	Precision	Recall	P-value*	Kappa#
Task 1 - Inflammation	**GastritisMIL**	0.829 ± 0.029	0.812 ± 0.035	0.877 ± 0.027	0.775 ± 0.039	reference	reference
	**Junior Pathologist A**	0.588 ± 0.039	0.603 ± 0.039	0.596 ± 0.041	0.616 ± 0.042	<0.001	0.497
	**Expert Pathologist B**	0.800 ± 0.030	0.800 ± 0.030	0.783 ± 0.030	0.868 ± 0.019	0.568	0.696
	**Expert Pathologist C**	0.859 ± 0.028	0.836 ± 0.034	0.852 ± 0.035	0.826 ± 0.036	0.542	0.665
Task 2 - Activity	**GastritisMIL**	0.824 ± 0.029	0.785 ± 0.035	0.804 ± 0.034	0.800 ± 0.031	reference	reference
	**Junior Pathologist A**	0.641 ± 0.038	0.498 ± 0.043	0.624 ± 0.049	0.507 ± 0.034	<0.001	0.358
	**Expert Pathologist B**	0.829 ± 0.030	0.794 ± 0.035	0.791 ± 0.035	0.798 ± 0.035	1.000	0.752
	**Expert Pathologist C**	0.853 ± 0.027	0.819 ± 0.032	0.850 ± 0.024	0.829 ± 0.030	0.568	0.718
Task 3 - Atrophy	**GastritisMIL**	0.735 ± 0.034	0.693 ± 0.051	0.828 ± 0.046	0.664 ± 0.044	reference	reference
	**Junior Pathologist A**	0.671 ± 0.035	0.549 ± 0.042	0.587 ± 0.054	0.550 ± 0.037	0.260	0.578
	**Expert Pathologist B**	0.724 ± 0.036	0.700 ± 0.042	0.685 ± 0.042	0.753 ± 0.036	0.915	0.606
	**Expert Pathologist C**	0.753 ± 0.033	0.735 ± 0.038	0.717 ± 0.038	0.782 ± 0.033	0.824	0.568
Task 4 - IM	**GastritisMIL**	0.812 ± 0.030	0.647 ± 0.067	0.754 ± 0.054	0.604 ± 0.065	reference	reference
	**Junior Pathologist A**	0.859 ± 0.026	0.560 ± 0.060	0.754 ± 0.131	0.548 ± 0.049	0.200	0.687
	**Expert Pathologist B**	0.859 ± 0.027	0.684 ± 0.060	0.692 ± 0.061	0.683 ± 0.064	0.200	0.775
	**Expert Pathologist C**	0.847 ± 0.027	0.746 ± 0.048	0.746 ± 0.044	0.821 ± 0.043	0.392	0.700

Table S7. Accuracy, F1-score, precision, recall of our model (GastritisMIL) and human Pathologists in the external test cohort of XMHCH.

Data are represented as mean ± SD.

* Difference of accuracy between GastritisMIL and human Pathologists, tested by Paired chi-square test (McNamar's test).

# inter-observer agreement of GastritisMIL and each human Pathologist, evaluated by quadratic weighted kappa coefficient.

External Testing		Accuracy	F1-score	Precision	Recall	P-value*	Kappa#
Task 1 - Inflammation	**GastritisMIL**	0.806 ± 0.030	0.631 ± 0.050	0.880 ± 0.114	0.633 ± 0.033	reference	reference
	**Junior Pathologist A**	0.794 ± 0.031	0.756 ± 0.043	0.785 ± 0.046	0.736 ± 0.047	0.892	0.536
	**Expert Pathologist B**	0.789 ± 0.031	0.706 ± 0.049	0.766 ± 0.056	0.677 ± 0.048	0.788	0.493
	**Expert Pathologist C**	0.756 ± 0.031	0.772 ± 0.034	0.773 ± 0.036	0.837 ± 0.020	0.262	0.614
Task 2 - Activity	**GastritisMIL**	0.800 ± 0.029	0.738 ± 0.036	0.754 ± 0.037	0.751 ± 0.032	reference	reference
	**Junior Pathologist A**	0.650 ± 0.036	0.528 ± 0.041	0.671 ± 0.031	0.536 ± 0.035	0.003	0.403
	**Expert Pathologist B**	0.861 ± 0.026	0.831 ± 0.032	0.842 ± 0.029	0.833 ± 0.031	0.169	0.677
	**Expert Pathologist C**	0.872 ± 0.024	0.850 ± 0.028	0.841 ± 0.027	0.865 ± 0.028	0.053	0.796
Task 3 - Atrophy	**GastritisMIL**	0.750 ± 0.031	0.725 ± 0.037	0.782 ± 0.031	0.731 ± 0.034	reference	reference
	**Junior Pathologist A**	0.611 ± 0.035	0.538 ± 0.038	0.554 ± 0.041	0.552 ± 0.036	0.011	0.698
	**Expert Pathologist B**	0.778 ± 0.032	0.750 ± 0.035	0.760 ± 0.033	0.785 ± 0.029	0.635	0.691
	**Expert Pathologist C**	0.822 ± 0.029	0.799 ± 0.033	0.813 ± 0.032	0.796 ± 0.033	0.079	0.814
Task 4 - IM	**GastritisMIL**	0.800 ± 0.031	0.719 ± 0.040	0.725 ± 0.040	0.728 ± 0.042	reference	reference
	**Junior Pathologist A**	0.833 ± 0.027	0.763 ± 0.036	0.767 ± 0.036	0.811 ± 0.031	0.48	0.877
	**Expert Pathologist B**	0.878 ± 0.024	0.832 ± 0.032	0.830 ± 0.031	0.848 ± 0.033	0.059	0.868
	**Expert Pathologist C**	0.861 ± 0.026	0.808 ± 0.035	0.798 ± 0.036	0.822 ± 0.036	0.135	0.895

Table S8. Accuracy, F1-score, precision, recall of our model (GastritisMIL) and human Pathologists in the external test cohort of XMHAH.

Data are represented as mean ± SD.

* Difference of accuracy between GastritisMIL and human Pathologists, tested by Paired chi-square test (McNamar’s test).

# inter-observer agreement of GastritisMIL and each human Pathologist, evaluated by quadratic weighted kappa coefficient.

Comparison of algorithm performance in recent CG-AI research								
**Research**	**Task 1 - Inflammation**				**Task 2 - Activity**			
	**Discrimination**	**AUC**	**Recall**	**Specificity**	**Discrimination**	**AUC**	**Recall**	**Specificity**
**LR**	NR	NR	NR	NR	NR	NR	NR	NR
**AMMNet**	NR	NR	NR	NR	whether activity	0.930	NR	NR
**GasMIL**	specific grade of Inflammation	0.970	0.860	0.860	specific grade of activity	0.981	0.900	0.900
**GastritisMIL (Ours)**	specific grade of Inflammation	**0.971**	**0.824**	**0.940**	specific grade of activity	**0.984**	**0.898**	**0.967**

**Research**	**Task 3 - Antrum Atrophy**				**Task 4 - Intestinal Metaplasia**			
	**Discrimination**	**AUC**	**Recall**	**Specificity**	**Discrimination**	**AUC**	**Recall**	**Specificity**
**LR**	moderate or severe atrophy	0.818	0.724	0.896	NR	NR	NR	NR
**AMMNet**	whether atrophy	0.970	NR	NR	whether IM	0.940	NR	NR
**GasMIL**	specific grade of atrophy	0.877	0.700	0.700	specific grade of IM	0.884	0.690	0.690
**GastritisMIL (Ours)**	specific grade of atrophy	**0.974**	**0.834**	**0.960**	specific grade of IM	**0.975**	**0.808**	**0.953**

Table S9. Comparison of algorithm performance in recent CG-AI research.
AUC = area under the receiver-operating characteristic curve. NR = not report.

Age-Stratified Performance Analysis Across Internal and External Cohorts								
**Age Group**	**Task 1 - Inflammation**				**Task 2 - Activity**			
	**XMUZSH (Internal)**	**FDUZSH (XM)**	**XMHCH**	**XMHAH**	**XMUZSH (Internal)**	**FDUZSH (XM)**	**XMHCH**	**XMHAH**
**Age < 45**	0.913 (24.9%)	0.889 (15.4%)	0.868 (31.2%)	0.818 (36.7%)	0.923 (52.0%)	0.833 (15.4%)	0.792 (31.2%)	0.909 (36.7%)
**45 ≤ Age ≤ 60**	0.886 (50.2%)	0.873 (53.8%)	0.822 (42.9%)	0.778 (35.0%)	0.925 (48.3%)	0.857 (53.8%)	0.781 (42.9%)	0.730 (35.0%)
**Age > 60**	0.880 (24.9%)	0.889 (30.7%)	0.795 (25.9%)	0.824 (28.3%)	0.916 (26.5%)	0.778 (30.7%)	0.909 (25.9%)	0.745 (28.3%)
**Average**	0.892	0.880	0.829	0.806	0.922	0.829	0.824	0.800
**Age Group**	**Task 3 - Antrum Atrophy**				**Task 4 - Intestinal Metaplasia**			
	**XMUZSH (Internal)**	**FDUZSH (XM)**	**XMHCH**	**XMHAH**	**XMUZSH (Internal)**	**FDUZSH (XM)**	**XMHCH**	**XMHAH**
**Age < 45**	0.872 (26.0%)	0.938 (20.5%)	0.660 (31.2%)	0.727 (36.7%)	0.896 (24.8%)	0.750 (20.5%)	0.849 (31.2%)	0.803 (36.7%)
**45 ≤ Age ≤ 60**	0.843 (47.8%)	0.811 (47.4%)	0.753 (42.9%)	0.746 (35.0%)	0.878 (48.7%)	0.892 (47.4%)	0.767 (42.9%)	0.857 (35.0%)
**Age > 60**	0.900 (26.2%)	0.760 (32.1%)	0.795 (25.9%)	0.784 (28.3%)	0.896 (26.4%)	0.800 (32.1%)	0.841 (25.9%)	0.725 (28.3%)
**Average**	0.866	0.821	0.735	0.750	0.896	0.833	0.812	0.800

Table S10. Age-stratified performance analysis across internal and three external cohorts.
Data are represented as accuracy (Proportion of the corresponding age group).

Option	External Testing		AUC	Accuracy	F1-score	Precision	Recall	NPV
Without Staining Normalization	Task 1 - Inflammation	FDUZSH (XM)	0.937 ± 0.018	0.880 ± 0.029	0.914 ± 0.021	0.936 ± 0.015	0.912 ± 0.020	1.000 ± 0.085
		XMHCH	0.924 ± 0.017	0.829 ± 0.029	0.812 ± 0.035	0.877 ± 0.027	0.775 ± 0.039	0.940 ± 0.022
		XMHAH	0.913 ± 0.017	0.806 ± 0.030	0.631 ± 0.050	0.880 ± 0.114	0.633 ± 0.033	0.921 ± 0.068
	Task 2 - Activity	FDUZSH (XM)	0.931 ± 0.018	0.829 ± 0.034	0.652 ± 0.061	0.621 ± 0.065	0.736 ± 0.040	1.000 ± 0.117
		XMHCH	0.923 ± 0.015	0.824 ± 0.029	0.785 ± 0.035	0.804 ± 0.034	0.800 ± 0.031	1.000 ± 0.064
		XMHAH	0.901 ± 0.017	0.800 ± 0.029	0.738 ± 0.036	0.754 ± 0.037	0.751 ± 0.032	1.000 ± 0.069
	Task 3 - Atrophy	FDUZSH (XM)	0.938 ± 0.019	0.821 ± 0.044	0.811 ± 0.049	0.810 ± 0.052	0.814 ± 0.049	0.940 ± 0.037
		XMHCH	0.865 ± 0.024	0.735 ± 0.034	0.693 ± 0.051	0.828 ± 0.046	0.664 ± 0.044	0.952 ± 0.049
		XMHAH	0.883 ± 0.018	0.750 ± 0.031	0.725 ± 0.037	0.782 ± 0.031	0.731 ± 0.034	0.831 ± 0.053
	Task 4 - IM	FDUZSH (XM)	0.952 ± 0.017	0.833 ± 0.042	0.816 ± 0.047	0.814 ± 0.048	0.819 ± 0.048	0.952 ± 0.008
		XMHCH	0.908 ± 0.016	0.812 ± 0.030	0.647 ± 0.065	0.754 ± 0.057	0.604 ± 0.064	0.970 ± 0.042
		XMHAH	0.928 ±0.015	0.800 ± 0.031	0.719 ± 0.040	0.725 ± 0.040	0.728 ± 0.042	0.924 ± 0.019
With Staining Normalization	Task 1 - Inflammation	FDUZSH (XM)	0.913 ± 0.021	0.761 ± 0.039	0.647 ± 0.076	0.689 ± 0.083	0.645 ± 0.087	0.973 ± 0.092
		XMHCH	0.925 ± 0.015	0.794 ± 0.031	0.766 ± 0.038	0.853 ± 0.024	0.729 ± 0.039	0.916 ± 0.032
		XMHAH	0.916 ± 0.017	0.744 ± 0.033	0.590 ± 0.049	0.857 ± 0.111	0.594 ± 0.033	0.921 ± 0.103
	Task 2 - Activity	FDUZSH (XM)	0.911 ± 0.028	0.829 ± 0.035	0.685 ± 0.068	0.674 ± 0.072	0.702 ± 0.074	0.972 ± 0.135
		XMHCH	0.911 ± 0.016	0.776 ± 0.033	0.717 ± 0.037	0.725 ± 0.038	0.735 ± 0.033	0.981 ± 0.065
		XMHAH	0.897 ± 0.018	0.783 ± 0.031	0.699 ± 0.036	0.711 ± 0.040	0.715 ± 0.030	1.000 ± 0.072
	Task 3 - Atrophy	FDUZSH (XM)	0.883 ± 0.030	0.692 ± 0.052	0.694 ± 0.056	0.696 ± 0.056	0.707 ± 0.055	0.967 ± 0.064
		XMHCH	0.803 ± 0.027	0.600 ± 0.038	0.549 ± 0.043	0.569 ± 0.043	0.554 ± 0.045	0.949 ± 0.062
		XMHAH	0.815 ± 0.020	0.556 ± 0.036	0.476 ± 0.040	0.605 ± 0.050	0.468 ± 0.034	0.884 ± 0.030
	Task 4 - IM	FDUZSH (XM)	0.883 ± 0.030	0.833 ± 0.042	0.812 ± 0.049	0.819 ± 0.048	0.820 ± 0.046	1.000 ± 0.035
		XMHCH	0.803 ± 0.027	0.718 ± 0.035	0.492 ± 0.068	0.647 ± 0.077	0.464 ± 0.062	0.964 ± 0.072
		XMHAH	0.815 ± 0.020	0.728 ± 0.033	0.632 ± 0.041	0.628 ± 0.040	0.644 ± 0.044	0.927 ± 0.030

Table S11. Impact of Staining Normalization on Model Performance Across External Test Cohorts.

Data are represented as mean ± SD. AUC = area under the receiver-operating characteristic curve

Age Distribution Analysis Across Internal and External Cohorts						
**Cohort**	**Task 1 - Inflammation**			**Task 3 - Antrum Atrophy**		
	**Age < 45**	**45 ≤ Age ≤ 60**	**Age > 60**	**Age < 45**	**45 ≤ Age ≤ 60**	**Age > 60**
**Training Cohort**	25.6%	48.8%	25.6%	23.6%	48.9%	27.5%
**Internal Test Cohort**	25.0%	50.0%	25.0%	26.0%	48.3%	25.7%
**FDUZSH (XM)**	15.4%	53.8%	30.8%	15.4%	53.8%	30.8%
**XMHCH**	31.2%	42.9%	25.9%	31.2%	42.9%	25.9%
**XMHAH**	35.7%	35.0%	28.3%	35.7%	35.0%	28.3%
**Cohort**	**Task 2 - Activity**			**Task 4 - Intestinal Metaplasia**		
	**Age < 45**	**45 ≤ Age ≤ 60**	**Age > 60**	**Age < 45**	**45 ≤ Age ≤ 60**	**Age > 60**
**Training Cohort**	25.6%	48.6%	25.7%	25.6%	49.1%	25.4%
**Internal Test Cohort**	23.9%	48.3%	27.8%	26.1%	45.8%	28.1%
**FDUZSH (XM)**	20.5%	47.4%	32.1%	20.5%	47.4%	32.1%
**XMHCH**	31.2%	42.9%	25.9%	31.2%	42.9%	25.9%
**XMHAH**	36.7%	35.0%	28.3%	36.7%	35.0%	28.3%

Table S12. Age Distribution Analysis Across Internal and External Cohorts.

Label Distribution Analysis Across Internal and External Cohorts							
**Cohort**	**Task 1 - Inflammation**			**Task 3 - Antrum Atrophy**			
	**Normal to Mild**	**Moderate**	**Severe**	**Normal**	**Mild**	**Moderate**	**Severe**
**Training Cohort**	30.9%	60.8%	8.3%	37.1%	39.0%	19.3%	4.6%
**Internal Test Cohort**	31.0%	60.8%	8.2%	37.5%	38.6%	19.3%	4.6%
**FDUZSH (XM)**	45.30%	50.4%	4.3%	15.4%	32.1%	37.2%	15.4%
**XMHCH**	29.4%	54.1%	16.5%	34.7%	32.9%	25.3%	54.1%
**XMHAH**	53.3%	37.8%	8.9%	14.4%	23.9%	21.7%	40.0%
**Cohort**	**Task 2 - Activity**			**Task 4 - Intestinal Metaplasia**			
	**Normal to Mild**	**Moderate**	**Severe**	**Normal**	**Mild**	**Moderate**	**Severe**
**Training Cohort**	61.8%	21.1%	17.1%	66.4%	14.4%	11.7%	7.5%
**Internal Test Cohort**	61.7%	21.1%	17.1%	66.5%	14.6%	11.5%	7.4%
**FDUZSH (XM)**	78.6%	14.5%	6.8%	38.5%	17.9%	23.1%	20.5%
**XMHCH**	51.8%	20.6%	27.6%	66.5%	22.4%	5.9%	5.3%
**XMHAH**	50.0%	18.9%	31.1%	49.4%	10.0%	24.4%	16.1%

Table S13. Label Distribution Analysis Across Internal and External Cohorts.

Cohort		Task 1 & Compression Error		Task 3 & Structure Confusion Error		Task 4 & Structure Confusion Error	
		Proportion	P-value*	Proportion	P-value*	Proportion	P-value*
External Cohort	FDUZSH (XM)	42.9% (6/14)	1.000	61.1% (11/18)	0.430	46.2% (6/13)	0.776
	XMHCH	44.8% (13/29)	0.757	57.8% (26/45)	0.333	45.5% (15/33)	0.450
	XMHAH	54.3% (19/35)	0.163	51.1% (23/45)	0.864	47.2% (17/36)	0.547
Internal Cohort	XMUZSH	39.8% (68/171)	reference	48.3% (84/174)	reference	54.3% (107/197)	reference

Table S14. Proportions of Error Types Among Total Failure Cases in Different Tasks Across Multi-center Cohorts.

* Difference of error proportion between XMUZSH cohort and another external cohort, tested by Chi-square test.
